# Facile synthesis of indolo[3,2-*a*]carbazoles via Pd-catalyzed twofold oxidative cyclization

**DOI:** 10.3762/bjoc.12.243

**Published:** 2016-11-22

**Authors:** Chao Yang, Kai Lin, Lan Huang, Wei-dong Pan, Sheng Liu

**Affiliations:** 1 Guizhou University, Guiyang, PR China; 2 The Key Laboratory of Chemistry for Natural Products of Guizhou Province, Chinese Academy of Sciences, Guiyang, PR China

**Keywords:** cyclization, indolo[3,2-*a*]carbazole, N-arylation, oxidative biaryl coupling, palladium catalysis

## Abstract

A rapid and efficient route has been developed for the synthesis of 9-methoxycarbonylindolo[3,2-*a*]carbazole derivatives. The key steps in this approach involved an aromatic amination and an oxidative biaryl coupling. Via the present route, indolo[3,2-*a*]carbazole derivatives are available in 3–4 steps based on commercially available starting materials.

## Introduction

**Figure 1:**
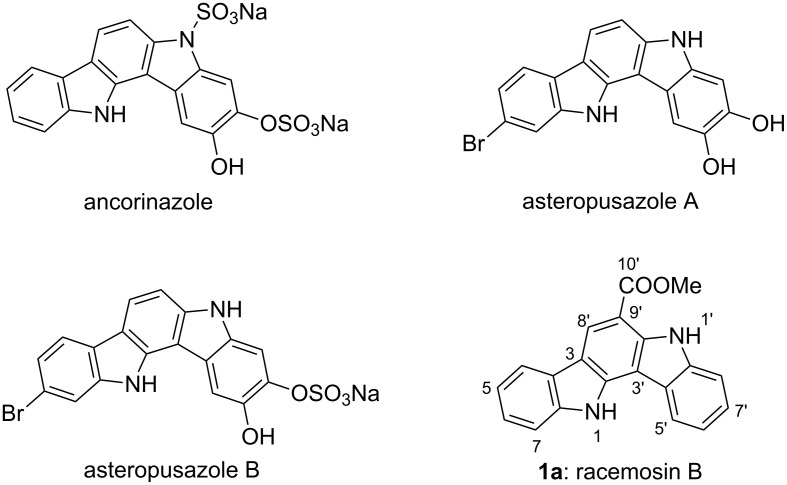
Natural indolo[3,2-*a*]carbazole alkaloids.

Many biologically active carbazole derivatives are of interest as drug-like templates. The indolo[3,2-*a*]carbazole skeleton, one of the isomeric indolocarbazoles, was firstly identified from nature in 2002 [1]. Up to now, four members of this family were isolated from marine organisms (Figure 1) [1–3]. Preliminary results of the antimicrobial and cytotoxicity assays indicated that these compounds have medicinal potentiality to target neurological and psychiatric disorders [1–2].

Several methods for the construction of the indolo[3,2-*a*]carbazole skeleton were developed by Bergman [4–5], Cachet [6], and Zhiping Li [7]. Very recently, we also reported a novel strategy toward indolo[3,2-*a*]carbazoles [8], and the key transformations involved a biomimetic transamination/aromatic cyclization sequence to deliver 9-methoxycarbonyl indolo[3,2-*a*]carbazole derivatives. Previous literature aimed at providing rapid and efficient access to indolo[3,2-*a*]carbazoles, however, most of them have some disadvantages. Few methods were reported to be suitable for scale-up preparation and the introduction of substituents to aromatic rings was also inconvenient. It could still be necessary to exploit more practical methods and economic procedures to assemble these polycyclic molecules. In this paper, we describe a concise, 3 to 4 steps procedure for the synthesis of indolo[3,2-*a*]carbazoles via palladium-catalyzed twofold oxidative cyclization.

## Findings

The oxidative biaryl coupling reaction was originally described over 30 years ago using stoichiometric palladium(II) [9–10]. Quite recently, the reaction has been developed to proceed catalytically and widely applied as an atom economic approach to carbazoles [11–12].

**Scheme 1:**
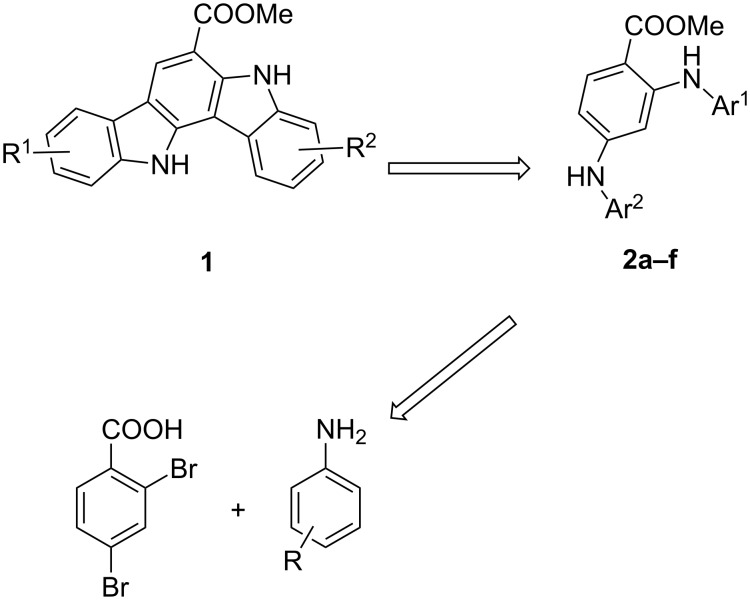
Retrosynthetic analysis of indolo[3,2-*a*]carbazoles.

As an interest concerning the development of synthesis methods for carbazoles, we considered that indolo[3,2-*a*]carbazoles **1** which represents racemosin B and its analogues also could be conveniently delivered by bidirectional indole annulations from methyl 2,4-dianilinobenzoates **2** (Scheme 1). Further disconnection of compounds **2** provided two commercially available basic fragments: 2,4-dibromobenzoic acid and anilines. Bergman et al. have described a synthetic route to indolo[3,2-*b*]carbazole by adopting twofold oxidative biaryl coupling as a key step [13], and the method has been further applied to the synthesis of malasseziazoles by Knölker et al. [14]. However, the main challenge in retrosynthetic analysis was to achieve the regioselective cyclizaion, since the formation of undesired regioisomeric monocyclized products would block the further cyclization. If the reaction follows the designed sequence, it would allow us to establish a novel and simple route to indolo[3,2-*a*]carbazole derivatives.

**Scheme 2:**
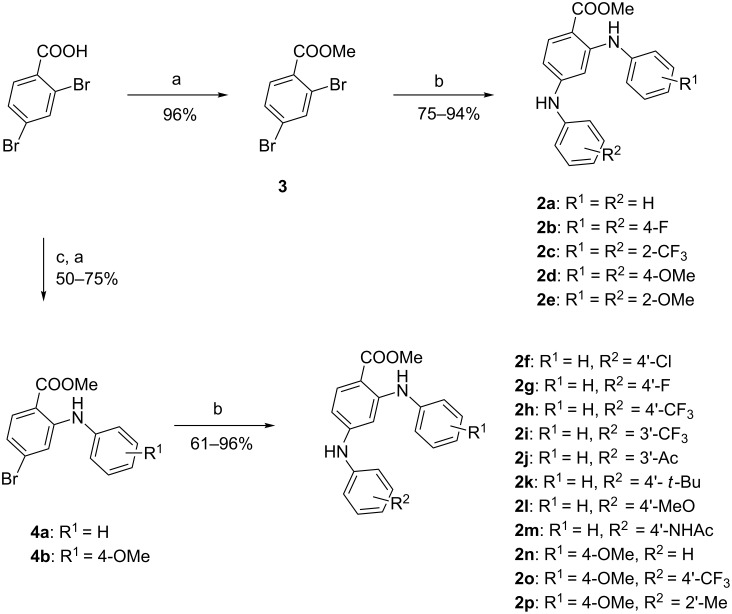
Reagents and conditions: (a) H_2_SO_4_, MeOH; (b) Ar-NH_2_, Pd(OAc)_2_, BINAP, dioxane, 100 °C; (c) 5 mol % Cu_2_O, K_3_PO_4_, DMA, 70 °C.

Our investigation started with the preparation of methyl 2,4-dianilinobenzoates (**2a–e**, Scheme 2). The double Buchwald–Hartwig coupling of methyl 2,4-dibromobenzoate **3** (obtained by treatment of dibromobenzoic acid with sulfuric acid in methanol) with an excess of anilines in the presence of cesium carbonate and catalytic quantities of Pd(OAc)_2_ and BINAP were processed smoothly. The desired methyl 2,4-dianilinobenzoates (**2a–e**) were obtained in good to excellent yield. In a parallel synthesis, using the Houpis protocol [15], Cu-catalyzed selective monoaminations of the 2,4-dibromobenzoic acid at the 2-position were achieved. After methyl esterification of ortho-aminated benzoic acids, substrates **4a** and **b** were treated with other anilines under the previous Buchwald–Hartwig conditions to afford methyl 2,4-dianilinobenzoate (**2f–p**) with various substituents at the aniline moieties.

Given the ease with which methyl 2,4-dianilinobenzoates could be assembled, further efforts were focused on the optimization studies of the key bidirectional indole annulations using **2a** as the model substrate. To maximize efficiency and technical simplicity, we prefer to employ air at ambient pressure as the terminal oxidant, and hence only the combination of bases (K_2_CO_3_ and NaO*t*-Bu) and solvents (AcOH, TFA, PivOH) were screened to promote the demanding transformation (Table 1). Initial investigations were performed with **2a** and catalytic Pd(OAc)_2_ in AcOH at 110 °C under air (Table 1, entries 1 and 2). To our delight, the desired product could be formed without observing any byproduct, and the adoption of NaO*t*-Bu as base gave a better yield. We next replaced the acetic acid with other acidic solvents. The desired transformation could not take place in TFA (Table 1, entry 3), however, PivOH [16] was found to be the preferred solvent (Table 1, entry 6). Moreover, the 10 mol % of palladium was required for complete conversion of **2a** within 48 h (Table 1, entry 7). Since the only intermediate **5** could be detected and isolated, it indicated that the two cyclizations of **1a** did not happen simultaneously but followed a particular sequence. Further attempts to shorten the reaction time by enhancing the temperature from 120 °C to 140 °C significantly decreased the yield (Table 1, entry 8), on account of decomposition of both the starting material and the product may occur. To examine whether the desired transformation was suitable for scale-up, a gram-scale synthesis of **2a** was also achieved (Table 1, entry 9).

**Table 1:** Optimization studies for annulation conditions.^a^

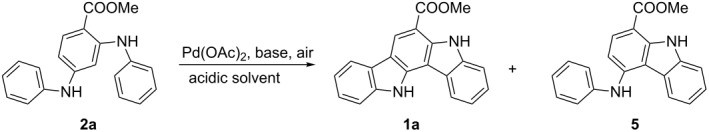					

entry	[Pd]	base (equiv)	solvent	*t* (°C)	yield^b^

1	10 mol %	K_2_CO_3_ (0.1)	AcOH	110	58%
2	10 mol %	NaO*t*-Bu (0.1)	AcOH	110	68%
3	10 mol %	NaO*t*-Bu (0.1)	TFA	90	0%
4	10 mol %	K_2_CO_3_ (0.1)	AcOH	120	0%^c^
5	10 mol %	K_2_CO_3_ (0.1)	PivOH	120	51%
6	10 mol %	NaO*t*-Bu (0.1)	PivOH	120	85%
7	5 mol %	NaO*t*-Bu (0.1)	PivOH	120	35%^d^
8	10 mol %	NaO*t*-Bu (0.1)	PivOH	140	45%
9	10 mol %	NaO*t*-Bu (0.1)	PivOH	120	69%^e^

^a^A mixture of compound **2a** (150 mg, 0.47 mmol), Pd(OAc)_2_ and basein 0.5 mL solvent was stirred and heated for 48 h under air (entries 1–3, 5–8). ^b^Isolated yield of **1a**. ^c^Reaction was carried out under Ar. ^d^Intermediate **5** was isolated in 42% yield from entry 7. ^e^Compound **2a** (1.5 g, 3.14 mmol) with the catalyst in 7.5 mL PivOH was stirred for 72 h (entry 9).

**Scheme 3:**
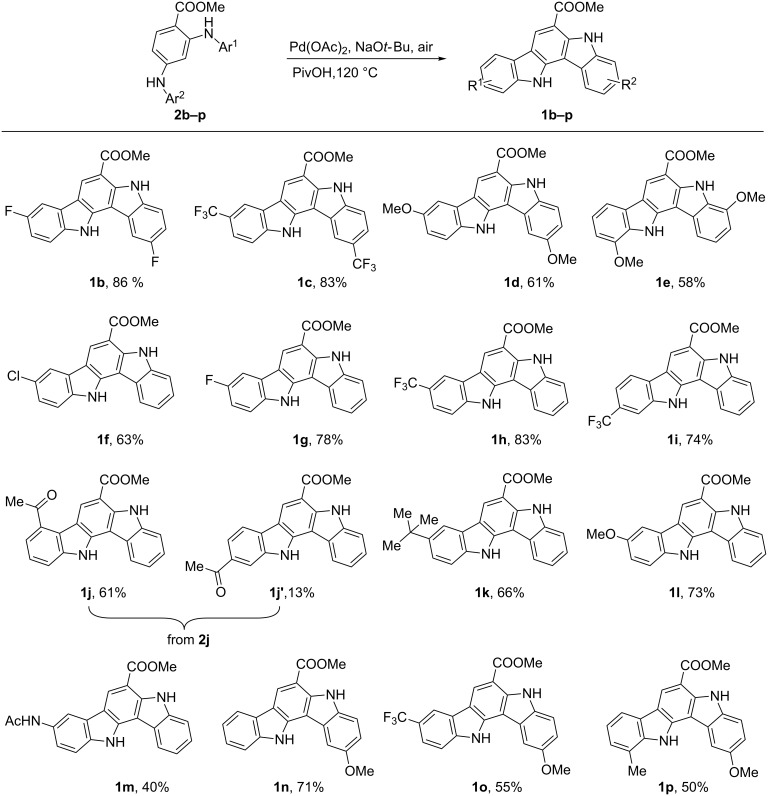
Substrate scope for Pd-catalyzed twofold annulations.

With the optimized conditions in hand, the generality and scope of the reaction were also examined (Scheme 3). A range of precursors (**2b–p**) with various substituents were cyclized to afford the desired products in moderate to good yields. Electron-withdrawing groups (CF_3_, F) gave the highest yields. The introduction of electron-donating substituents such as methoxy and acetylamino groups significantly reduced the reactivity, thus the complete cyclization needed longer time and the yield was relatively lower. Moreover, a chlorine atom at the aromatic ring was tolerated under these conditions.

The meta-substituted starting materials **2i** and **2j** gave good regioselectivity. When a trifluoromethyl group was located at the meta-position, only one regioisomer **1i** was observed. However, the selectivity was reversed when acetyl was present at the meta-position (5:1 for **2j**). The main cyclized product of **2j** was the corresponding ortho-acetyl product **1j**. Unexpectedly, the reaction preferentially occurred at a more sterically hindered position.

## Conclusion

In conclusion, a twofold C−H activation protocol applied to methyl 2,4-dianilinobenzoates facilitated a short and quick access to the cyclized products. Via the present route, indolo[3,2-*a*]carbazole derivatives are available in 3–4 steps based on commercially available starting materials. The operational simplicity combined with the convenience for introducing substituents to the aromatic rings makes this method useful.

## Supporting Information

File 1 Experimental part and NMR spectra of synthesized compounds.
