# Supplementary material for: Facile synthesis of indolo[3,2-a]carbazoles via Pd-catalyzed twofold oxidative cyclization
Source: Beilstein J Org Chem. 2016 Nov 22;12:2490–4. doi: 10.3762/bjoc.12.243 (PMC5238593; doi:10.3762/bjoc.12.243)
Supplement: File 1 — Experimental part and NMR spectra of synthesized compounds. [file Beilstein_J_Org_Chem-12-2490-s001.pdf]

Supporting Information

for

Facile synthesis of indolo[3,2-*a*]carbazoles via Pd-catalyzed twofold oxidative cyclization

Chao Yang[1], Kai Lin[2], Lan Huang[2], Wei-dong Pan[2] and Sheng Liu*[2]

Address: [1]Guizhou University, Guiyang, PR China and [2]The Key Laboratory of Chemistry for Natural Products of Guizhou Province, Chinese Academy of Sciences, Guiyang, PR China

Email: Sheng Liu - lsheng@126.com

*Corresponding author

Experimental part and NMR spectra of synthesized compounds

1. General description.

2. Experimental and spectroscopic data for compounds 3, 4a, 4b, 2a–p, 1a–p.

3. NMR Spectra for compounds 3, 4a, 4b, 2a–p, 5, 1a–p.

1. General description.

Starting materials, reagents and solvents were purchased from commercial suppliers and used without further purification. The progress of reactions was monitored by silica gel thin layer chromatography (TLC) plates, visualized under UV. Products were purified by flash column chromatography (FCC) on 200–300 mesh silica gel. Proton nuclear magnetic resonance spectra (^{1}H NMR) were recorded on a spectrometer operating at 400 or 500 MHz. Data are reported as follows: chemical shift, integration, multiplicity (s = singlet, d = doublet, dd =double doublet, t = triplet, br = broad, m = multiplet). Carbon nuclear magneticresonance spectra (^{13}C NMR) were recorded on a spectrometer operating at 100 or 125 MHz.

2. Experimental and spectroscopic data for compounds 3, 4a, 4b, 2a–p, 1a–p.

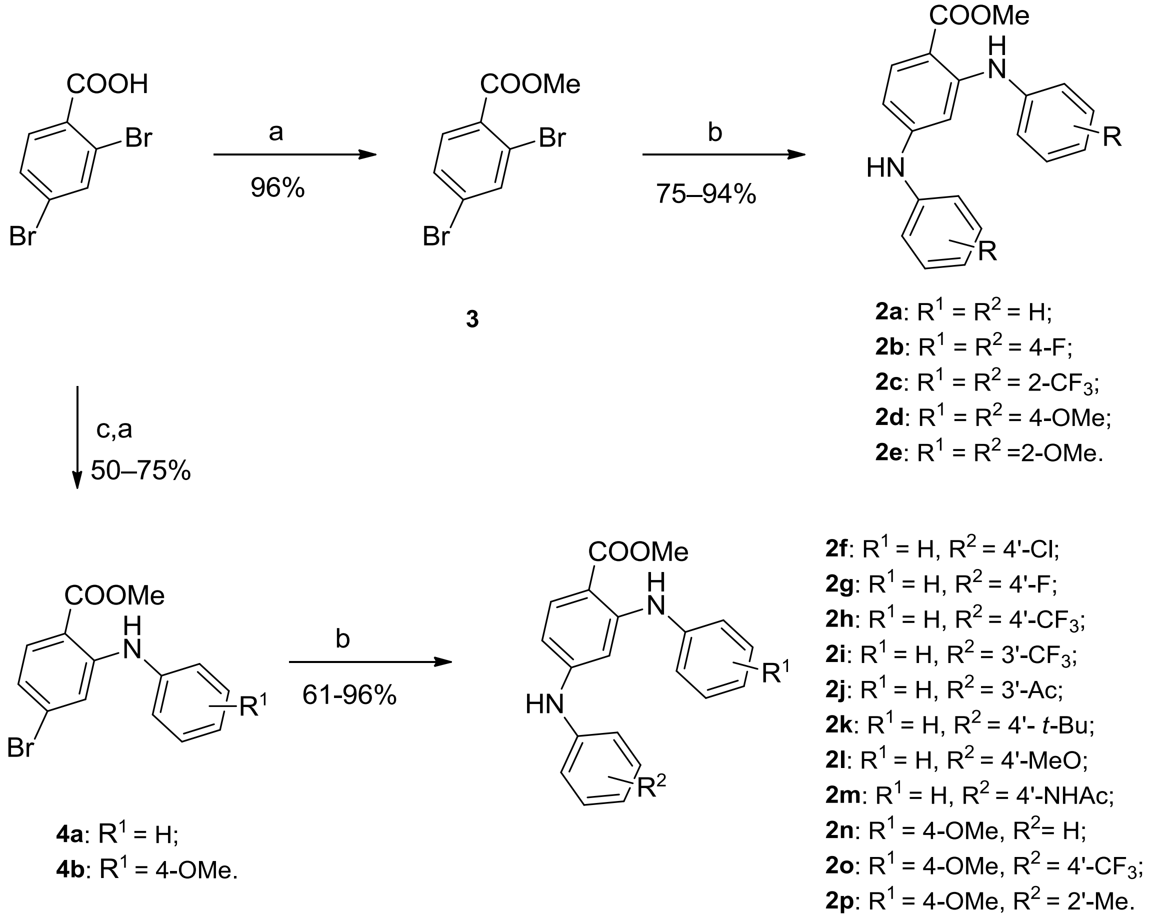

General procedure a: The corresponding acid (5 mmol) was dissolved in CH_3OH (20 mL), and then H_2SO_4 (8 equiv) was added at 0 °C, the reaction mixture was then refluxed for 24–48 h. After cooling, the solvent was evaporated. To the resulting mixture was slowly added a solution of 10% Na_2CO_3 (200 mL), and then the aqueous solution was extracted with ethyl acetate. The organic layers were combined, dried over anhydrous Na_2SO_4, filtered and concentrated in vacuo. The crude product was purified by column chromatography to obtain compound **3** or compound **4a** and **4b**.

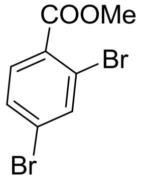

3

1.4 g; isolated yield 96%; white solid; ^{1}H NMR (400 MHz, $CDCl_3$) δ 7.83 (d, J = 2.0 Hz, 1H), 7.68 (d, J = 8.4 Hz, 1H), 7.49 (dd, J = 8.4, 2.0 Hz, 1H), 3.92 (s, 3H); ^{13}C NMR (125 MHz, $CDCl_3$) δ 165.8, 136.9, 132.6, 130.7, 130.5, 126.6, 122.8, 52.7. HRMS (ESI) m/z calcd for $C_8H_7Br_2O_2$ $(M+1)^+$ 294.8792, found 294.8779.

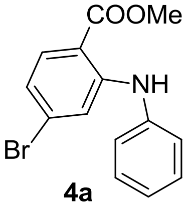

4a

765 mg; isolated yield 50% (2 steps from 2,4-dibromobenzoic acid); yellow solid; IR (KBr) 3312, 2955, 1687, 1595, 1576, 1428, 1240, 1098, 763; ^{1}H NMR (400 MHz, $CDCl_3$) δ 9.51 (s, 1H), 7.78 (d, J = 8.8 Hz, 1H), 7.37 (t, J = 8.0 Hz, 2H), 7.33 (d, J = 1.6 Hz, 1H), 7.23 (d, J = 8.0 Hz, 2H), 7.14 (t, J = 7.2 Hz, 1H), 6.81 (dd, J = 8.4, 2.0 Hz, 1H), 3.88 (s, 3H); ^{13}C NMR (100 MHz, $CDCl_3$) δ 168.4, 148.9, 139.7, 132.8, 129.5, 129.2, 124.4, 123.1, 120.0, 116.1, 110.2, 51.8. HRMS (ESI) m/z calcd for $C_{14}H_{13}BrNO_2$ $(M+1)^+$ 306.0130, found 306.0140.

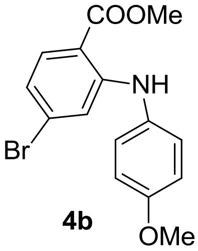

4b

1.26 g; isolated yield 75% (2 steps from 2,4-dibromobenzoic acid); yellow solid; IR (KBr) 3310, 2957, 1682, 1597, 1575, 1425, 1244, 1092, 762; ^{1}H NMR (500 MHz, $CDCl_3$) δ 9.31 (s, 1H), 7.77 (d, J = 8.5 Hz, 1H), 7.15 (d, J = 8.5 Hz, 2H), 7.05 (d, J = 1.5 Hz, 1H), 6.93 (d, J = 8.5 Hz, 2H), 6.76 (dd, J = 8.5, 2.0 Hz, 1H), 3.89 (s, 3H), 3.83 (s, 3H). ^{13}C NMR (125 MHz, $CDCl_3$) δ 168.5, 157.2, 150.5, 132.8, 132.3, 129.4, 126.4, 119.2, 115.7, 114.8, 109.4, 55.5, 51.8; HRMS (ESI) m/z calcd for $C_{15}H_{15}BrNO_3$ $(M+1)^+$ 336.0235, found 336.0241.

General procedure b: To a solution of benzoic acid methyl ester (**3**, **4a**,**b**) (500 mg), the respective arylamine compounds (2.4 equiv for **3**, 1.2 equiv for **4a**,**b**), Cs_2CO_3 (2.8 equiv for **3**, 1.4 equiv for **4a**,**b**), BINAP (0.08 equiv) in 1,4-dioxane (5 mL) was added $Pd(OAc)_2$ (5 mol %) under nitrogen. And the reaction mixture was stirred at 100 °C for 10–24 h. The reaction mixture was cooled to room temperature, and then H_2O was added. The mixture was extracted with ethyl acetate, and then the organic layers were combined, dried over anhydrous Na_2SO_4, filtered and concentrated in vacuo. The crude product was purified by column chromatography.

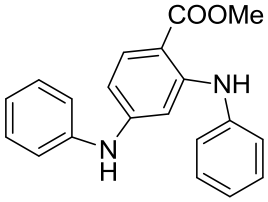

2a

406 mg; isolated yield 75%; white solid; IR (KBr) 3386, 2924, 1670, 1613, 1577, 1236, 1098, 732;

^{1}H NMR (400 MHz, $CDCl_3$) δ 9.58 (s, 1H), 7.84 (d, J = 8.8 Hz, 1H), 7.34-7.22 (m, 6H), 7.11-6.97 (m, 4H), 6.76 (d, J = 2.0 Hz, 1H), 6.36 (dd, J = 8.8, 2.0 Hz, 1H), 5.90 (s, 1H), 3.85 (s, 3H). ^{13}C NMR (100 MHz, $CDCl_3$) δ 168.6, 149.6, 148.9, 140.7, 140.6, 133.4, 129.3, 129.2, 123.5, 122.8, 122.7, 120.4, 105.4, 103.9, 98.2, 51.3; HRMS (ESI) m/z calcd for $C_{20}H_{19}N_2O_2$ $(M+1)^+$ 319.1447, found 319.1459.

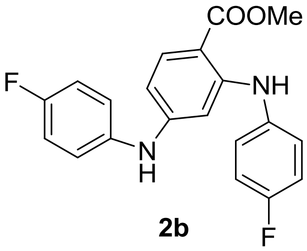

2b

566 mg; isolated yield 94%; yellow solid; IR (KBr) 3360, 3320, 2949, 1668, 1615, 1506, 1435, 1271, 1098, 771; ^{1}H NMR (500 MHz, $CDCl_3$) δ 9.45 (s, 1H), 7.82 (d, J = 8.5 Hz, 1H), 7.15 (dd, J = 10.0, 5.0 Hz, 2H), 7.06-6.95 (m, 6H), 6.44 (d, J = 2.5 Hz, 1H), 6.23 (d, J = 9.0, 2.0 Hz, 1H), 5.88-5.85 (m, 1H), 5.52 (d, J = 23.1 Hz, 1H), 2.92 (d, J = 2.0 Hz, 3H). ^{13}C NMR (100 MHz, $CDCl_3$) δ 168.6, 159.4 (d, J_{C-F} = 242.8 Hz), 158.9 (d, J_{C-F} = 242.3 Hz), 150.3, 149.7, 136.5 (t, J_{C-F} = 2.3 Hz), 133.4, 125.3 (d, J_{C-F} = 8.2 Hz), 123.2 (d, J_{C-F} = 8.0 Hz), 116.0 (d, J_{C-F} = 22.6 Hz), 115.9 (d, J_{C-F} = 22.5 Hz), 104.6, 103.4, 97.0, 51.3. HRMS (ESI) m/z calcd for $C_{20}H_{17}F_2N_2O_2$ $(M+1)^+$ 355.1258, found 355.1254.

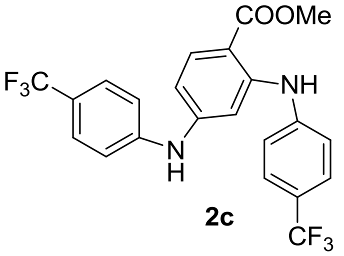

2c

703 mg; isolated yield 91%; white solid; IR (KBr) 3361, 3326, 2953, 1652, 1602, 1521, 1269, 1068, 829; ^{1}H NMR (400 MHz, $CDCl_3$) δ 9.80 (s, 1H), 7.92 (d, J = 8.8 Hz, 1H), 7.54 (t, J = 8.4 Hz, 4H), 7.28 (d, J = 8.4 Hz, 2H), 7.15 (d, J = 8.0 Hz, 2H), 6.94 (s, 1H), 6.57 (d, J = 8.8 Hz, 1H), 6.26 (s, 1H), 3.88 (s, 3H). ^{13}C NMR (125 MHz, $CDCl_3$) δ 168.4, 147.9, 147.3, 144.2, 144.1, 133.6, 126.7 (q, J_{C-F} = 3.8 Hz), 126.6 (q, J_{C-F} = 3.8 Hz), 124.5 (q, J_{C-F} = 200.1 Hz), 124.3, 124.0, 123.9 (q, J_{C-F} = 181.3 Hz), 123.2, 123.2, 120.6, 118.3, 107.5, 106.5, 101.2, 51.7. HRMS (ESI) m/z calcd for $C_{22}H_{17}F_6N_2O_2$ $(M+1)^+$ 455.1194, found 455.1182.

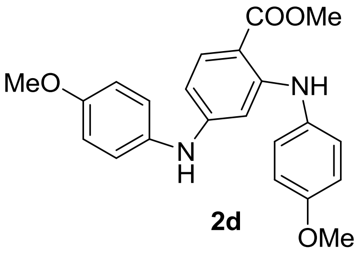

2d

572 mg; isolated yield 89%; yellow oil; IR (KBr) 3307, 2948, 1671, 1594, 1511, 1263, 1091, 770; ^{1}H NMR (400 MHz, $CDCl_3$) δ 9.38 (s, 1H), 7.79 (d, J = 8.8 Hz, 1H), 7.15 (d, J = 8.4 Hz, 2H), 7.04 (d, J = 8.4 Hz, 2H), 6.87 (d, J = 8.4 Hz, 2H), 6.83 (d, J = 8.4 Hz, 2H), 6.37 (s, 1H), 6.17 (d, J = 8.4 Hz, 1H), 5.73 (s, 1H), 3.84 (s, 3H), 3.80 (s, 3H), 3.78(s, 3H). ^{13}C NMR (125 MHz, $CDCl_3$) δ 168.7, 156.4, 156.1, 151.1, 150.5, 133.5, 133.3, 125.8, 123.9, 114.4, 103.7, 102.3, 96.3, 77.3, 77.0, 76.8, 55.39, 51.10. HRMS (ESI) m/z calcd for $C_{22}H_{23}N_2O_4$ $(M+1)^+$ 379.1658, found 379.1662.

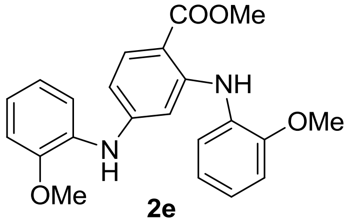

2e

565 mg; isolated yield 88%; yellow oil; IR (KBr) 3339, 2947, 1676, 1591, 1519, 1243, 1089, 746; ^{1}H NMR (500 MHz, $CDCl_3$) δ 9.63 (s, 1H), 7.90 (d, J = 9.0 Hz, 1H), 7.48 (d, J = 8.0 Hz, 1H), 7.39 (dd, J = 7.5, 1.0 Hz, 1H), 7.04 (t, J = 7.5 Hz, 1H), 6.99 (d, J = 2.0 Hz, 1H), 6.97-6.88 (m, 4H), 6.48 (dd, J = 9.0, 2.0 Hz, 1H), 6.32 (s, 1H), 3.90 (s, 6H), 3.84 (s, 3H).^{13}C NMR (125 MHz, $CDCl_3$) δ 168.3, 151.4, 149.3, 148.8, 148.3, 133.2, 130.4, 129.9, 123.1, 121.8, 120.9, 120.5, 120.2, 117.9, 111.1, 110.6, 106.2, 104.7, 99.0, 55.6, 55.4, 51.2. HRMS (ESI) m/z calcd for $C_{22}H_{23}N_2O_4$ $(M+1)^+$ 379.1658, found 379.1667.

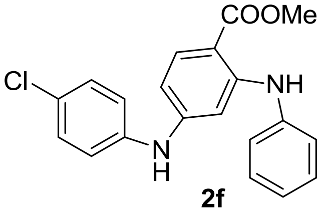

2f

365 mg; isolated yield 61 %; white solid; IR (KBr) 3360, 3335, 2954, 1651, 1615, 1516, 1266, 1091, 749; ^{1}H NMR (500 MHz, $CDCl_3$) δ 9.57 (s, 1H), 7.84 (d, J = 9.0 Hz, 1H), 7.32 (t, J = 9.6 Hz, 2H), 7.24-7.20 (m, 4H), 7.07 (t, J = 8.0 Hz, 1H), 7.00 (d, J = 8.5 Hz, 2H), 6.71 (d, J = 1.5 Hz, 1H), 6.31 (dd, J = 8.5, 1.5 Hz, 1H), 5.85 (s, 1H), 3.85 (s, 3H). ^{13}C NMR (125 MHz, $CDCl_3$) δ 168.5, 149.7, 148.4, 140.6, 139.4, 133.4, 129.5, 129.3, 127.5, 123.7, 122.8, 122.4, 121.4, 105.4, 104.3, 51.4. HRMS (ESI) m/z calcd for $C_{20}H_{18}ClN_2O_2$ $(M+1)^+$ 353.1057, found 353.1060.

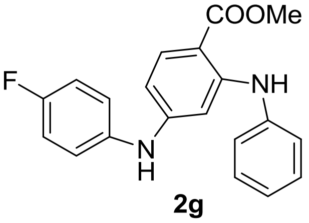

2g

548 mg; isolated yield 96%; light yellow solid; IR (KBr) 3357, 2925, 1656, 1619, 1508, 1351, 1092, 765; ^{1}H NMR (400 MHz, $CDCl_3$) δ 9.58 (s, 1H), 7.82 (d, J = 8.4 Hz, 1H), 7.31 (t, J = 8.0 Hz, 2H), 7.21 (d, J = 8.0 Hz, 2H), 7.08-7.04 (m, 3H), 6.98 (t, J = 8.8 Hz, 2H), 6.65 (d, J = 2.0 Hz, 1H), 6.25 (dd, J = 8.8, 2.4 Hz, 1H), 5.80 (s, 1H), 3.85 (s, 3H). ^{13}C NMR (100 MHz, $CDCl_3$) δ 168.6, 159.0 (d, $J_{C\text{-}F}$ = 242.4 Hz), 149.6, 149.6, 140.6, 136.6 (d, $J_{C\text{-}F}$ = 3.0 Hz), 133.4, 129.2, 123.5, 123.2,

123.2, 122.7, 116.1, 115.9, 104.8, 103.7, 97.5, 51.3. HRMS (ESI) m/z calcd for $C_{20}H_{18}FN_2O_2$ $(M+1)^+$ 337.1352, found 337.1346.

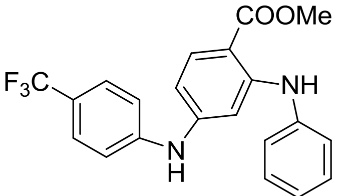

2h

551 mg; isolated yield 84%; yellow solid; IR (KBr) 3347, 2950, 1650, 1605, 1541, 1328, 1286, 1070, 825; ^1H NMR (500 MHz, $CDCl_3$) δ 9.58 (s, 1H), 7.89 (d, J = 8.5 Hz, 1H), 7.49 (d, J = 8.5 Hz, 2H), 7.34 (t, J = 8.5 Hz, 2H), 7.23 (d, J = 7.5 Hz, 2H), 7.13-7.08 (m, 3H), 6.81 (d, J = 2.5 Hz, 1H), 6.45 (dd, J = 8.5 Hz, 2.0 Hz, 1H), 6.22-6.17 (br, 1H), 3.87 (s, 3H). ^{13}C NMR (125 MHz, $CDCl_3$) δ 168.5, 149.7, 147.1, 144.5, 140.4, 133.4, 129.4, 126.6 (q, J_{C-F} = 3.8 Hz), 124.3(q, J_{C-F} = 271.3 Hz), 123.9, 123.5, 123.1, 117.9, 106.5, 105.3, 100.2, 51.5. HRMS (ESI) m/z calcd for $C_{21}H_{18}F_3N_2O_2$ $(M+1)^+$ 387.1320, found 387.1315.

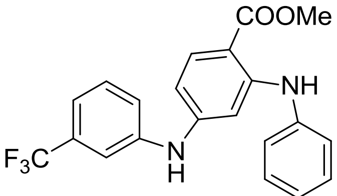

2i

531 mg; isolated yield 81%; white solid; IR (KBr) 3347, 2950, 1650, 1605, 1541, 1328, 1286, 1070, 825; ^1H NMR (500 MHz, $CDCl_3$) δ 9.60 (s, 1H), 7.88 (d, J = 8.5 Hz, 1H), 7.37-7.31 (m, 4H), 7.23-7.18 (m, 4H), 7.08 (t, J = 8.5 Hz, 1H), 6.79 (s, 1H), 6.34 (dd, J = 2.0, 9.0 Hz, 1H), 5.98 (s, 1H), 3.87 (s, 3H); ^{13}C NMR (125 MHz, $CDCl_3$) δ 168.5, 149.8, 147.7, 141.7, 140.4, 133.6, 131.9 (q, J_{C-F} = 33.8 Hz), 129.9, 129.4, 125.0 (q, J_{C-F} = 271.3 Hz), 123.9, 123.0, 122.6, 118.9, 118.8, 115.8, 115.7, 106.1, 104.9, 98.9, 51.5. HRMS (ESI) m/z calcd for $C_{21}H_{18}F_3N_2O_2$ $(M+1)^+$ 387.1320, found 387.1313.

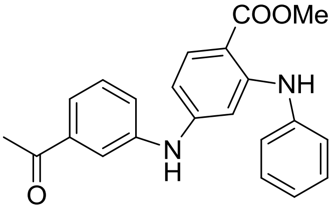

2j

514 mg; isolated yield 84%; white solid; IR (KBr) 3344, 1681, 1616, 1584, 1540, 1314, 1149, 1086, 696; 1H NMR (500 MHz, $CDCl_3$) δ 9.60 (s, 1H), 7.87 (d, J = 9.0 Hz, 1H), 7.71 (s, 1H), 7.55 (d, J = 7.0 Hz, 1H), 7.36 (t, J = 8.0 Hz, 1H), 7.34-7.23(m, 5H), 7.23 (s, 1H), 7.06 (t, J = 7.0 Hz, 1H), 6.80 (d, J = 2.0 Hz, 1H), 6.36 (dd, J = 9.0, 2.0 Hz, 1H), 6.03 (s, 1H), 3.87 (s, 3H), 2.55 (s, 3H); ^{13}C NMR (125 MHz, $CDCl_3$) δ 197.9, 168.8, 149.9, 148.4, 141.7, 140.7, 138.5, 133.7, 129.8, 129.5, 124.6, 123.9, 123.2, 122.8, 119.3, 106.0, 104.8, 98.8, 51.6, 26.9. HRMS (ESI) m/z calcd for $C_{22}H_{21}N_2O_3$ $(M+1)^+$ 361.1152, found 361.1159.

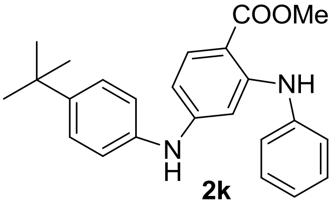

2k

566 mg; isolated yield 89%; white oil; IR (KBr) 3307, 2951, 1675, 1593, 1514, 1263, 1089, 772; ^{1}H NMR (400 MHz, $CDCl_3$) δ 9.61 (s, 1H), 7.83 (d, J = 8.8 Hz, 1H), 7.33-7.29 (m, 4H), 7.23 (d, J = 7.2 Hz, 1H), 7.07-7.03 (m, 3H), 6.72 (d, J = 2.0 Hz, 1H), 6.34 (dd, J = 8.8, 2.0 Hz, 1H), 5.86 (s, 1H), 3.85 (s, 3H), 1.31 (s, 9H). ^{13}C NMR (125 MHz, $CDCl_3$) δ 168.6, 149.5, 146.0, 140.8, 138.0, 133.3, 129.1, 126.0, 123.3, 122.6, 120.7, 105.1, 103.5, 97.8, 51.2, 34.2, 31.3. HRMS (ESI) m/z calcd for $C_{24}H_{27}N_2O_2$ $(M+1)^+$ 375.2073, found 375.2083.

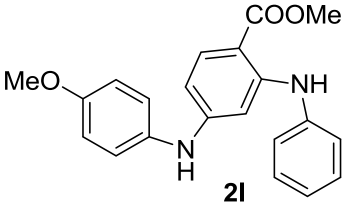

2l

443 mg; isolated yield 75%; white oil; IR (KBr) 3349, 2953, 1651, 1614, 1509, 1257, 1091, 1036, 750; ^{1}H NMR (500 MHz, $CDCl_3$) δ 9.69 (s, 1H), 7.88 (d, J = 8.0 Hz, 1H), 7.36 (t, J = 7.0 Hz, 2H), 7.28 (d, J = 7.5 Hz, 2H), 7.11 (d, J = 8.5 Hz, 3H), 6.90 (d, J = 8.5 Hz, 2H), 6.71 (d, J = 1.5 Hz, 1H), 6.27 (dd, J = 8.5, 1.5 Hz, 1H), 5.88 (s, 1H), 3.90 (s, 3H), 3.83 (s, 3H). ^{13}C NMR (125 MHz, $CDCl_3$) δ 168.6, 156.1, 150.5, 149.4, 140.7, 133.3, 129.1, 124.0, 123.1, 122.3, 114.5, 104.4, 102.9, 96.8, 55.3, 51.1. HRMS (ESI) m/z calcd for $C_{21}H_{21}N_2O_3$ $(M+1)^+$ 349.1552, found 349.1551.

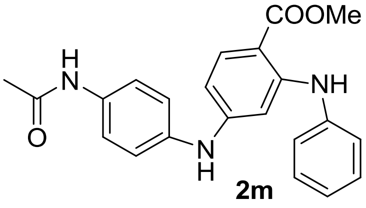

2m

567 mg; isolated yield 85%; light yellow powder; IR (KBr) 3308, 2948, 1670, 1594, 1511, 1263, 1091, 770; ^{1}H NMR (400 MHz, $CDCl_3$) δ 9.56 (s, 1H), 7.80 (d, J = 8.8 Hz, 1H), 7.68 (s, 1H), 7.36 (d, J = 8.4 Hz, 2H), 7.29 (t, J = 7.6 Hz, 2H), 7.19 (d, J = 7.6 Hz, 2H), 7.04-6.98 (m, 3H), 6.69 (s, 1H), 6.27 (d, J = 7.6 Hz, 1H), 6.02 (s, 1H), 3.83 (s, 3H), 2.11 (s, 3H). ^{13}C NMR (100 MHz, $CDCl_3$) δ 168.6, 168.6, 149.5, 149.2, 140.6, 137.0, 133.3, 133.0, 129.2, 123.3, 122.5, 121.3, 121.2, 105.1, 103.6, 97.7, 51.3, 24.2. HRMS (ESI) m/z calcd for $C_{22}H_{22}N_3O_3$ $(M+1)^+$ 376.1661, found 376.1674.

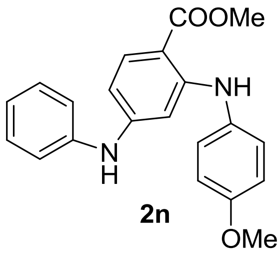

2n

533 mg; isolated yield 90%; yellow oil; IR (KBr) 3350, 2925, 1665, 1591, 1516, 1264, 1093, 746; ^{1}H NMR (400 MHz, $CDCl_3$) δ 9.37 (s, 1H), 7.81 (d, J = 8.8 Hz, 1H), 7.27 (d, J = 8.0 Hz, 1H), 7.24 (d, J = 4.4 Hz, 1H), 7.16 (d, J = 8.8 Hz, 2H), 7.07 (d, J = 8.0 Hz, 2H), 6.98 (t, J = 7.2 Hz, 1H), 6.88 (d, J = 8.8 Hz, 2H), 6.49 (d, J = 1.6 Hz, 1H), 6.32 (dd, J = 8.8, 2.0 Hz, 1H), 5.87 (s, 1H), 3.85 (s, 3H), 3.80 (s, 3H). ^{13}C NMR (100 MHz, $CDCl_3$) δ 168.6, 156.5, 151.2, 148.8, 140.8, 133.3, 133.3, 129.2, 126.0, 122.6, 120.2, 114.5, 104.7, 103.2, 97.7, 55.4, 51.3; HRMS (ESI) m/z calcd for $C_{21}H_{21}N_2O_3$ $(M+1)^+$ 349.1552, found 349.1546.

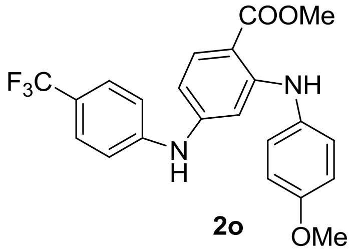

2o

488 mg; isolated yield 69%; yellow solid; IR (KBr) 3355, 2949, 1665, 1602, 1511, 1265, 2069, 832; ^{1}H NMR (400 MHz, $CDCl_3$) δ 9.37 (s, 1H), 7.87 (d, J = 8.4 Hz, 1H), 7.47 (d, J = 8.4 Hz, 2H), 7.16 (d, J = 8.8 Hz, 2H), 7.09 (d, J = 8.4 Hz, 2H), 6.90 (d, J = 8.8 Hz, 2H), 6.53 (d, J = 2.0 Hz, 1H), 6.40 (dd, J = 8.8, 2.0 Hz, 1H), 6.07 (s, 1H), 3.87 (s, 3H), 3.81 (s, 3H). ^{13}C NMR (100 MHz, $CDCl_3$) δ 168.5, 156.8, 151.3, 147.0, 144.5, 133.4, 133.0, 126.5 (q, J_{C-F} = 4.0 Hz), 126.3, 124.3 (q, J_{C-F} = 276.3 Hz), 123.4, 123.1, 117.8, 114.6, 105.7, 104.6, 99.7, 55.5, 51.4. HRMS (ESI) m/z calcd for $C_{22}H_{20}F_3N_2O_3$ $(M+1)^+$ 417.1426, found 417.1417.

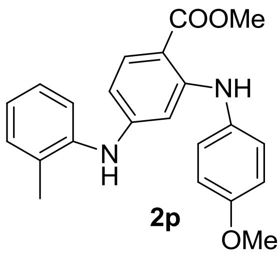

2p

504 mg; isolated yield 82 %; yellow oil; IR (KBr) 3436, 2930, 2856, 1681, 1587, 1220, 1082, 784; ^{1}H NMR (400 MHz, $CDCl_3$) δ 9.37 (s, 1H), 7.79 (d, J = 8.8 Hz, 1H), 7.24-7.14 (m, 5H), 7.00 (t, J = 7.6 Hz, 1H), 6.87 (d, J = 8.8 Hz, 2H), 6.34 (d, J = 2.0 Hz, 1H), 6.15 (dd, J = 8.8, 2.0 Hz, 1H), 5.52 (s, 1H), 3.84 (s, 3H), 3.80 (s, 3H), 2.20 (s, 3H). ^{13}C NMR (100 MHz, $CDCl_3$) δ 168.7, 156.5, 151.2, 150.0, 138.9, 133.4, 133.3, 131.0, 126.6, 125.9, 124.0, 122.5, 114.5, 104.4, 102.8, 97.3, 55.5, 51.2, 17.8; HRMS (ESI) m/z calcd for $C_{22}H_{23}N_2O_3$ $(M+1)^+$ 363.1709, found 363.1701.

General procedure c: 2,4-Dibromobenzoic acid (4.0 g, 14.3 mmol), anilines (21.4 mmo) and K_3PO_4 (7.6 g, 28.6 mmol, 2 eq) were dissolved by *N,N*-dimethylacetamide (15 mL). The solution was degassed by bubbling nitrogen for 10 min, followed by addition of Cu_2O (102 mg, 0.71 mmol, 0.05 equiv) under nitrogen. The solution was warmed to 70 °C and stirred for 16–36 h until TLC showed the complete consumption of 2,4-dibromobenzoic acid. The mixture was cooled to room temperature and filtered through Celite to remove the solids. H_2O was added to the mixture at 25 °C, the pH was adjusted to 4–5 by addition of 2 N HCl and the mixture were extracted with EtOAc. The combined organic layer was washed with H_2O, brine, dried over anhydrous Na_2SO_4, filtered and concentrated. Then the crude acid was directly esterified by ***General procedure a***.

General procedure d: The mixture of compound **2** (150 mg, 1equiv), $Pd(OAc)_2$ (10 mg, 0.1equiv), NaO*t*-Bu (5 mg, 0.1equiv), and pivalic acid (500 mg) was heated to 120 °C under air and stirred for 24–72 h until TLC showed the complete consumption of compounds **2** and any intermediate. The solution was cooled to rt, diluted with EtOAc, washed with 10% Na_2CO_3 aqueous solution, dried over anhydrous Na_2SO_4, filtered and evaporated under reduced pressure. The crude product was purified by silica gel column chromatography to afford the corresponding product.

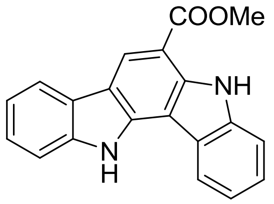

1a

125 mg; isolated yield 85%; white solid; IR (KBr) 3453, 3386, 1673, 1613, 1477, 1238, 1096, 732; ^{1}H NMR (400 MHz, DMSO-*d*$_6$) δ 12.08 (s, 1H), 11.58 (s, 1H), 8.84 (s, 1H), 8.69 (d, J = 7.6 Hz, 1H), 8.24 (d, J = 7.6 Hz, 1H), 7.84 (d, J = 8.0 Hz, 1H), 7.66 (d, J = 8.0 Hz, 1H), 7.48-7.40 (m, 2H), 7.34 (t, J = 7.6 Hz, 1H), 7.26 (t, J = 7.6 Hz, 1H), 4.03 (s, 3H). ^{13}C NMR (125 MHz, DMSO-*d*$_6$) δ 167.0, 140.2, 139.0, 138.3, 137.2, 124.8, 124.7, 123.5, 121.2, 121.0, 120.6, 120.0, 119.5, 119.4, 115.0, 112.1, 111.4, 106.7, 104.5, 51.7. HRMS (ESI) m/z calcd for $C_{20}H_{14}N_2O_2Na$ $(M+Na)^+$ 337.0953, found 337.0959.

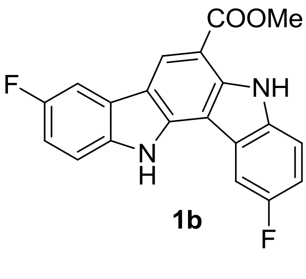

1b

127 mg; isolated yield 86%; white powder; IR (KBr) 3472, 3405, 1681, 1649, 1591, 1491, 1252, 1098, 798; ^{1}H NMR (400 MHz, DMSO-*d*$_6$) δ 12.15 (s, 1H), 11.65 (s, 1H), 8.88 (s, 1H), 8.52 (d, J = 9.2 Hz, 1H), 8.11 (d, J = 8.8 Hz, 1H), 7.82 (dd, J = 8.4, 4.4 Hz, 1H), 7.61 (dd, J = 8.4, 4.0 Hz, 1H), 7.31(t, J = 8.4 Hz, 1H), 7.24 (t, J = 8.4 Hz, 1H), 4.01 (s, 3H). ^{13}C NMR (125 MHz, DMSO-*d*$_6$) δ 166.8, 157.4 (d, J_{C-F} = 232.0 Hz), 157.2 (d, J_{C-F} = 232.0 Hz), 139.4, 138.1, 136.4, 135.5, 124.3 (d,

J_{C-F} = 9.8 Hz), 122.6, 120.7 (d, J_{C-F} = 10.8 Hz), 114.6 (d, J_{C-F} = 3.8 Hz), 113.1 (d, J_{C-F} = 9.3 Hz), 112.5 (d, J_{C-F} = 25.0 Hz), 112.2 (d, J_{C-F} = 11.9 Hz), 112.1 (d, J_{C-F} = 4.8 Hz), 106.5 (d, J_{C-F} = 3.9 Hz), 106.3 (d, J_{C-F} = 24.4 Hz), 105.6 (d, J_{C-F} = 24.3 Hz), 104.8, 51.7. HRMS (ESI) m/z calcd for $C_{20}H_{12}F_2N_2O_2Na$ $(M+Na)^+$ 373.0765, found 373.0774.

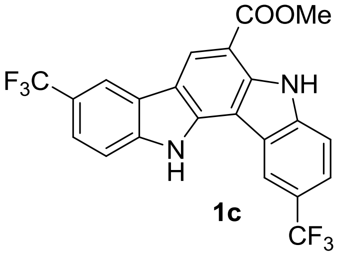

1c

123 mg; isolated yield 83%; white powder; IR (KBr) 3459, 3426, 1694, 1649, 1598, 1244, 1018, 738; ^{1}H NMR (500 MHz, DMSO-*d*$_6$) δ 12.61 (bs, 1H), 11.96 (bs, 1H), 9.09-8.98 (m, 2H), 8.69 (bs, 1H), 7.97 (bs, 1H), 7.77-7.72 (m, 3H), 4.01 (s, 3H). ^{13}C NMR (125 MHz, DMSO-*d*$_6$) δ 166.7, 142.1, 140.9, 139.5, 137.7, 125.8 (q, J_{C-F} = 268.8 Hz), 125.6 (q, J_{C-F} = 270.0 Hz), 123.3, 122.9, 121.7 (q, J_{C-F} = 3.8 Hz), 121.5 (q, J_{C-F} = 3.8 Hz), 120.9 (q, J_{C-F} = 31.3 Hz), 120.6 (q, J_{C-F} = 32.5 Hz), 120.1, 118.8 (q, J_{C-F} = 3.8 Hz), 117.5 (q, J_{C-F} = 3.8 Hz), 115.1, 112.7, 111.9, 106.7, 105.9, 51.9. HRMS (ESI) m/z calcd for $C_{22}H_{12}F_6N_2O_2Na$ $(M+Na)^+$ 473.0701, found 473.0715.

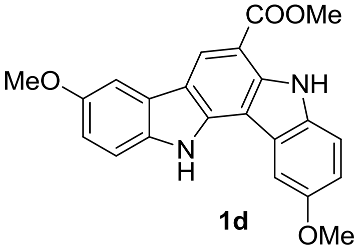

1d

91 mg; isolated yield 61%; white solid; IR(KBr) 3393, 2949, 1674, 1589, 1210, 1096, 795; ^{1}H NMR (400 MHz, DMSO-*d*$_6$) δ 11.91 (s, 1H), 11.40 (s, 1H), 8.84 (s, 1H), 8.21 (d, J = 2.0 Hz, 1H), 7.86 (d, J = 2.0 Hz, 1H), 7.74 (d, J = 9.2 Hz, 1H), 7.57 (d, J = 8.4 Hz, 1H), 7.11 (dd, J = 9.2, 2.0 Hz, 1H), 7.05 (dd, J = 9.2, 2.0 Hz, 1H), 4.03 (s, 3H), 3.98 (s, 3H), 3.90 (s, 3H). ^{13}C NMR (100 MHz, DMSO-*d*$_6$) δ 167.0, 154.1, 153.9, 138.7, 137.8, 134.6, 133.7, 124.3, 121.5, 120.9, 114.8, 113.8, 113.5, 112.7, 111.8, 106.7, 104.1, 103.9, 102.7, 56.0, 55.6, 51.6. HRMS (ESI) m/z calcd for $C_{22}H_{18}N_2O_4Na$ $(M+Na)^+$ 397.1164, found 397.1162.

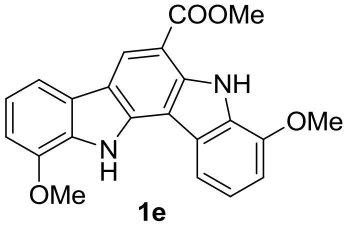

1e

86 mg; isolated yield 58%; white solid; IR (KBr) 3453, 2965, 1689, 1645, 1580, 1507, 1240, 1086, 725; ^{1}H NMR (400 MHz, DMSO-*d*$_6$) δ 12.06 (s, 1H), 10.38 (s, 1H), 8.79 (s, 1H), 8.68 (d, J = 7.6 Hz, 1H), 7.82 (d, J = 7.6 Hz, 1H), 7.27-7.18 (m, 2H), 7.06 (d, J = 8.0 Hz, 1H), 7.03 (d, J = 8.0 Hz,

1H), 4.05 (s, 3H), 4.03 (s, 3H), 4.00 (s, 3H). ^{13}C NMR (125 MHz, DMSO-*d$_6$*) δ 167.5, 146.0, 145.2, 138.1, 137.1, 129.6, 127.9, 125.0, 121.9, 121.1, 120.9, 120.4, 116.1, 115.1, 111.9, 107.61, 106.27, 105.8, 104.5, 55.6, 55.5, 51.9. HRMS (ESI) m/z calcd for $C_{22}H_{18}N_2O_4Na$ $(M+Na)^+$ 397.1164, found 397.1151.

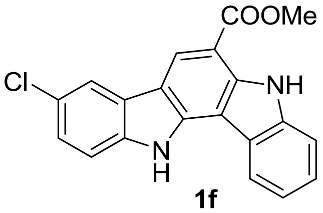

1f

93 mg; isolated yield 63%; white powder; IR (KBr) 3439, 3391, 1669, 1621, 1589, 1291, 1240, 1098, 731; ^{1}H NMR (400 MHz, DMSO-*d$_6$*) δ 12.20 (s, 1H), 11.62 (s, 1H), 8.90 (s, 1H), 8.66 (d, J = 7.6 Hz, 1H), 8.36 (s, 1H), 7.85 (d, J = 8.0 Hz, 1H), 7.64 (d, J = 8.4 Hz, 1H), 7.47 (t, J = 7.6 Hz, 1H), 7.41 (dd, J = 8.4, 1.2 Hz, 1H), 7.35 (t, J = 7.6 Hz, 1H), 4.02 (s, 3H). ^{13}C NMR (125 MHz, DMSO-*d$_6$*) δ 166.9, 139.0, 138.7, 138.5, 137.8, 125.1, 124.8, 124.4, 124.4, 121.8, 120.9, 120.4, 119.6, 119.3, 114.2, 112.7, 112.2, 106.7, 105.1, 51.7. HRMS (ESI) m/z calcd for $C_{20}H_{13}ClN_2O_2Na$ $(M+Na)^+$ 371.0563, found 371.0574.

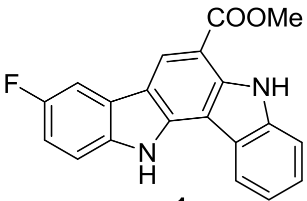

1g

117 mg; isolated yield 78%; light yellow powder; IR (KBr) 3435, 2975, 1671, 1646, 1614, 1252, 1096, 729; ^{1}H NMR (400 MHz, DMSO-*d$_6$*) δ 12.11 (s, 1H), 11.61 (s, 1H), 8.88 (s, 1H), 8.65 (d, J = 8.0 Hz, 1H), 8.12 (dd, J = 9.6, 2.4 Hz, 1H), 7.84 (d, J = 8.4 Hz, 1H), 7.64 (d, J = 4.4 Hz, 1H), 7.61 (d, J = 4.4 Hz, 1H), 7.46 (t, J = 7.2 Hz, 1H), 7.35 (t, J = 7.2 Hz, 1H), 7.24 (dt, J = 8.8, 2.4 Hz, 1H), 4.02 (s, 3H). ^{13}C NMR (500 MHz, DMSO-*d$_6$*) δ 166.9, 154.1, 141.9, 139.1, 137.9, 133.9, 125.5 (q, $J_{C\text{-}F}$ = 268.8 Hz), 123.6, 121.9, 121.2 (q, $J_{C\text{-}F}$ = 5.0 Hz), 120.7, 120.5, 117.3 (q, $J_{C\text{-}F}$ = 6.3 Hz), 114.4, 114.1, 112.9, 111.7, 106.9, 105.6, 104.0, 56.1, 51.7. HRMS (ESI) m/z calcd for $C_{20}H_{13}FN_2O_2Na$ $(M+Na)^+$ 355.0859, found 355.0853.

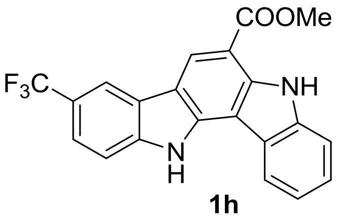

1h

124 mg; isolated yield 83%; white powder; IR (KBr) 3435, 3383, 1672, 1613, 1587, 1299, 1101, 1059, 734; ^{1}H NMR (400 MHz, DMSO-*d$_6$*) δ 12.45 (s, 1H), 11.66 (s, 1H), 9.03 (s, 1H), 8.77-8.60 (m, 2H), 7.86 (d, J = 8.0 Hz, 1H), 7.81 (d, J = 8.0 Hz, 1H), 7.71 (d, J = 8.0 Hz, 1H), 7.48 (t, J = 7.6 Hz, 1H), 7.36 (t, J = 7.2 Hz, 1H), 4.03 (s, 3H). ^{13}C NMR (100 MHz, DMSO-*d$_6$*) δ 166.9, 142.1,

139.1, 138.6, 137.9, 125.5 (q, J_{C-F} = 270.9 Hz), 124.9, 123.5, 121.9, 121.3 (q, J_{C-F} = 5.1 Hz), 121.0, 120.5, 120.3, 119.6, 117.4 (q, J_{C-F} = 3.7 Hz), 114.5, 112.2, 111.8, 106.9, 105.6, 51.8. HRMS (ESI) m/z calcd for $C_{21}H_{13}F_3N_2O_2Na$ $(M+Na)^+$ 405.0827, found 405.0831.

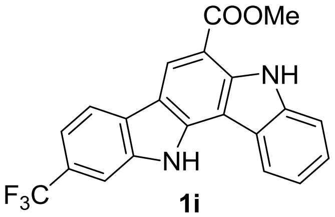

1i

110 mg; isolated yield 74%; white powder; IR (KBr) 3419, 2958, 1676, 1649, 1590, 1443, 1268, 1058, 735; ^{1}H NMR (400 MHz, DMSO-d_6) δ 12.42 (s, 1H), 11.72 (s, 1H), 8.96 (s, 1H), 8.67 (d, J = 8.0 Hz, 1H), 8.49 (d, J = 8.4 Hz, 1H), 7.92 (s, 1H), 7.89 (d, J = 8.4 Hz, 1H), 7.58 (d, J = 8.0 Hz, 1H), 7.51 (t, J = 7.6 Hz, 1H), 7.40 (t, J = 7.6 Hz, 1H), 4.06 (s, 3H); ^{13}C NMR (100 MHz, DMSO-d_6) δ 166.8, 139.3, 139.0, 138.7, 138.2, 126.7, 125.1 (q, J_{C-F} = 271.3 Hz), 125.0, 124.9, 122.0, 120.9, 120.4, 120.3, 119.7, 116.4 (d, J_{C-F} = 4.2 Hz), 114.0, 112.3, 108.0 (t, J_{C-F} = 5.0 Hz), 106.7, 105.7, 51.8. HRMS (ESI) m/z calcd for $C_{21}H_{13}F_3N_2O_2Na$ $(M+Na)^+$ 405.0827, found 405.0834

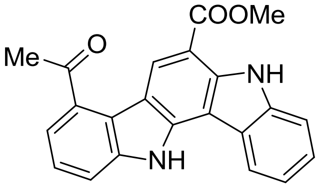

1j

91 mg; isolated yield 61%; yellow solid; IR (KBr) 3347, 1685, 1613, 1585, 1544, 1315, 1150, 1087, 772; ^{1}H NMR (500 MHz, DMSO-d_6) δ 12.33 (s, 1H), 11.63 (s, 1H), 9.63 (s, 1H), 8.71 (d, J = 8.0 Hz, 1H), 7.96 (d, J = 7.5 Hz, 1H), 7.93 (d, J = 7.5 Hz, 1H), 7.86 (d, J = 8.0 Hz, 1H), 7.56 (t, J = 8.0 Hz, 1H), 7.47 (t, J = 8.0 Hz, 1H), 7.36 (t, J = 7.5 Hz, 1H), 4.04 (s, 3H), 2.82 (s, 3H); ^{13}C NMR (125 MHz, DMSO-d_6) δ 201.3, 167.6, 141.8, 139.4, 138.9, 138.7, 132.6, 127.5, 125.1, 124.4, 123.9, 121.3, 121.2, 121.1, 120.0, 116.6, 114.6, 112.7, 106.5, 105.1, 52.2, 29.5. HRMS (ESI) m/z calcd for $C_{22}H_{16}N_2O_3Na$ $(M+Na)^+$ 379.1059, found 379.1057.

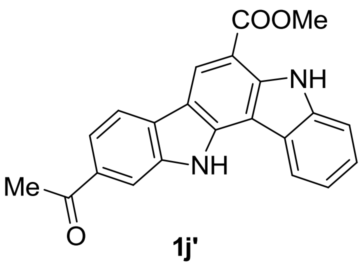

1j'

19mg; isolated yield 13%; yellow solid; IR (KBr) 3344, 1681, 1616, 1584, 1540, 1314, 1149, 1086, 696; ^{1}H NMR (400 MHz, DMSO-d_6) δ 12.33 (s, 1H), 11.70 (s, 1H), 8.93 (s, 1H), 8.68 (d, J = 7.6 Hz, 1H), 8.38 (d, J = 8.0 Hz, 1H), 8.23 (s, 1H), 7.91 (d, J = 8.0 Hz, 1H), 7.87 (d, J = 8.0 Hz, 1H), 7.49 (t, J = 7.6 Hz, 1H), 7.39 (t, J = 7.6 Hz, 1H), 4.04 (s, 3H), 2.72 (s, 3H). ^{13}C NMR (100

MHz, DMSO-d_6) δ 198.0, 167.3, 140.2, 139.5, 139.2, 139.1, 133.8, 128.0, 125.4, 122.6, 121.4, 120.9, 120.2, 119.8, 114.7, 112.8, 111.8, 107.1, 106.0, 52.3, 27.4. HRMS (ESI) m/z calcd for $C_{22}H_{16}N_2O_3Na$ $(M+Na)^+$ 379.1059, found 379.1048.

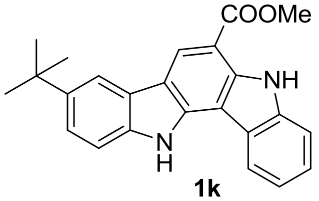

1k

98 mg; isolated yield 66%; white powder; IR (KBr) 3442, 2925, 2854, 1672, 1590, 1266, 1239, 1098, 735; ^{1}H NMR (400 MHz, DMSO-d_6) δ 11.96 (s, 1H), 11.55 (s, 1H), 8.89 (s, 1H), 8.67 (d, J = 8.0 Hz, 1H), 8.27 (s, 1H), 7.84 (d, J = 8.0 Hz, 1H), 7.58 (d, J = 8.4 Hz, 1H), 7.49 (dd, J = 8.4, 1.6 Hz, 1H), 7.46 (t, 7.6 Hz, 1H), 7.34 (t, J = 7.6 Hz, 1H), 4.04 (s, 3H), 1.44 (s, 9H). ^{13}C NMR (125 MHz, DMSO-d_6) δ 167.1, 142.6, 138.9, 138.3, 137.6, 124.6, 123.3, 122.4, 121.2, 121.0, 120.6, 119.4, 115.8, 115.3, 112.0, 110.79, 106.6, 104.2, 51.6, 34.5, 31.9. HRMS (ESI) m/z calcd for $C_{24}H_{22}N_2O_2Na$ $(M+Na)^+$ 393.1579, found 393.1571.

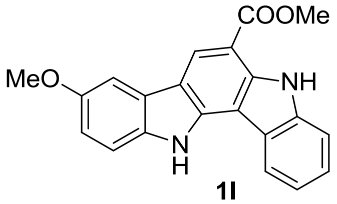

1l

118 mg; isolated yield 73%; white powder; IR (KBr) 3444, 3378, 2926, 1685, 1589, 1273, 1049, 731; ^{1}H NMR (400 MHz, DMSO-d_6) δ 11.89 (s, 1H), 11.53 (s, 1H), 8.84 (s, 1H), 8.63 (d, J = 7.6 Hz, 1H), 7.85 (d, J = 2.4 Hz, 1H), 7.82 (d, J = 8.0 Hz, 1H), 7.53 (d, J = 8.4 Hz, 1H), 7.44 (t, J = 7.6 Hz, 1H), 7.32 (t, J = 7.6 Hz, 1H), 7.02 (dd, J = 8.4, 2.0 Hz, 1H), 4.02 (s, 3H), 3.88 (s, 3H). ^{13}C NMR (100 MHz, DMSO-d_6) δ 167.0, 154.1, 149.6, 138.9, 138.2, 137.7, 134.7, 124.6, 124.17, 121.5, 120.9, 120.6, 119.4, 115.2, 113.6, 112.0, 106.6, 104.1, 102.7, 55.3, 51.4. HRMS (ESI) m/z calcd for $C_{21}H_{16}N_2O_3Na$ $(M+Na)^+$ 367.1059, found 367.1055.

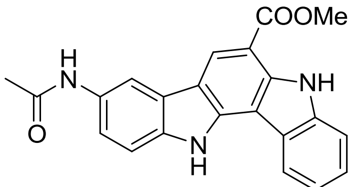

1m

60 mg; isolated yield 40%; Red solid; IR (KBr) 3406, 3300, 2924, 1672, 1488, 1373, 1244, 1095, 736; ^{1}H NMR (500 MHz, DMSO-d_6) δ 12.01 (s, 1H), 11.57 (s, 1H), 10.00 (s, 1H), 8.70 (s, 1H), 8.66 (d, J = 8.0 Hz, 1H), 8.51 (d, J = 1.5 Hz, 1H), 7.84 (d, J = 8.0 Hz, 1H), 7.59 (d, J = 9.0 Hz, 1H), 7.49 (dd, J = 8.5, 2.0 Hz, 1H), 7.46 (t, J = 8.0 Hz, 1H), 7.34 (t, J = 8.0 Hz, 1H), 4.03 (s, 3H), 2.11 (s, 3H). ^{13}C NMR (125 MHz, DMSO-d_6) δ 167.9, 166.9, 139.0, 138.3, 137.6, 136.5, 132.6, 124.7, 123.3, 121.0, 120.8, 120.5, 119.5, 117.7, 115.0, 112.1, 111.3, 110.0, 106.7, 104.4, 51.7, 24.0. HRMS (ESI) m/z calcd for $C_{22}H_{17}N_3O_3Na$ $(M+Na)^+$ 394.1168, found 394.1170.

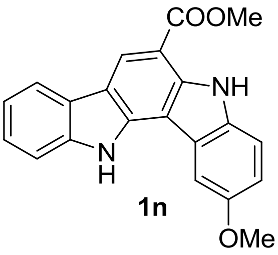

1n

105 mg; isolated yield 71%; white solid; IR (KBr) 3444, 3378, 2926, 1685, 1589, 1489, 1273, 1095, 788; ^{1}H NMR (500 MHz, DMSO-d_6) δ 12.08 (s, 1H), 11.42 (s, 1H), 8.81 (s, 1H), 8.24-8.22 (m, 2H), 7.73 (d, J = 8.5 Hz, 1H), 7.67 (d, J = 8.0 Hz, 1H), 7.43 (t, J = 6.0 Hz, 1H), 7.26 (d, J = 7.5 Hz, 1H), 7.10 (dd, J = 7.2, 2.0 Hz, 1H), 4.02 (s, 3H), 3.97 (s, 3H). ^{13}C NMR (125 MHz, DMSO-d_6) δ 167.0, 153.9, 140.1, 138.8, 137.3, 133.8, 124.7, 123.6, 121.1, 120.9, 120.0, 119.5, 114.6, 114.0, 112.7, 111.3, 106.8, 104.5, 104.0, 56.1, 51.7. HRMS (ESI) m/z calcd for $C_{21}H_{16}N_2O_3Na$ $(M+Na)^+$ 367.1059, found 367.1055.

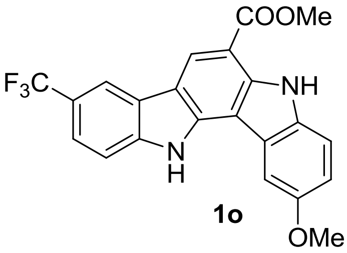

1o

82 mg; isolated yield 55%; white solid; IR (KBr) 3444, 3385, 2926, 1673, 1588, 1293, 1056, 786; ^{1}H NMR (400 MHz, DMSO-d_6) δ 12.44 (s, 1H), 11.51 (s, 1H), 9.02 (s, 1H), 8.72 (s, 1H), 8.23 (d, J = 2.0 Hz, 1H), 7.82 (d, J = 8.4 Hz, 1H), 7.73 (t, J = 8.8 Hz, 2H), 7.12 (dd, J = 8.8, 2.4 Hz, 1H), 4.02 (s, 3H), 3.97 (s, 3H). ^{13}C NMR (125 MHz, DMSO-d_6) δ 166.9, 154.1, 142.0, 139.1, 138.0, 133.9, 125.5 (q, J_{C-F} = 268.8 Hz), 123.6, 121.9, 121.2 (q, J_{C-F} = 5.0 Hz), 120.7, 120.5, 117.3(q, J_{C-F} = 6.3 Hz), 114.2, 114.1, 112.9, 111.7, 107.0, 105.6, 104.0, 56.1, 51.7. HRMS (ESI) m/z calcd for $C_{22}H_{15}F_3N_2O_3Na$ $(M+Na)^+$ 435.0932, found 435.0945.

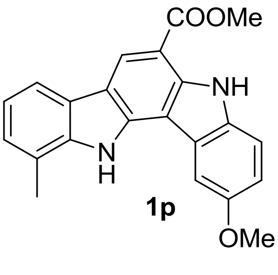

1p

74 mg; isolated yield 50%; white solid; IR (KBr) 3439, 2924, 2854, 1681, 1633, 1587, 1489, 1220, 1082, 784; ^{1}H NMR (400 MHz, $CDCl_3$) δ 10.06 (s, 1H), 8.66 (s, 1H), 8.41 (s, 1H), 7.91 (d, J = 6.4 Hz, 1H), 7.53 (s, 1H), 7.45 (d, J = 7.2 Hz, 1H), 7.23-7.22 (m, 2H), 7.11 (d, J = 8.8 Hz, 1H), 4.04 (s, 3H), 3.98 (s, 3H), 2.61 (s, 3H). ^{13}C NMR (100 MHz, $CDCl_3$) δ 168.2, 154.0, 140.3, 138.7, 137.5, 133.5, 125.6, 123.7, 121.6, 121.3, 120.8, 120.1, 117.0, 116.1, 112.6, 111.5, 106.7, 105.00, 104.9, 56.4, 51.7, 16.7. HRMS (ESI) m/z calcd for $C_{22}H_{18}N_2O_3Na$ $(M+Na)^+$ 381.1215, found 381.1224.

3. NMR spectra for compounds 3, 4a, 4b, 2a–p, 5, 1a–p

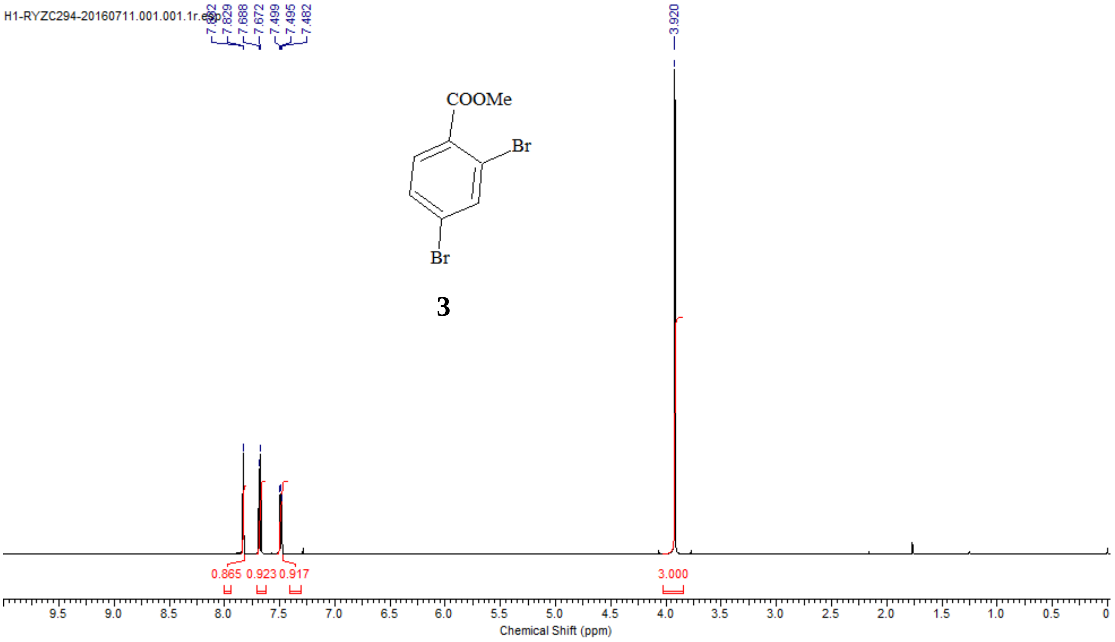

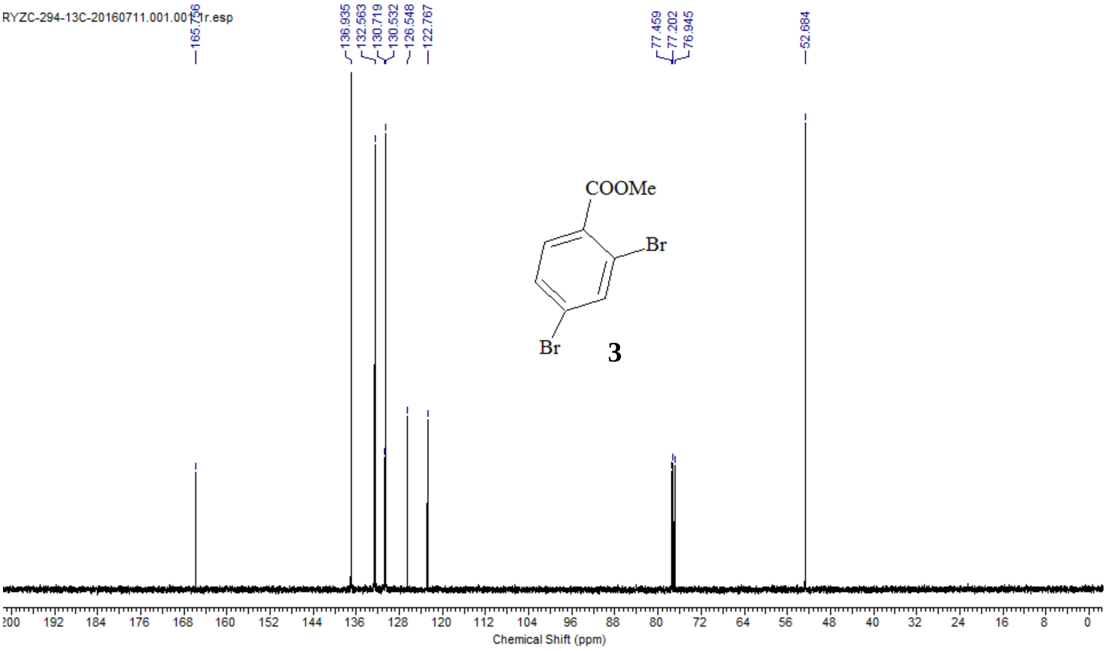

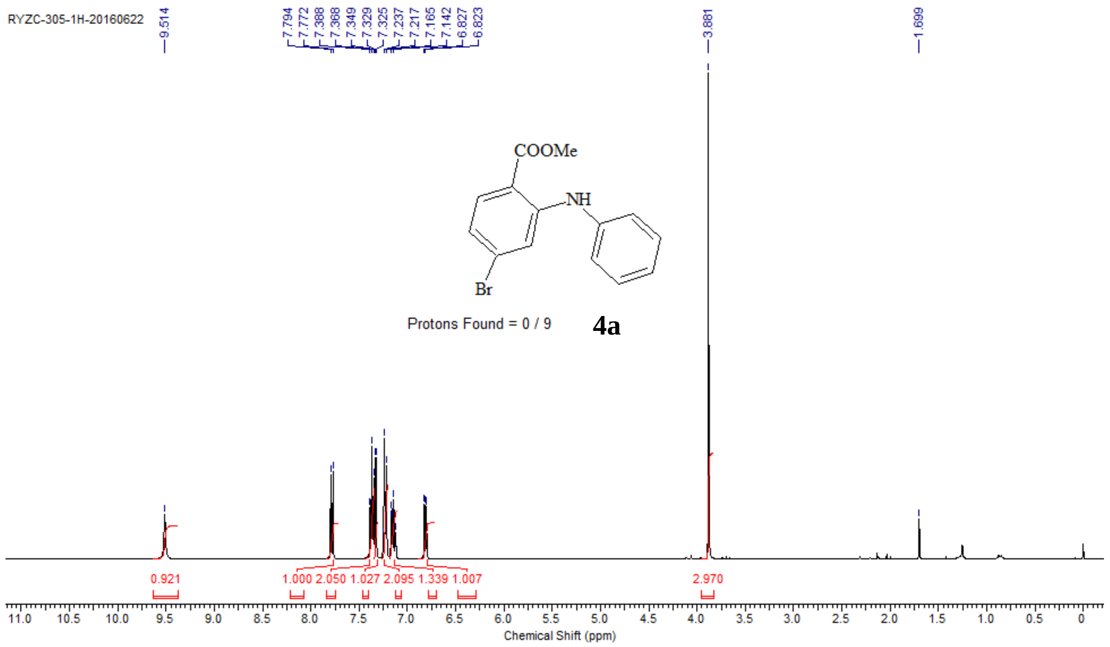
RYZC-305-1H-20160622
9.514
7.794
7.772
7.388
7.368
7.349
7.329
7.325
7.237
7.217
7.165
7.142
6.827
6.823
3.881
1.699
COOMe
NH
Br
Protons Found = 0 / 9
4a
0.921
1.000 2.050 1.027 2.095 1.339 1.007
2.970
Chemical Shift (ppm)

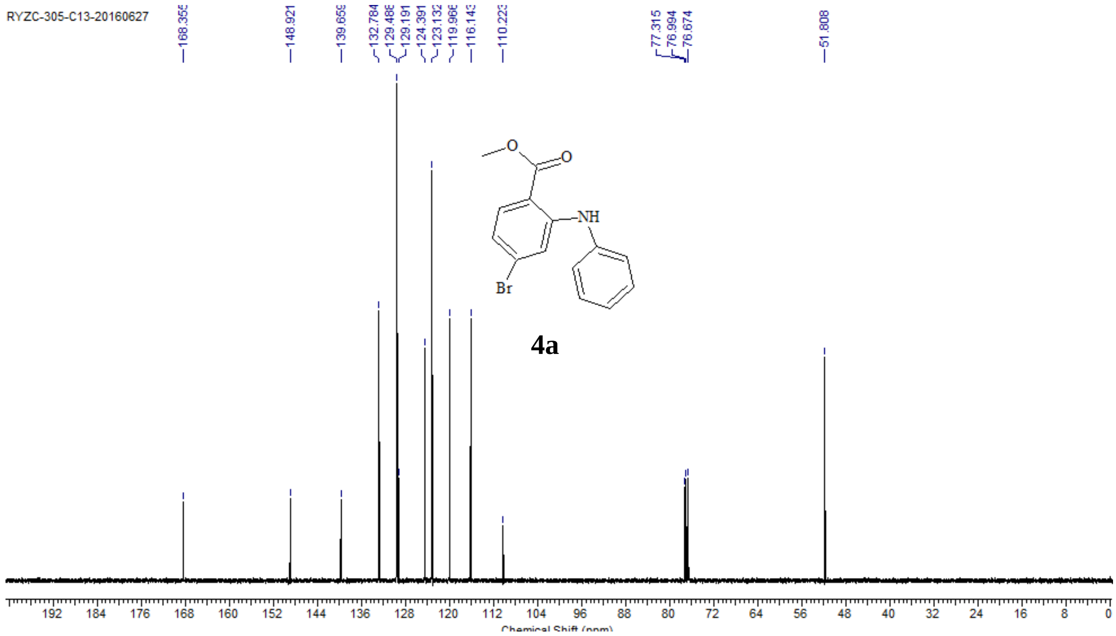
RYZC-305-C13-20160627
168.355
148.921
139.659
132.784
129.488
129.191
124.391
123.132
119.966
116.143
110.223
77.315
76.994
76.674
51.808
O
O
NH
Br
4a

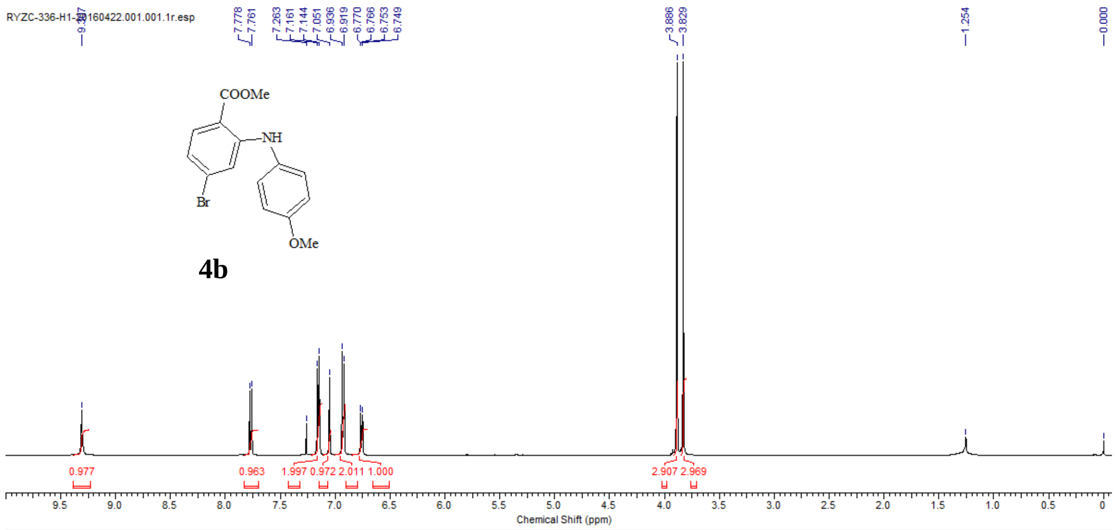
RYZC-336-H1-20160422.001.001.1r.esp
9.287
7.778
7.761
7.263
7.161
7.144
7.051
6.936
6.919
6.770
6.766
6.753
6.749
3.886
3.829
1.254
0.000
COOMe
NH
Br
OMe
4b
0.977
0.963
1.997
0.972
2.011
1.000
2.907
2.969
Chemical Shift (ppm)

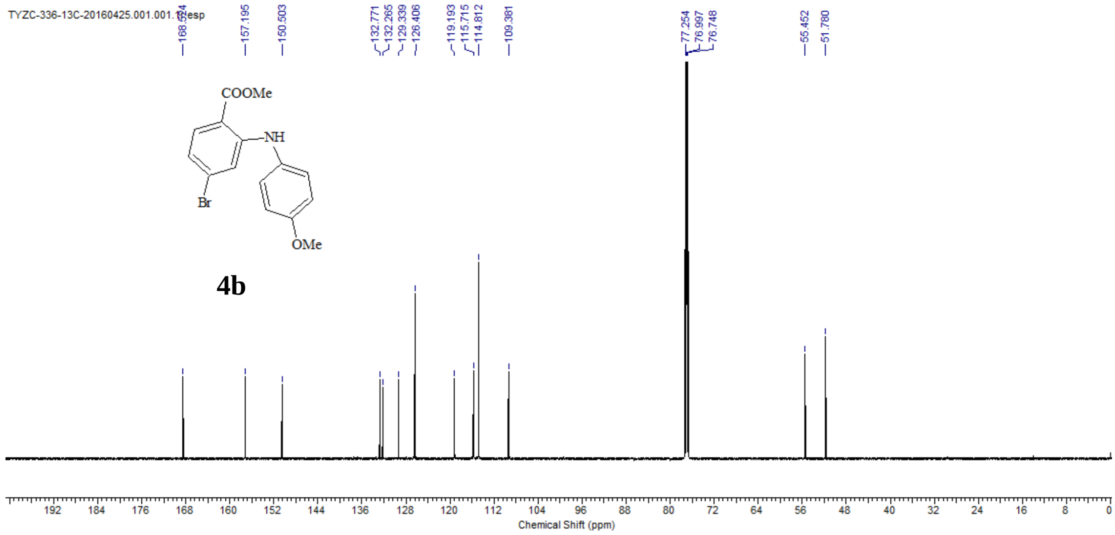
TYZC-336-13C-20160425.001.001.1r.esp
168.524
157.195
150.503
132.771
132.265
129.339
126.406
119.193
115.715
114.812
109.381
77.254
76.997
76.748
55.452
51.780
COOMe
NH
Br
OMe
4b
Chemical Shift (ppm)

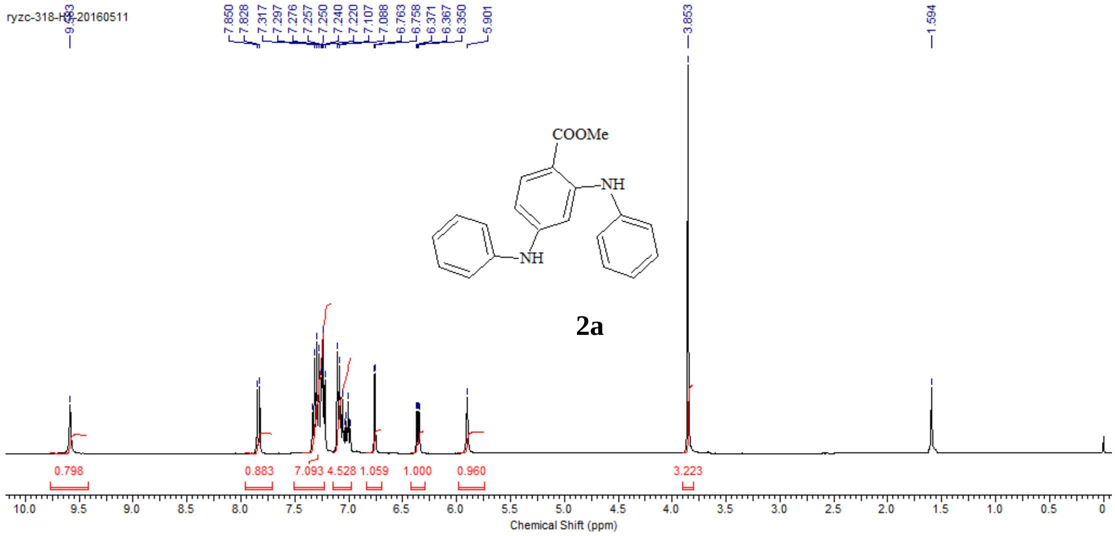
ryzc-318-H1-20160511
9.593
7.850
7.828
7.317
7.297
7.276
7.257
7.250
7.240
7.220
7.107
7.088
6.763
6.758
6.371
6.367
6.350
5.901
3.853
1.594
COOMe
NH
NH
2a
0.798
0.883
7.093
4.528
1.059
1.000
0.960
3.223
Chemical Shift (ppm)

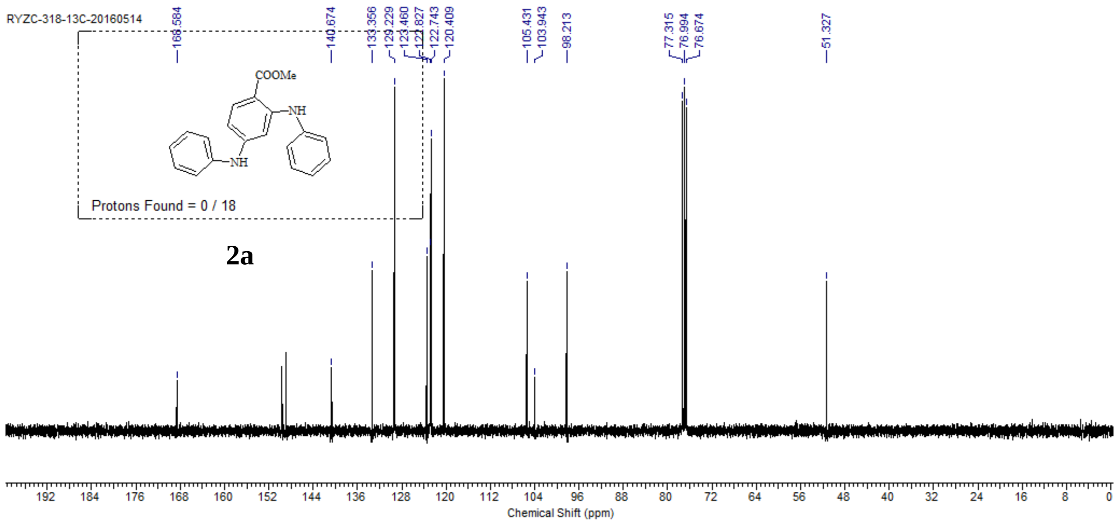
RYZC-318-13C-20160514
168.584
140.674
133.356
129.229
123.460
121.827
122.743
120.409
105.431
103.943
98.213
77.315
76.994
76.674
51.327
COOMe
NH
NH
Protons Found = 0 / 18
2a
Chemical Shift (ppm)

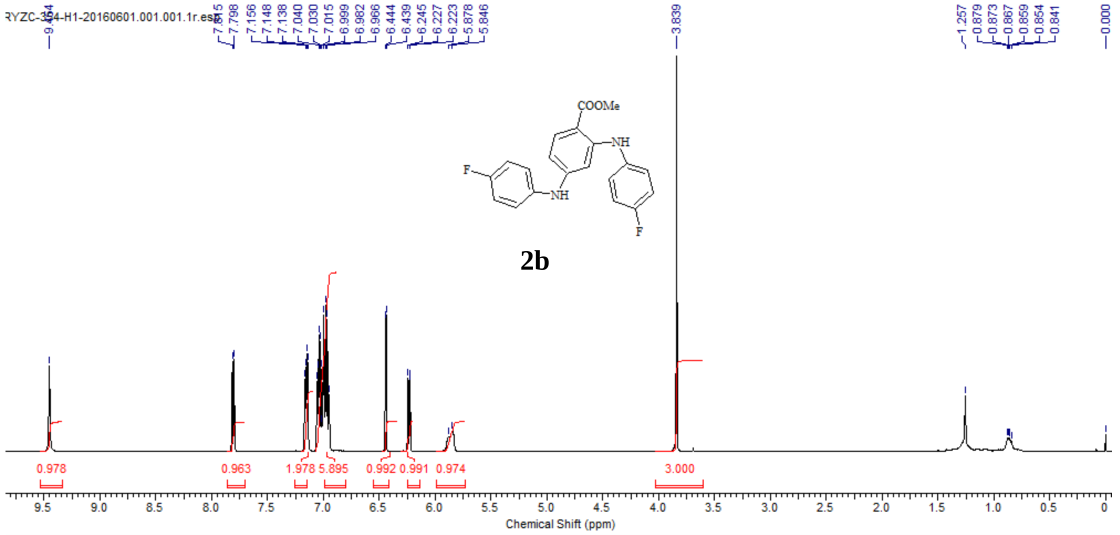
7.815
7.798
7.156
7.148
7.138
7.040
7.030
7.015
6.999
6.982
6.966
6.444
6.439
6.245
6.227
6.223
5.878
5.846
3.839
1.257
0.879
0.873
0.867
0.859
0.854
0.841
0.000
COOMe
NH
F
NH
F
2b
0.978
0.963
1.978
5.895
0.992
0.991
0.974
3.000
Chemical Shift (ppm)

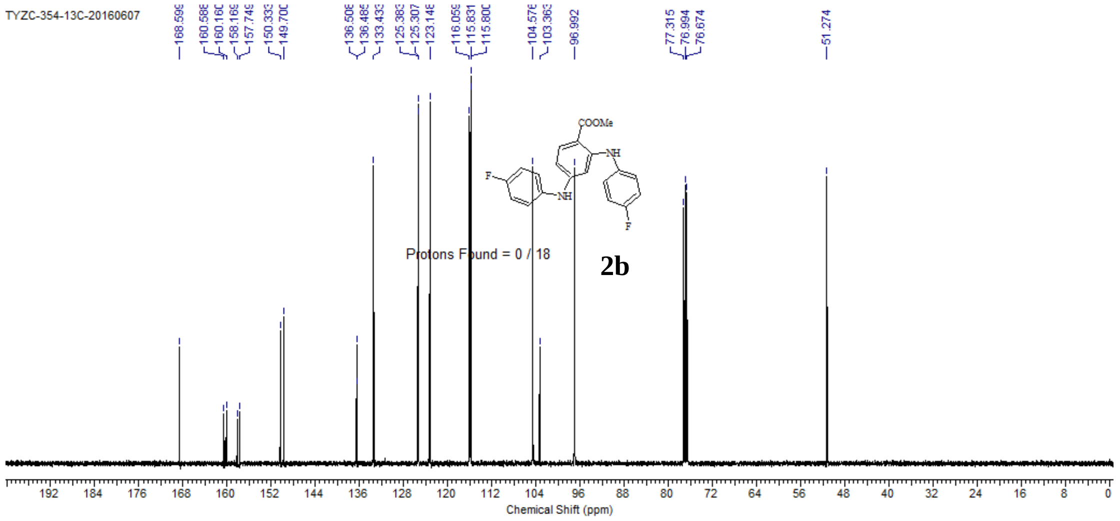
TYZC-354-13C-20160607
168.599
160.588
160.160
158.169
157.749
150.332
149.700
136.508
136.485
133.433
125.383
125.307
123.148
116.059
115.831
115.800
104.576
103.363
96.992
77.315
76.994
76.674
51.274
COOMe
NH
F
NH
F
Protons Found = 0 / 18
2b
Chemical Shift (ppm)

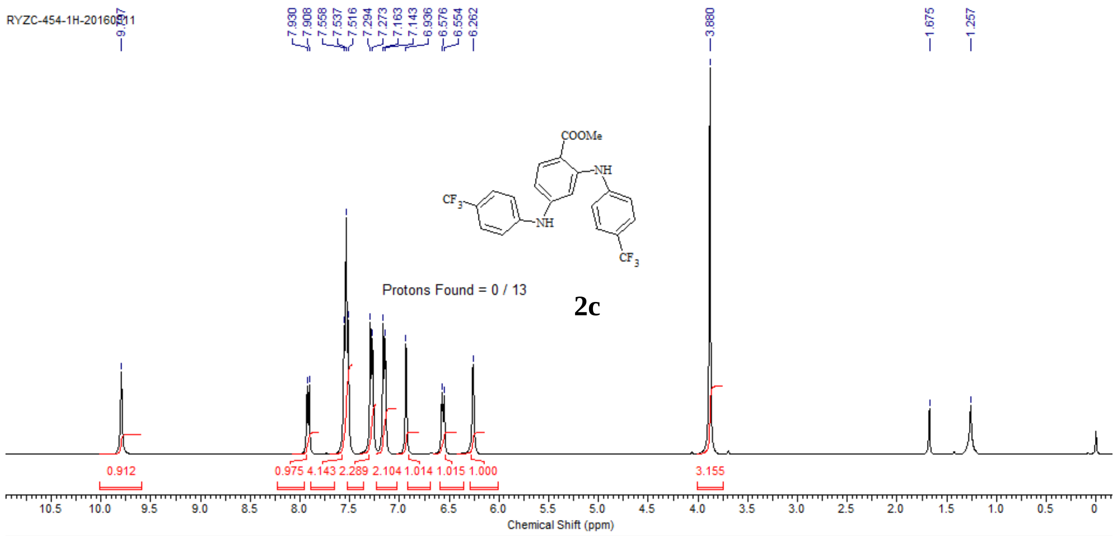
RYZC-454-1H-20160511
9.817
7.930 7.908 7.558 7.537 7.516 7.294 7.273 7.163 7.143 6.936 6.576 6.554 6.262
3.880
1.675
1.257
COOMe
NH
CF3
NH
CF3
Protons Found = 0 / 13
2c
0.912
0.975 4.143 2.289 2.104 1.014 1.015 1.000
3.155
Chemical Shift (ppm)

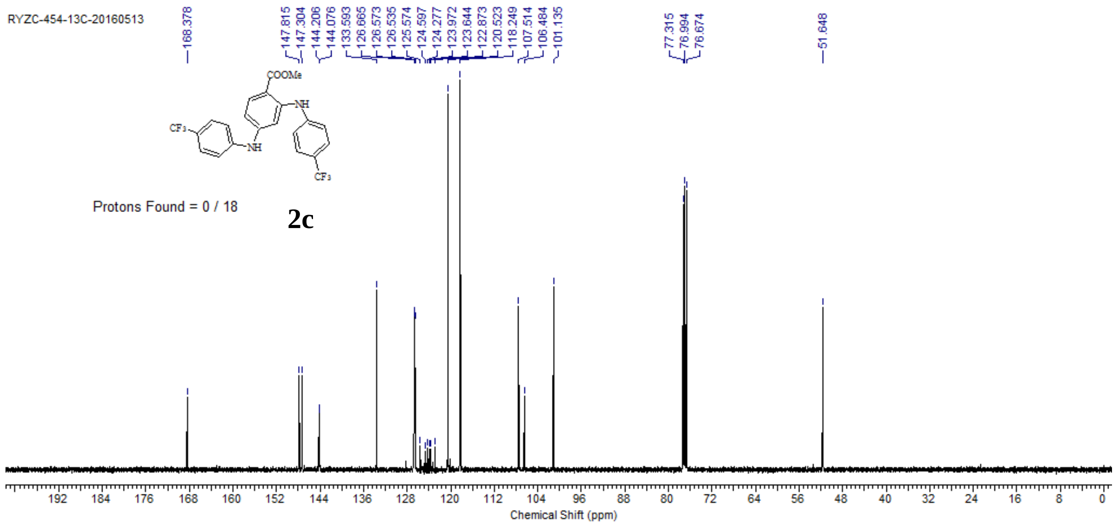
RYZC-454-13C-20160513
168.378
147.815 147.304 144.206 144.076 133.593 126.665 126.573 126.535 125.574 124.597 124.277 123.972 123.644 122.873 120.523 118.249 107.514 106.484 101.135
77.315 76.994 76.674
51.648
COOMe
NH
CF3
NH
CF3
Protons Found = 0 / 18
2c
Chemical Shift (ppm)

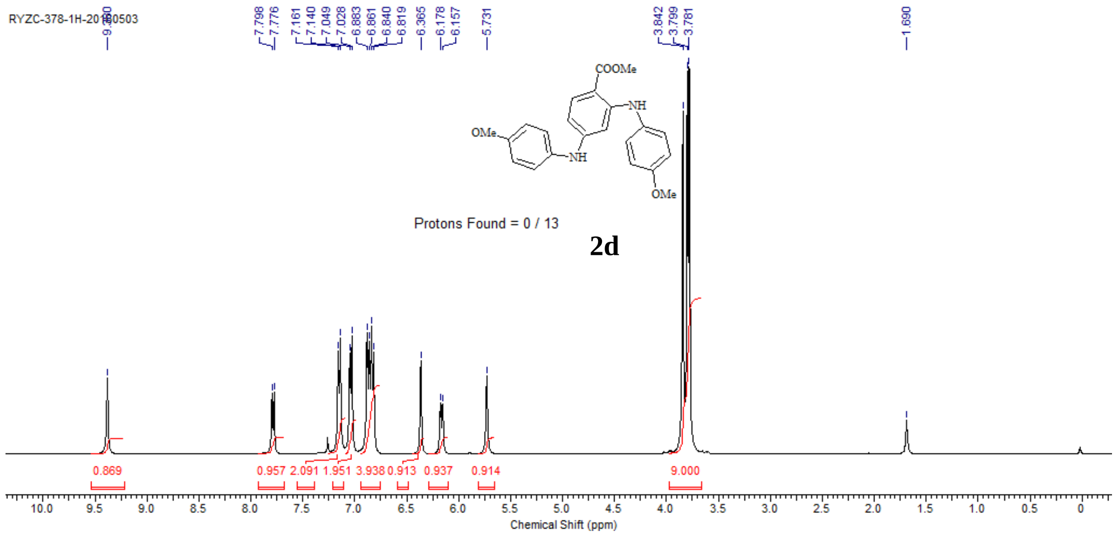
RYZC-378-1H-20160503
9.360
7.798 7.776 7.161 7.140 7.049 7.028 6.883 6.861 6.840 6.819 6.365 6.178 6.157 5.731
3.842 3.799 3.781
1.690
COOMe
NH
OMe
NH
OMe
Protons Found = 0 / 13
2d
0.869
0.957 2.091 1.951 3.938 0.913 0.937 0.914
9.000
Chemical Shift (ppm)

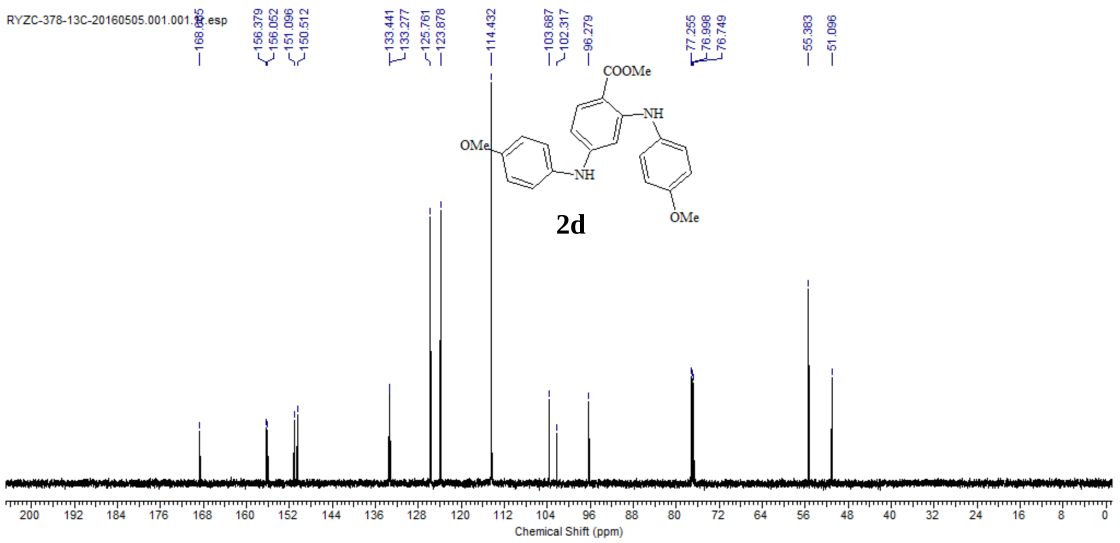
RYZC-378-13C-20160505.001.001.1r.esp
COOMe
NH
OMe
NH
OMe
2d
Chemical Shift (ppm)

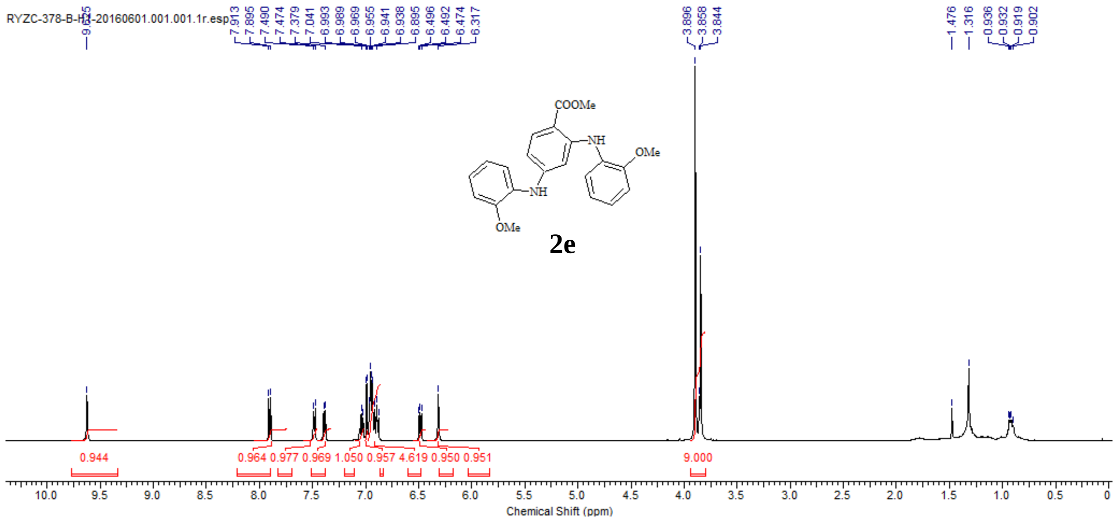
COOMe
NH
OMe
NH
OMe
2e
Chemical Shift (ppm)

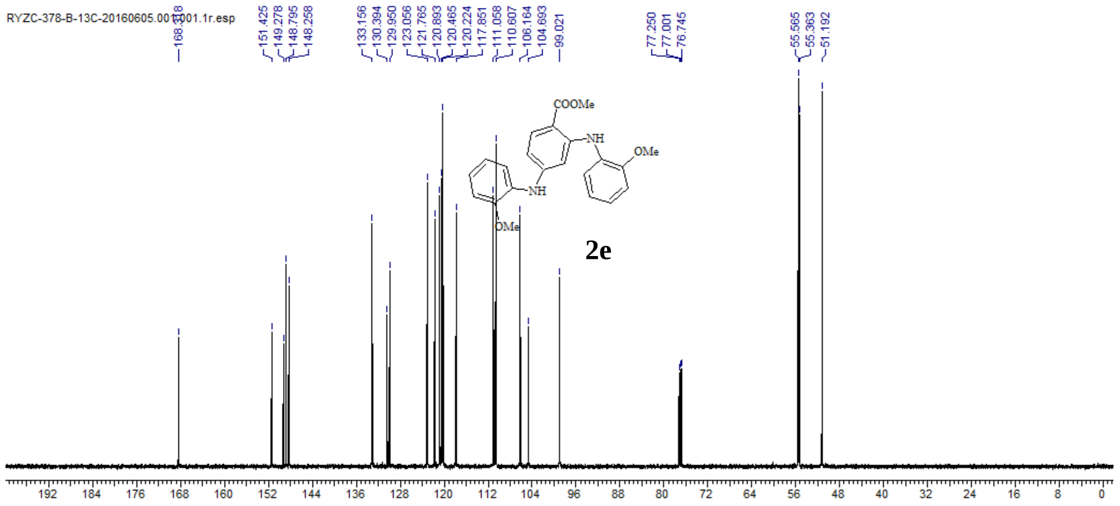
RYZC-378-B-13C-20160605.001.001.1r.esp
COOMe
NH
OMe
NH
OMe
2e

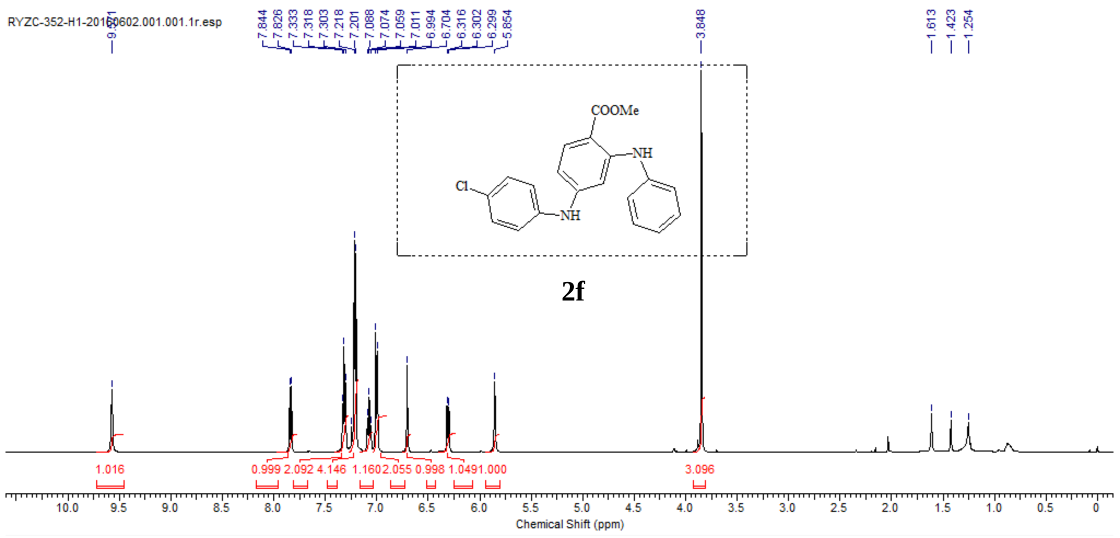
RYZC-352-H1-20160602.001.001.1r.esp
COOMe
NH
Cl
NH
2f
Chemical Shift (ppm)

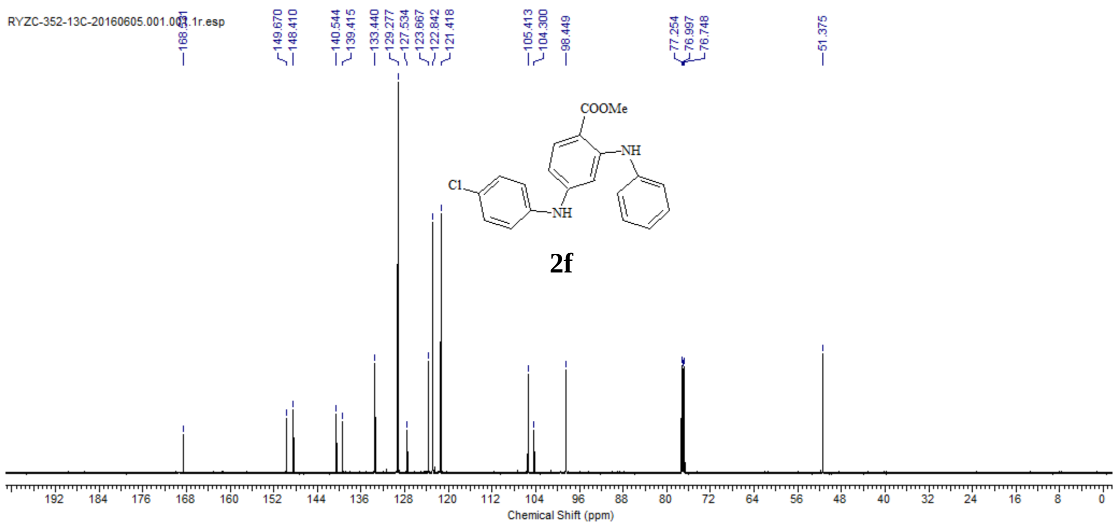
RYZC-352-13C-20160605.001.001.1r.esp
COOMe
NH
Cl
NH
2f
Chemical Shift (ppm)

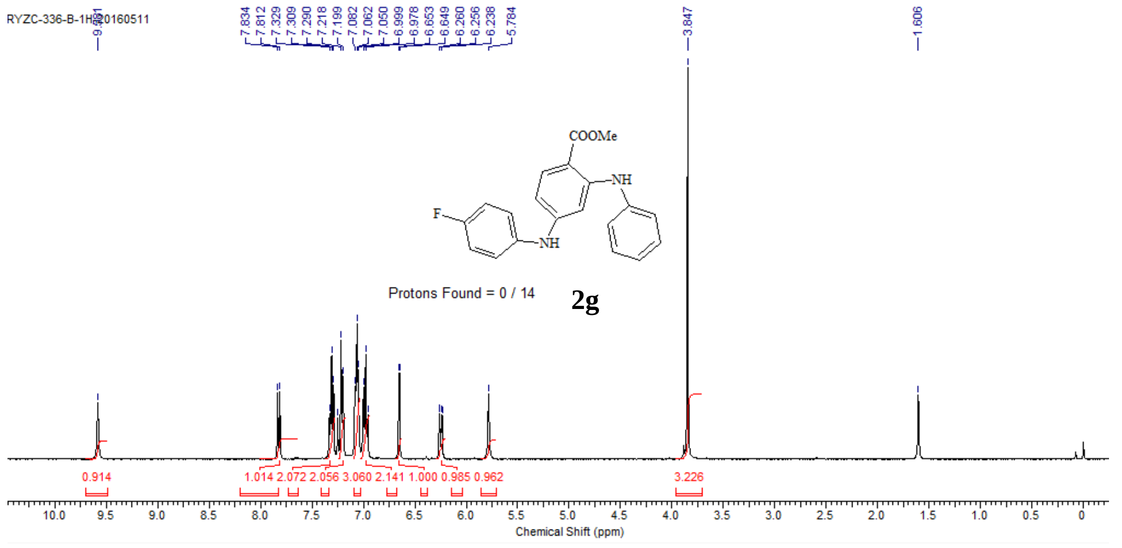
RYZC-336-B-1H-20160511
COOMe
NH
F
NH
Protons Found = 0 / 14
2g
Chemical Shift (ppm)

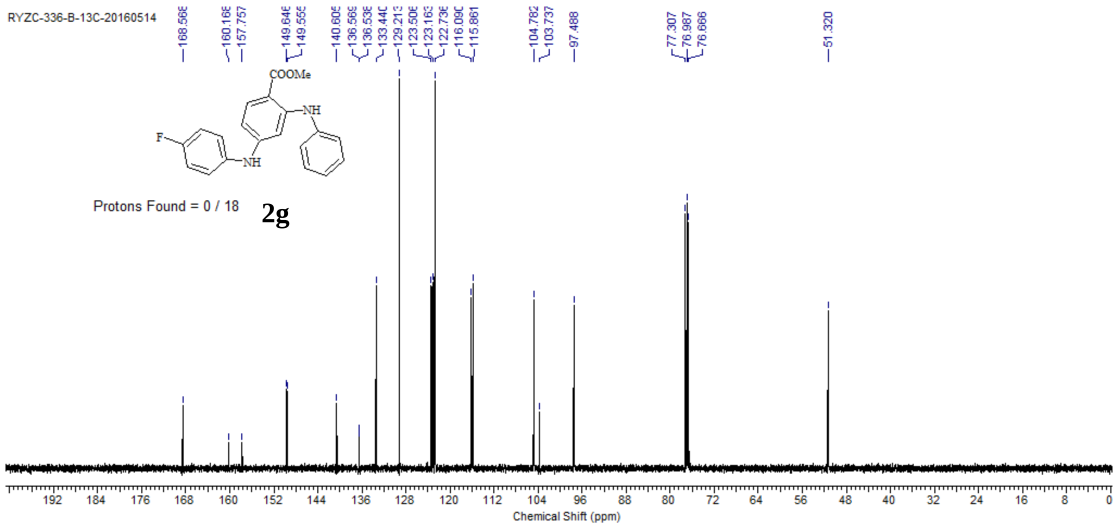
RYZC-336-B-13C-20160514
168.568
157.757
149.559
140.605
136.569
136.536
133.440
129.213
123.506
123.163
122.736
116.090
115.861
104.782
103.737
97.488
77.307
76.987
76.666
51.320
COOMe
NH
F
NH
Protons Found = 0 / 18
Chemical Shift (ppm)

2g

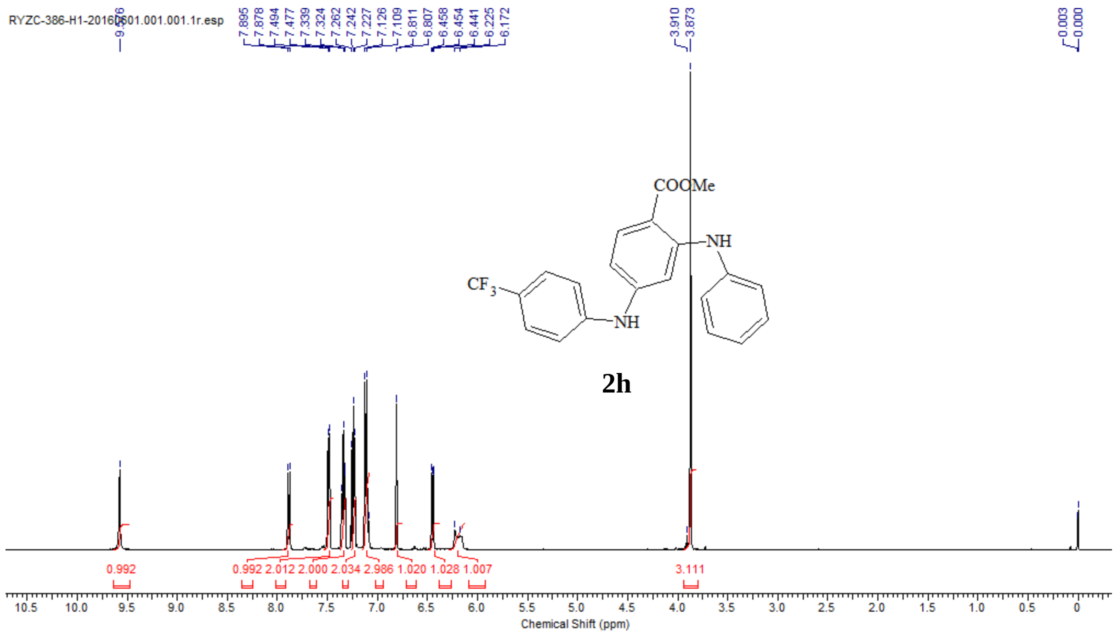
RYZC-386-H1-20160601.001.001.1r.esp
7.895
7.878
7.494
7.477
7.339
7.324
7.262
7.242
7.227
7.126
7.109
6.811
6.807
6.458
6.454
6.441
6.225
6.172
3.910
3.873
0.003
0.000
COOMe
NH
CF_3
NH
0.992
0.992 2.012 2.000 2.034 2.986 1.020 1.028 1.007
3.111
Chemical Shift (ppm)

2h

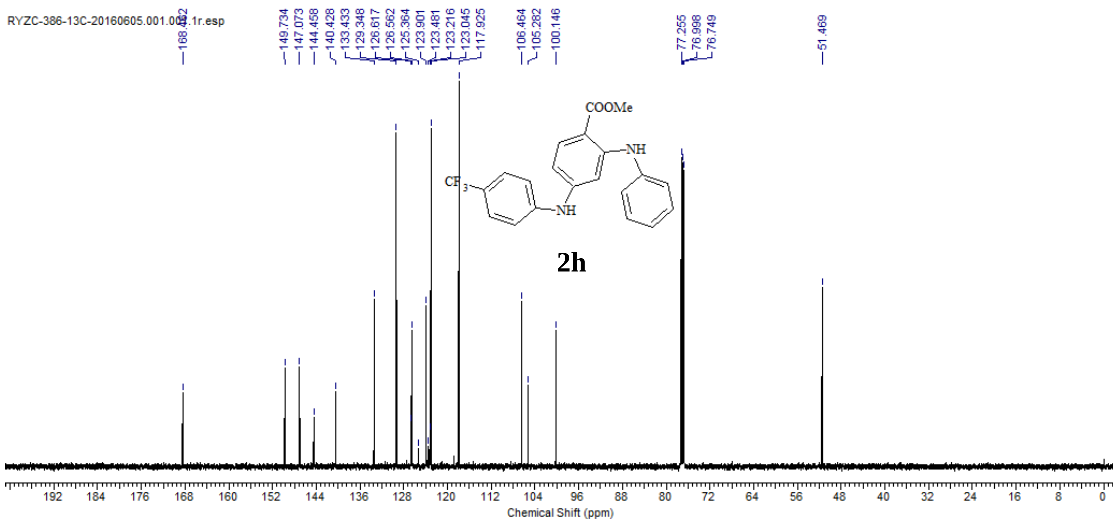
RYZC-386-13C-20160605.001.001.1r.esp
149.734
147.073
144.458
140.428
133.433
129.348
126.617
126.562
125.364
123.901
123.481
123.216
123.045
117.925
106.464
105.282
100.146
77.255
76.998
76.749
51.469
COOMe
NH
CF_3
NH
Chemical Shift (ppm)

2h

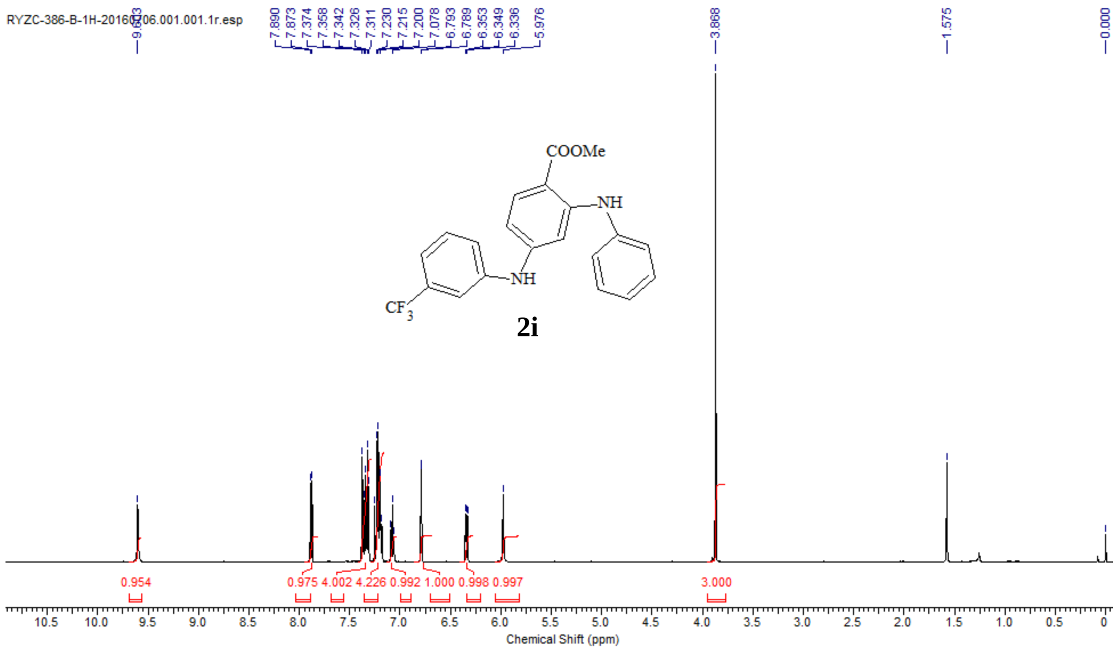
RYZC-386-B-1H-20160706.001.001.1r.esp
COOMe
NH
NH
CF3
2i
Chemical Shift (ppm)

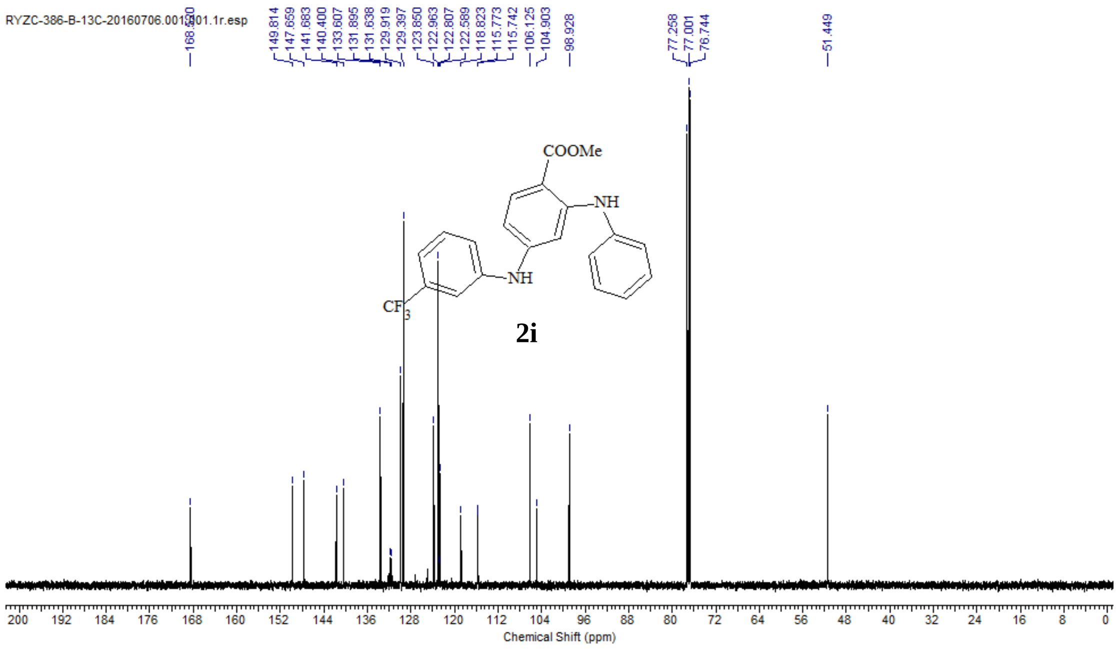
RYZC-386-B-13C-20160706.001.001.1r.esp
COOMe
NH
NH
CF3
2i
Chemical Shift (ppm)

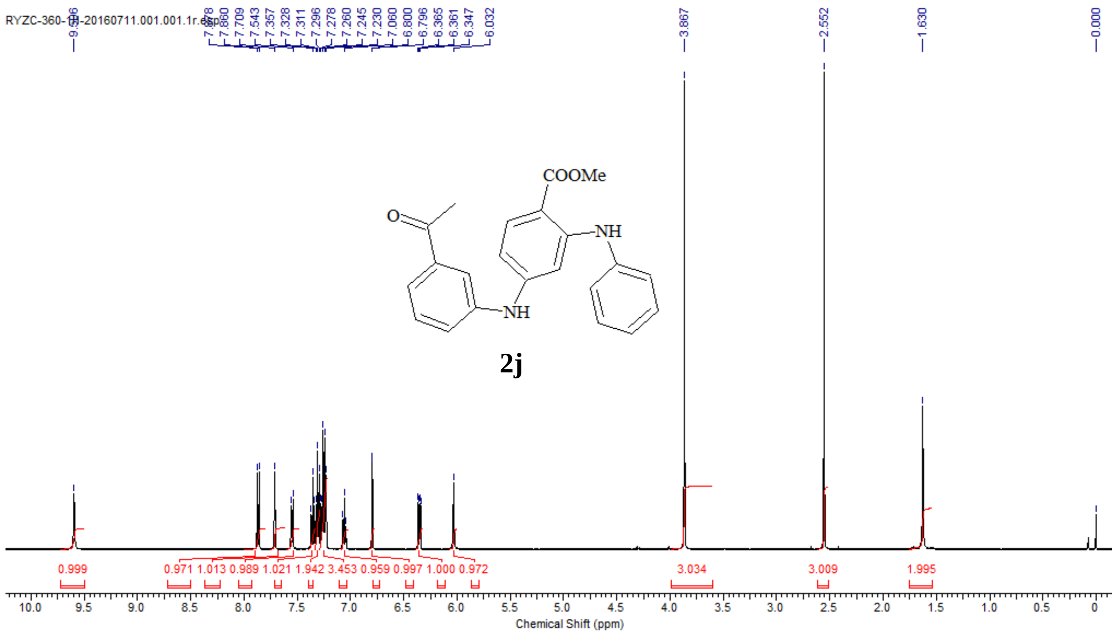
RYZC-360-1H-20160711.001.001.1r.esp
COOMe
NH
O
NH
2j
Chemical Shift (ppm)

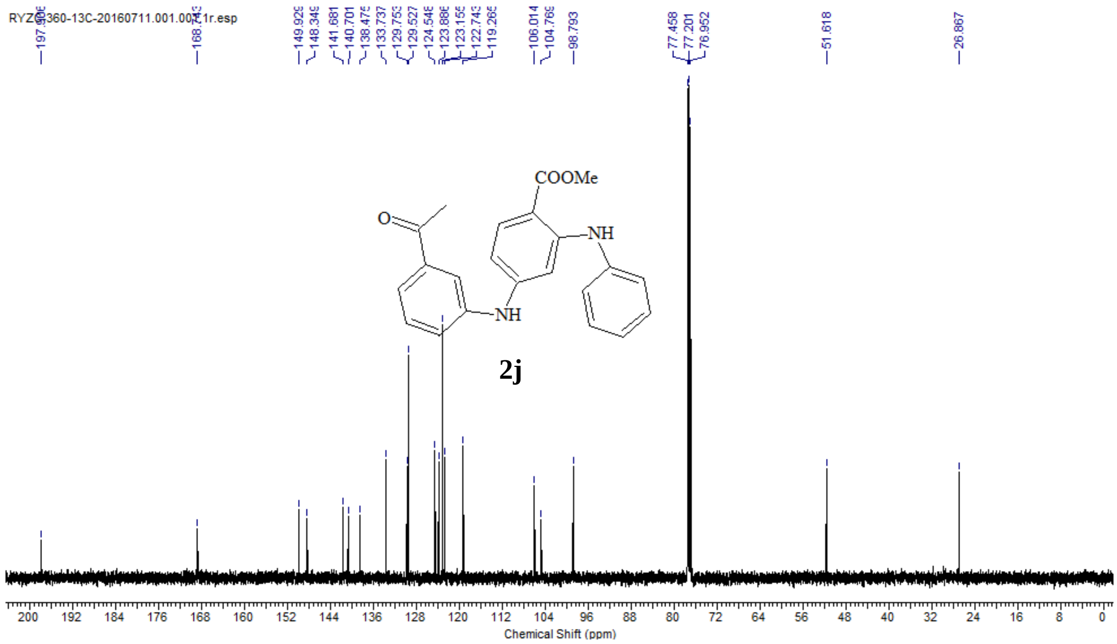
RYZC-360-13C-20160711.001.001.1r.esp
COOMe
NH
O
NH
2j
Chemical Shift (ppm)

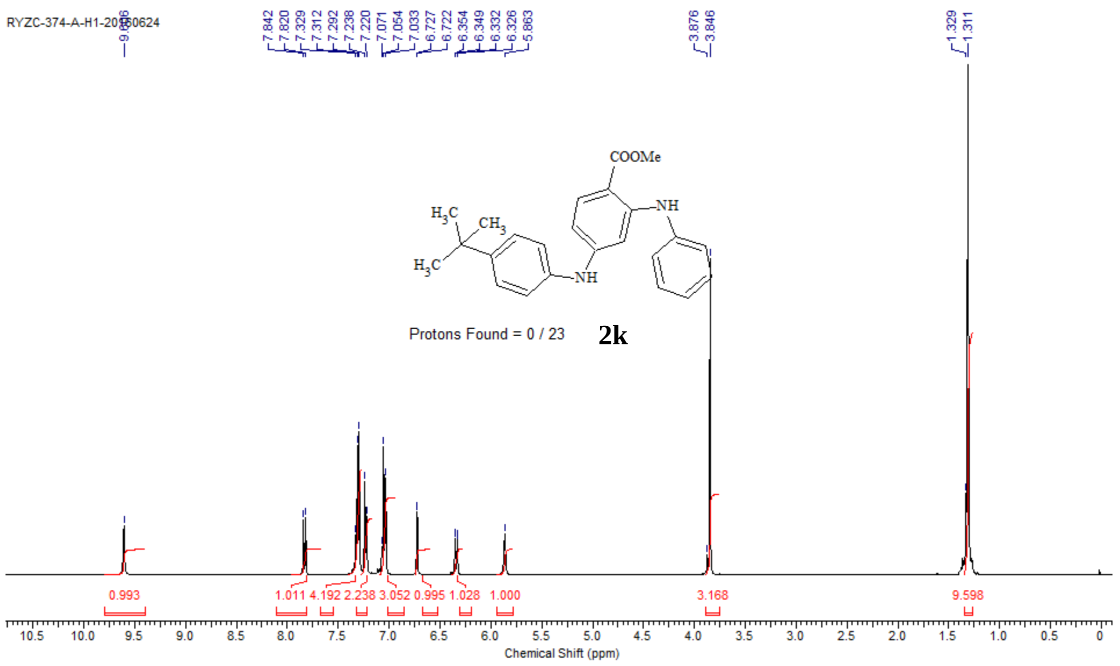
RYZC-374-A-H1-20160624
9.636
7.842
7.820
7.329
7.312
7.292
7.238
7.220
7.071
7.054
7.033
6.727
6.722
6.354
6.349
6.332
6.326
5.863
3.876
3.846
1.329
1.311
COOMe
NH
H3C
CH3
H3C
NH
Protons Found = 0 / 23
2k
0.993
1.011
4.192
2.238
3.052
0.995
1.028
1.000
3.168
9.598
Chemical Shift (ppm)

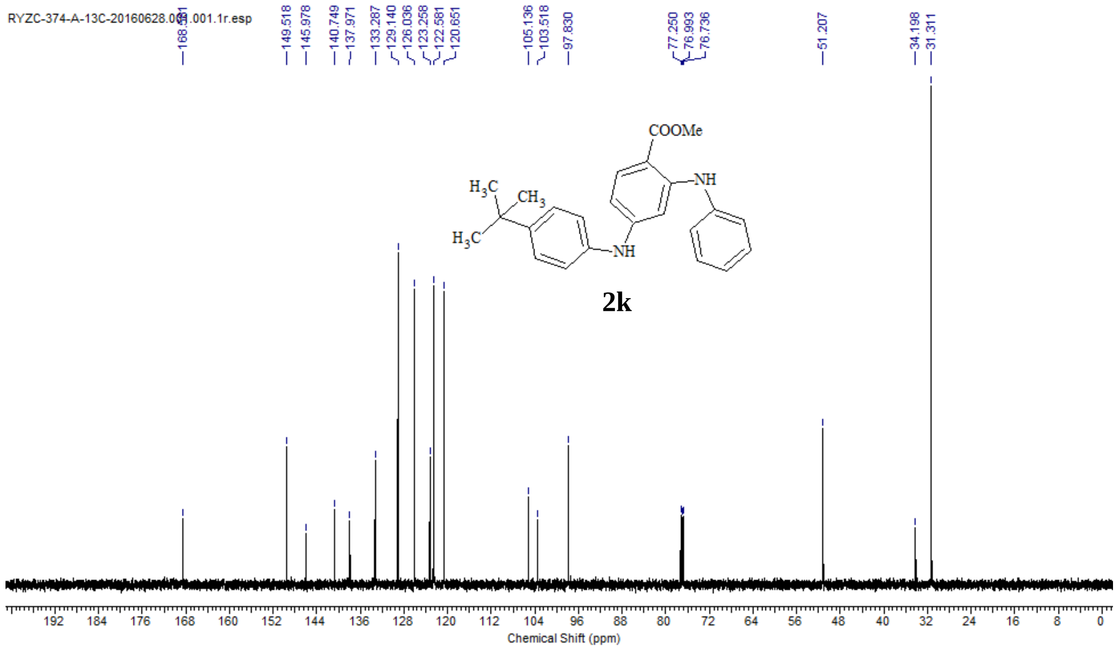
RYZC-374-A-13C-20160628.001.001.1r.esp
168.591
149.518
145.978
140.749
137.971
133.287
129.140
126.036
123.258
122.581
120.651
105.136
103.518
97.830
77.250
76.993
76.736
51.207
34.198
31.311
COOMe
NH
H3C
CH3
H3C
NH
2k
Chemical Shift (ppm)

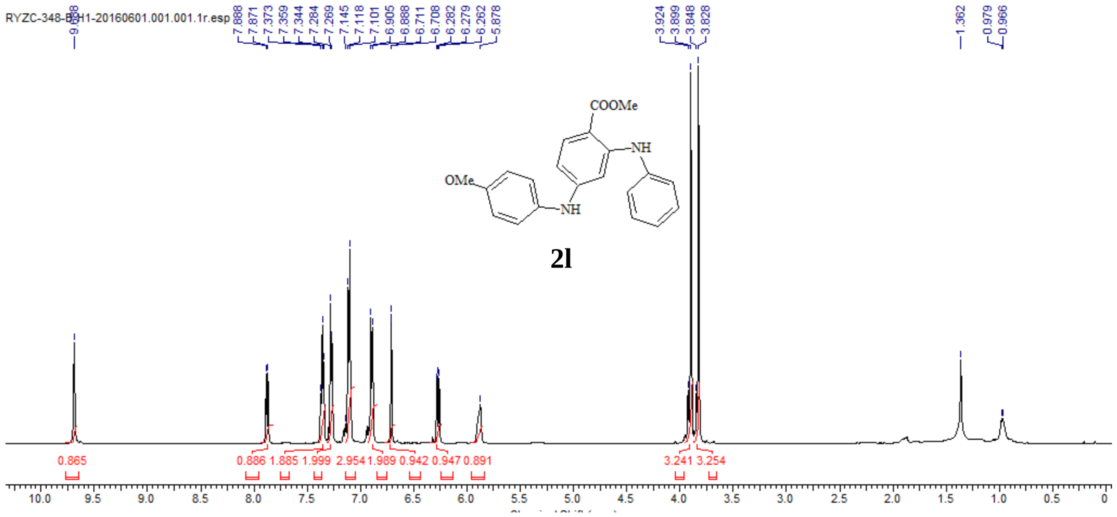
RYZC-348-B-H1-20160601.001.001.1r.esp
COOMe
NH
OMe
NH
2l

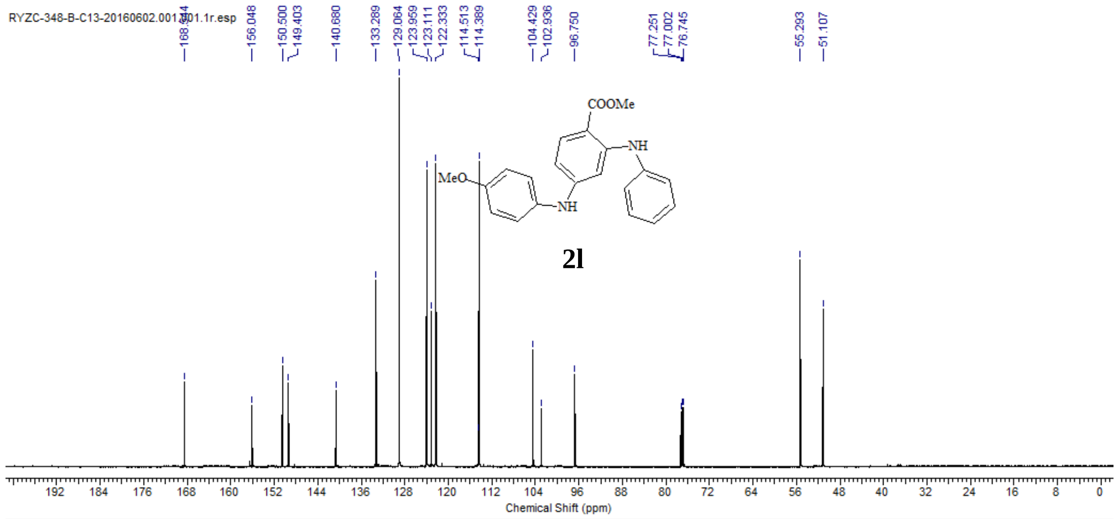
RYZC-348-B-C13-20160602.001.001.1r.esp
COOMe
NH
MeO
NH
2l
Chemical Shift (ppm)

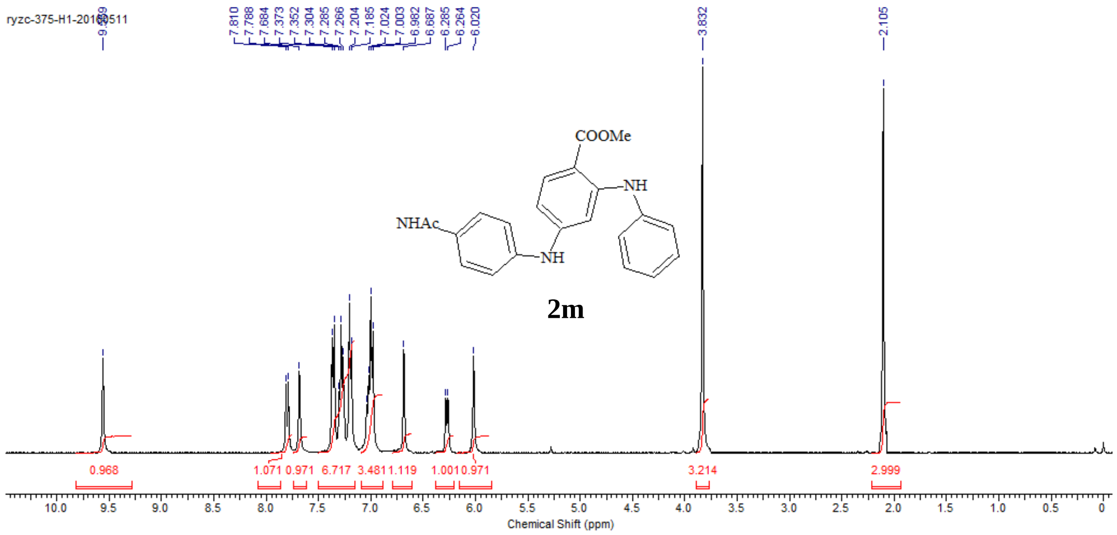
ryzc-375-H1-20160511
COOMe
NH
NHAc
NH
2m
Chemical Shift (ppm)

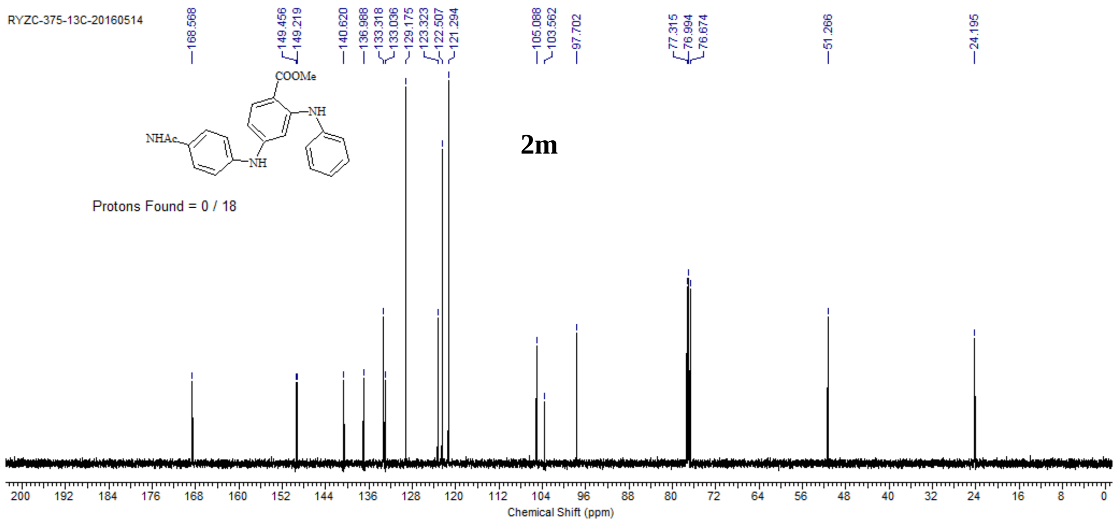
RYZC-375-13C-20160514
168.568
149.456
149.219
140.620
136.988
133.318
133.036
129.175
123.323
122.507
121.294
105.088
103.562
97.702
77.315
76.994
76.674
51.266
24.195
COOMe
NH
NHAc
NH
2m
Protons Found = 0 / 18
Chemical Shift (ppm)

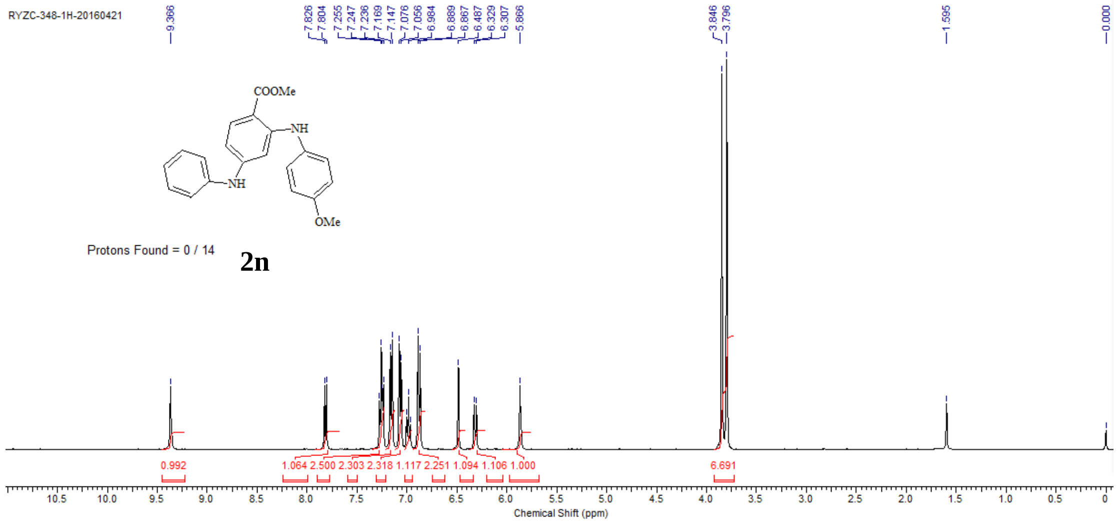
RYZC-348-1H-20160421
9.366
7.826
7.804
7.255
7.247
7.236
7.169
7.147
7.076
7.056
6.984
6.889
6.867
6.487
6.329
6.307
5.866
3.846
3.796
1.595
0.000
COOMe
NH
NH
OMe
Protons Found = 0 / 14
2n
0.992
1.064
2.500
2.303
2.318
1.117
2.251
1.094
1.106
1.000
6.691
Chemical Shift (ppm)

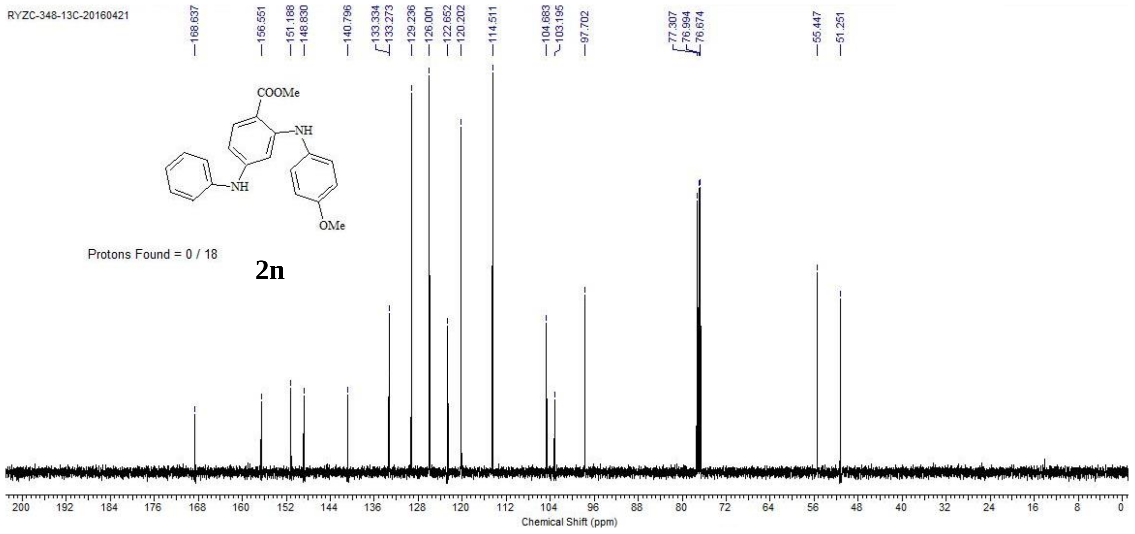
RYZC-348-13C-20160421
168.637
156.551
151.188
148.830
140.796
133.334
133.273
129.236
126.001
122.652
120.202
114.511
104.683
103.195
97.702
77.307
76.994
76.674
55.447
51.251
COOMe
NH
NH
OMe
Protons Found = 0 / 18
2n
Chemical Shift (ppm)

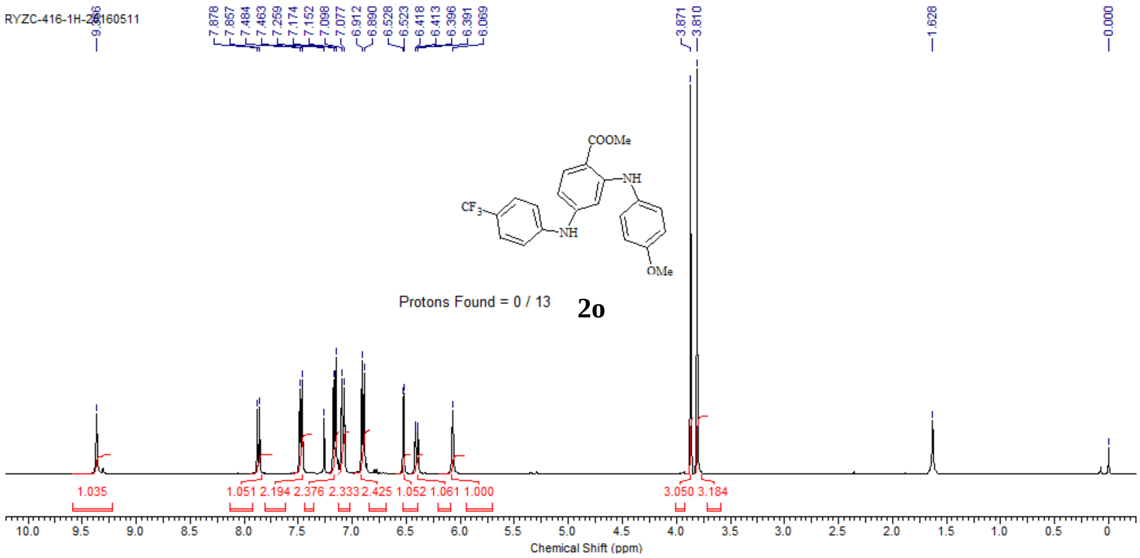
RYZC-416-1H-20160511
7.878
7.857
7.484
7.463
7.259
7.174
7.152
7.098
7.077
6.912
6.890
6.528
6.523
6.418
6.413
6.396
6.391
6.069
3.871
3.810
1.628
0.000
COOMe
NH
CF3
NH
OMe
Protons Found = 0 / 13
2o
1.035
1.051 2.194 2.376 2.333 2.425 1.052 1.061 1.000
3.050 3.184
Chemical Shift (ppm)

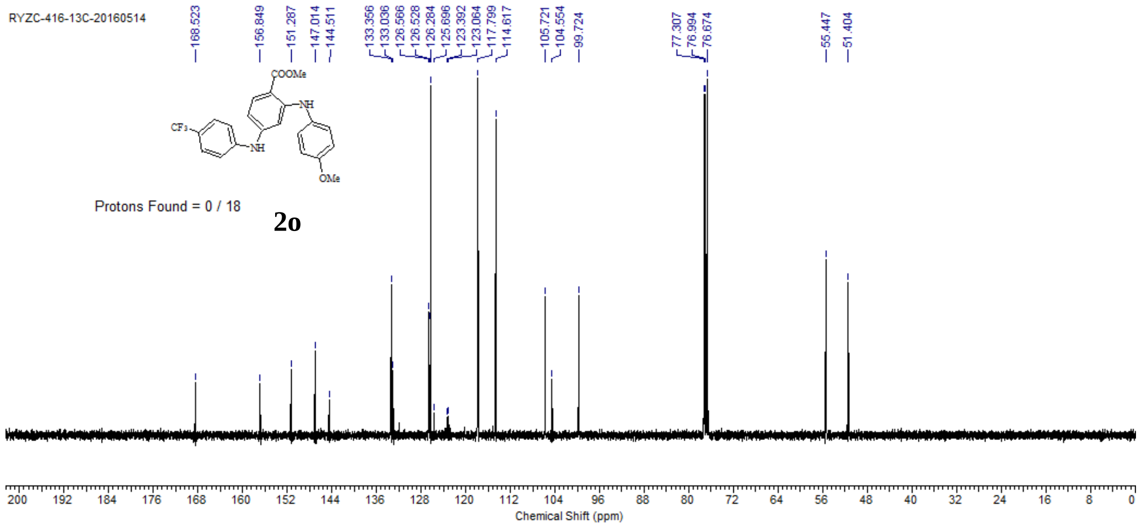
RYZC-416-13C-20160514
168.523
156.849
151.287
147.014
144.511
133.356
133.036
126.566
126.528
126.284
125.696
123.392
123.064
117.799
114.617
105.721
104.554
99.724
77.307
76.994
76.674
55.447
51.404
COOMe
NH
CF3
NH
OMe
Protons Found = 0 / 18
2o
Chemical Shift (ppm)

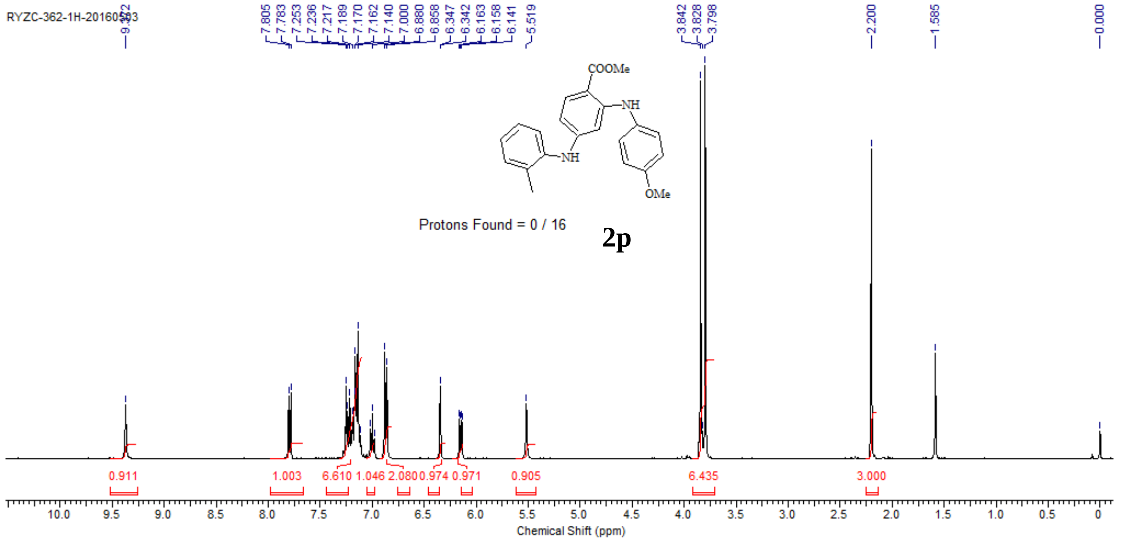
RYZC-362-1H-20160503
7.805
7.783
7.253
7.236
7.217
7.189
7.170
7.162
7.140
7.000
6.880
6.858
6.347
6.342
6.163
6.158
6.141
5.519
3.842
3.828
3.798
2.200
1.585
0.000
COOMe
NH
NH
OMe
Protons Found = 0 / 16
2p
0.911
1.003
6.610 1.046 2.080 0.974 0.971
0.905
6.435
3.000
Chemical Shift (ppm)

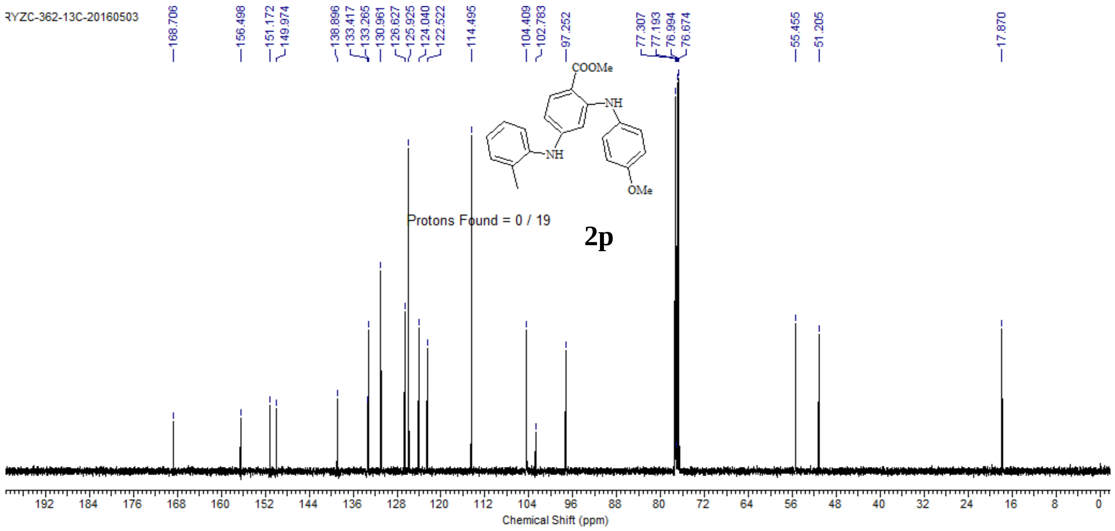
RYZC-362-13C-20160503
168.706
156.498
151.172
149.974
138.896
133.417
133.265
130.961
126.627
125.925
124.040
122.522
114.495
104.409
102.783
97.252
77.307
77.193
76.994
76.674
55.455
51.205
17.870
COOMe
NH
NH
OMe
Protons Found = 0 / 19
2p
Chemical Shift (ppm)

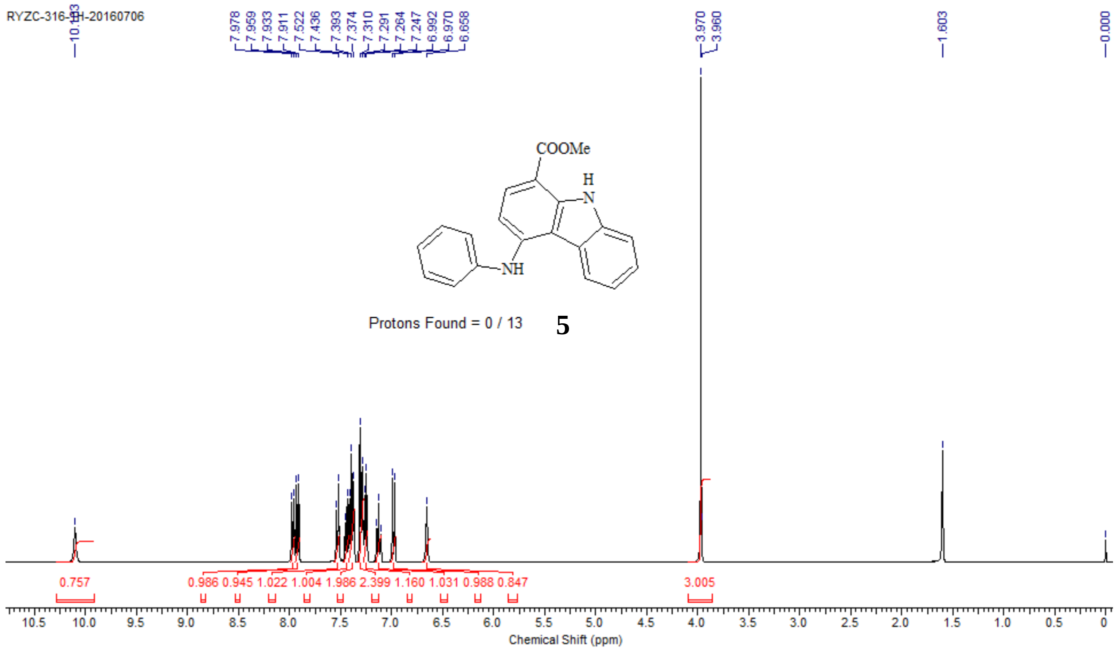
RYZC-316-1H-20160706
10.103
7.978
7.959
7.933
7.911
7.522
7.436
7.393
7.374
7.310
7.291
7.264
7.247
6.992
6.970
6.658
3.970
3.960
1.603
0.000
COOMe
H
N
NH
Protons Found = 0 / 13
5
0.757
0.986 0.945 1.022 1.004 1.986 2.399 1.160 1.031 0.988 0.847
3.005
Chemical Shift (ppm)

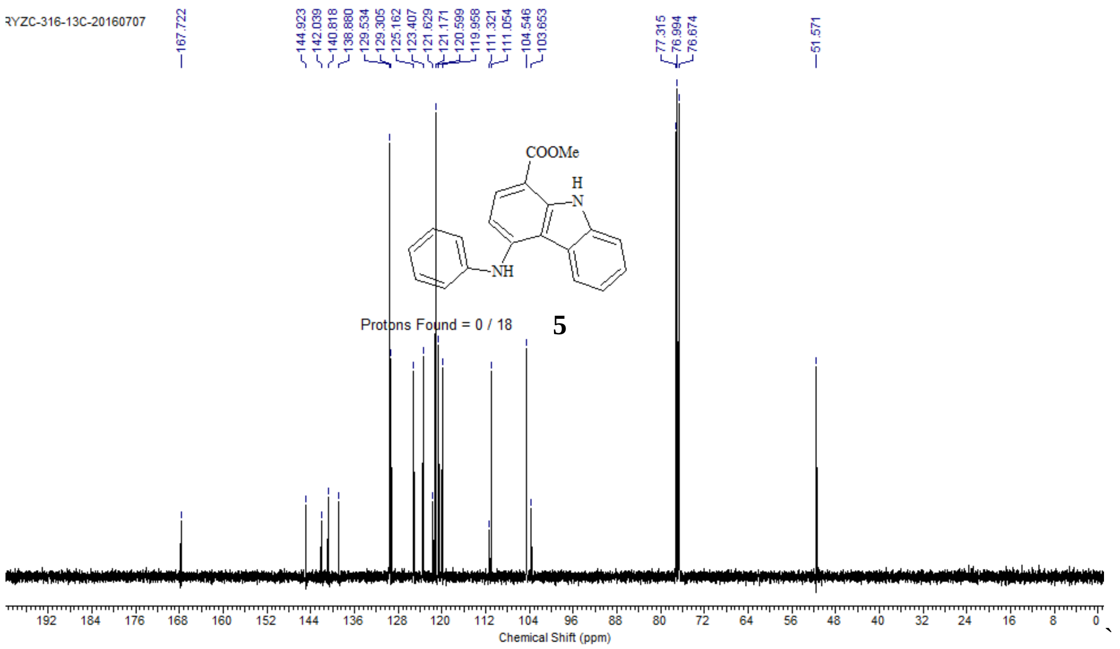
RYZC-316-13C-20160707
167.722
144.923
142.039
140.818
138.880
129.534
129.305
125.162
123.407
121.629
121.171
120.599
119.958
111.321
111.054
104.546
103.653
77.315
76.994
76.674
51.571
COOMe
H
N
NH
Protons Found = 0 / 18
5
Chemical Shift (ppm)

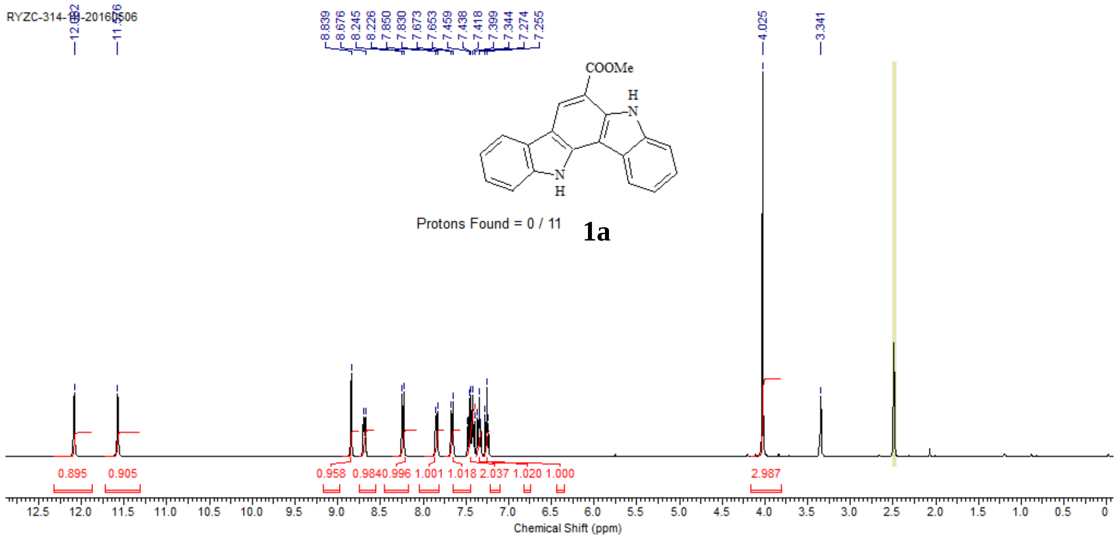
8.839
8.676
8.245
8.226
7.850
7.830
7.673
7.653
7.459
7.438
7.418
7.399
7.344
7.274
7.255
4.025
3.341
COOMe
H
N
N
H
Protons Found = 0 / 11
1a
0.895
0.905
0.958
0.984
0.996
1.001
1.018
2.037
1.020
1.000
2.987
Chemical Shift (ppm)

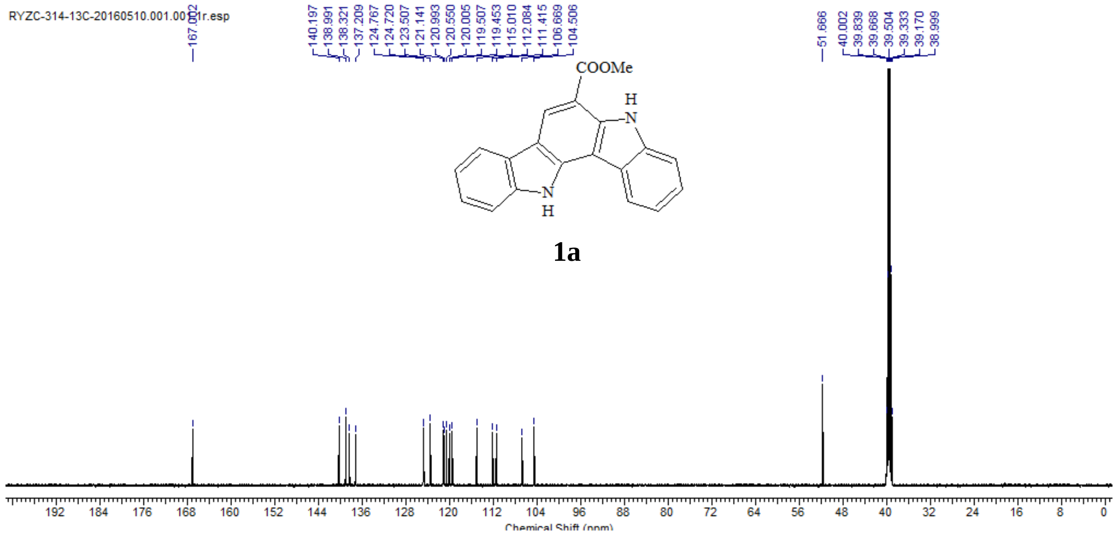
140.197
138.991
138.321
137.209
124.767
124.720
123.507
121.141
120.993
120.550
120.005
119.507
119.453
115.010
112.084
111.415
106.669
104.506
51.666
40.002
39.839
39.668
39.504
39.333
39.170
38.999
COOMe
H
N
N
H
1a
Chemical Shift (ppm)

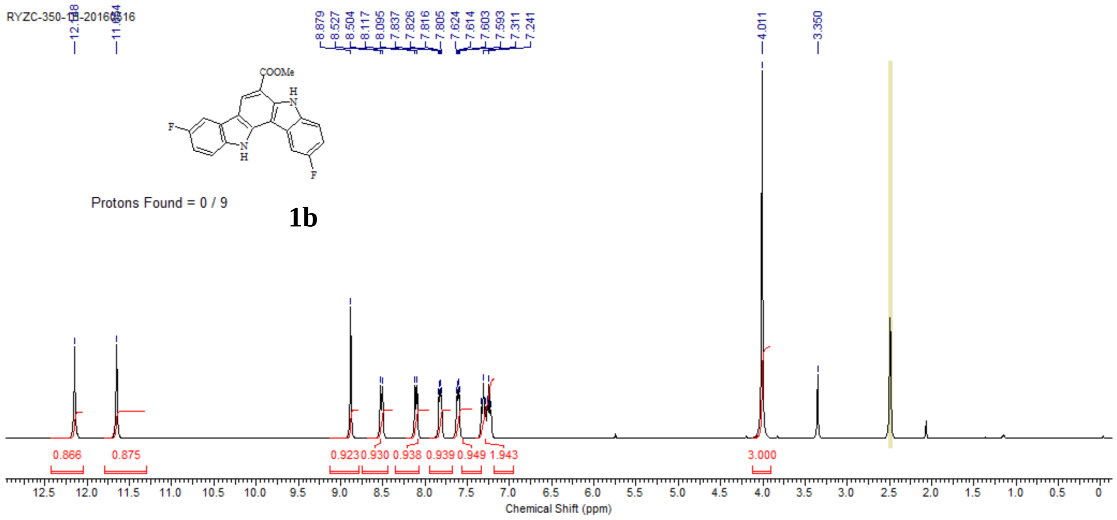
RYZC-350-
8.879 8.527 8.504 8.117 8.095 7.837 7.826 7.816 7.805 7.624 7.614 7.603 7.593 7.311 7.241
4.011
3.350
COOMe
Protons Found = 0 / 9
0.866 0.875 0.923 0.930 0.938 0.939 0.949 1.943 3.000
Chemical Shift (ppm)

1b

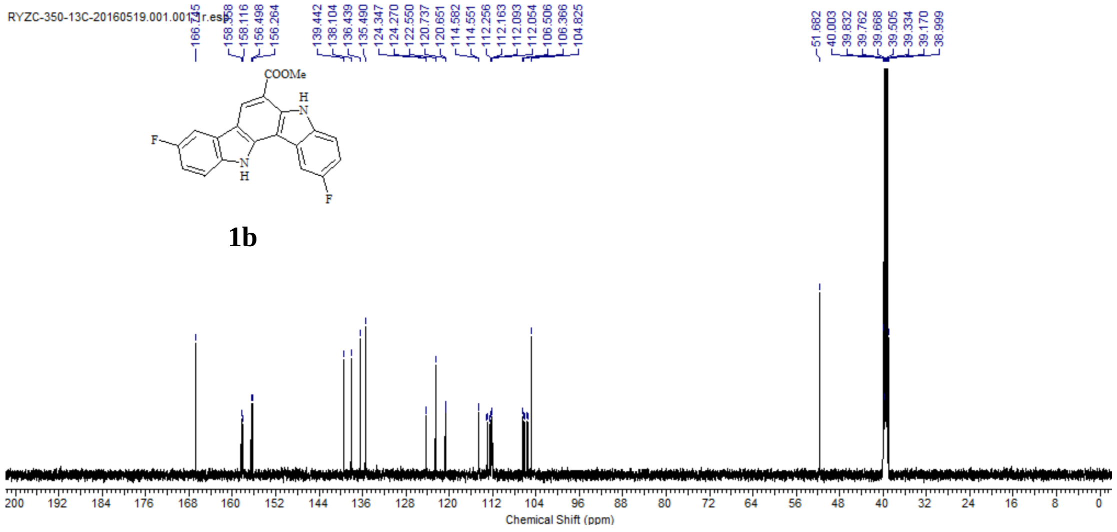
RYZC-350-13C-20160519.001.001
158.358 158.116 156.498 156.264
139.442 138.104 136.439 135.490 124.347 124.270 122.550 120.737 120.651 114.582 114.551 112.256 112.163 112.093 112.054 106.506 106.366 104.825
51.682 40.003 39.832 39.762 39.668 39.505 39.334 39.170 38.999
COOMe
Chemical Shift (ppm)

1b

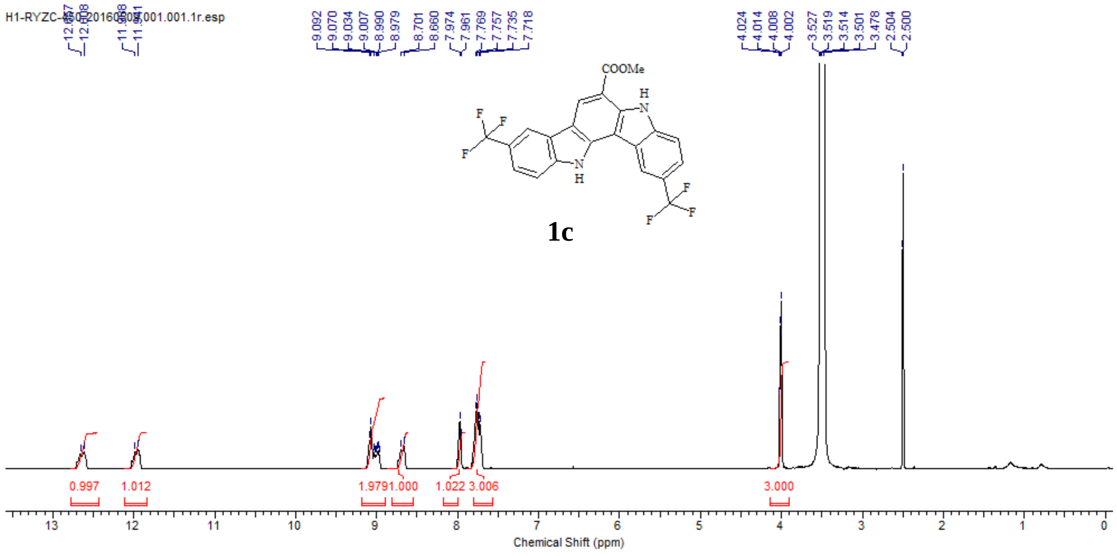
H1-RYZC-
9.092 9.070 9.034 9.007 8.990 8.979 8.701 8.660 7.974 7.961 7.769 7.757 7.735 7.718
4.024 4.014 4.008 4.002 3.527 3.519 3.514 3.501 3.478 2.504 2.500
COOMe
0.997 1.012 1.979 1.000 1.022 3.006 3.000
Chemical Shift (ppm)

1c

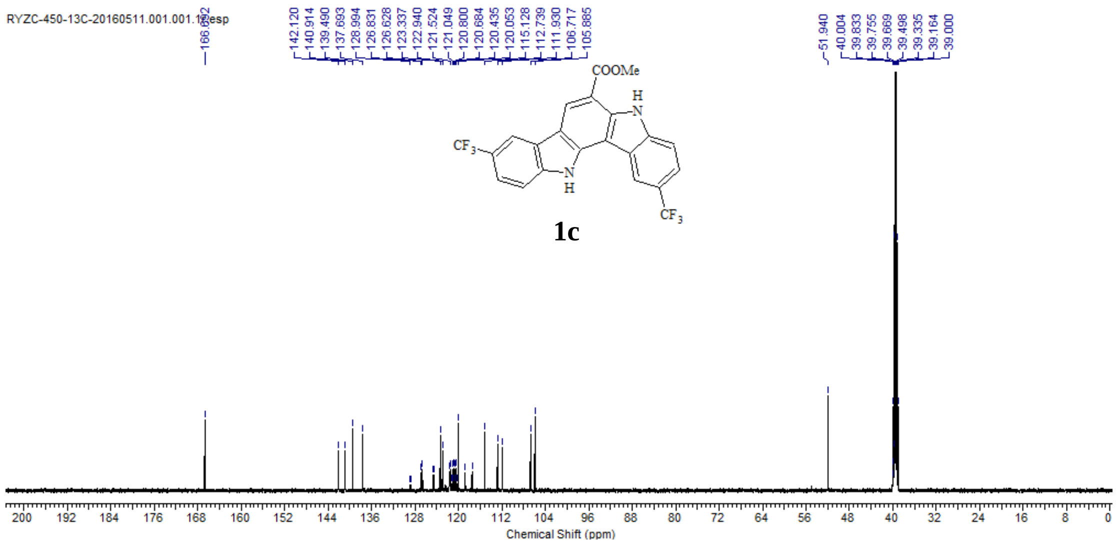
RYZC-450-13C-20160511.001.001.1r.esp
166.962
142.120
140.914
139.490
137.693
128.994
126.831
126.628
123.337
122.940
121.524
121.049
120.800
120.684
120.435
120.053
115.128
112.739
111.930
106.717
105.885
51.940
40.004
39.833
39.755
39.669
39.498
39.335
39.164
39.000
COOMe
H
N
CF3
N
H
CF3
1c
Chemical Shift (ppm)

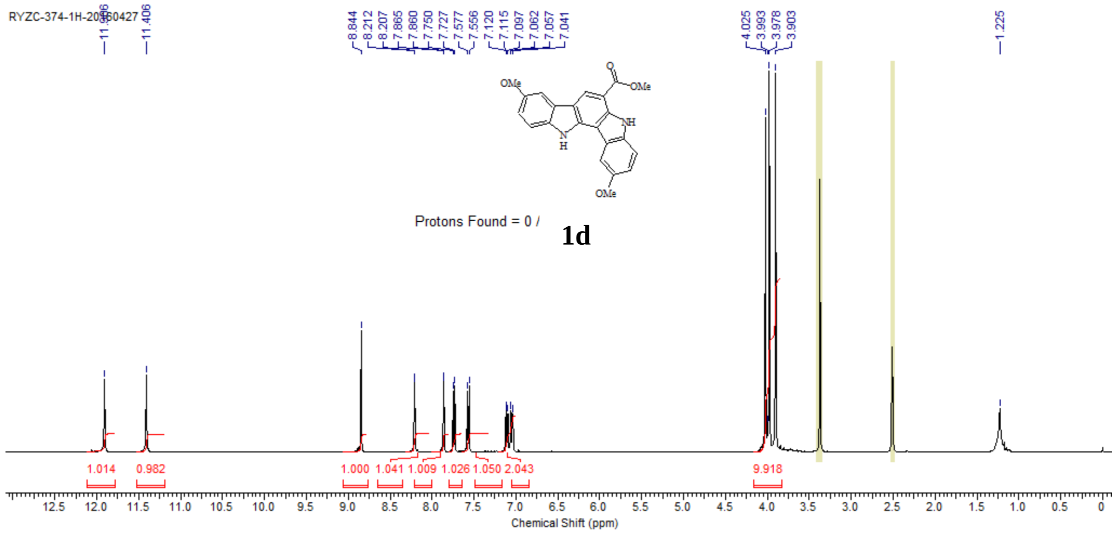
RYZC-374-1H-20160427
11.406
8.844
8.212
8.207
7.865
7.860
7.750
7.727
7.577
7.556
7.120
7.115
7.097
7.062
7.057
7.041
4.025
3.993
3.978
3.903
1.225
OMe
OMe
NH
N
H
OMe
Protons Found = 0 /
1d
1.014
0.982
1.000
1.041
1.009
1.026
1.050
2.043
9.918
Chemical Shift (ppm)

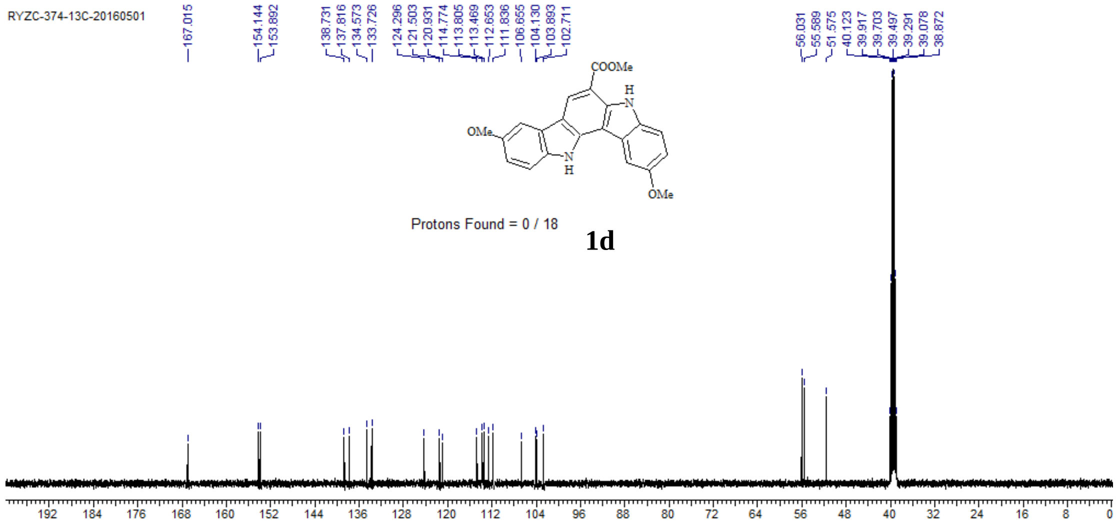
RYZC-374-13C-20160501
167.015
154.144
153.892
138.731
137.816
134.573
133.726
124.296
121.503
120.931
114.774
113.805
113.469
112.653
111.836
106.655
104.130
103.893
102.711
56.031
55.589
51.575
40.123
39.917
39.703
39.497
39.291
39.078
38.872
COOMe
H
N
OMe
N
H
OMe
Protons Found = 0 / 18
1d

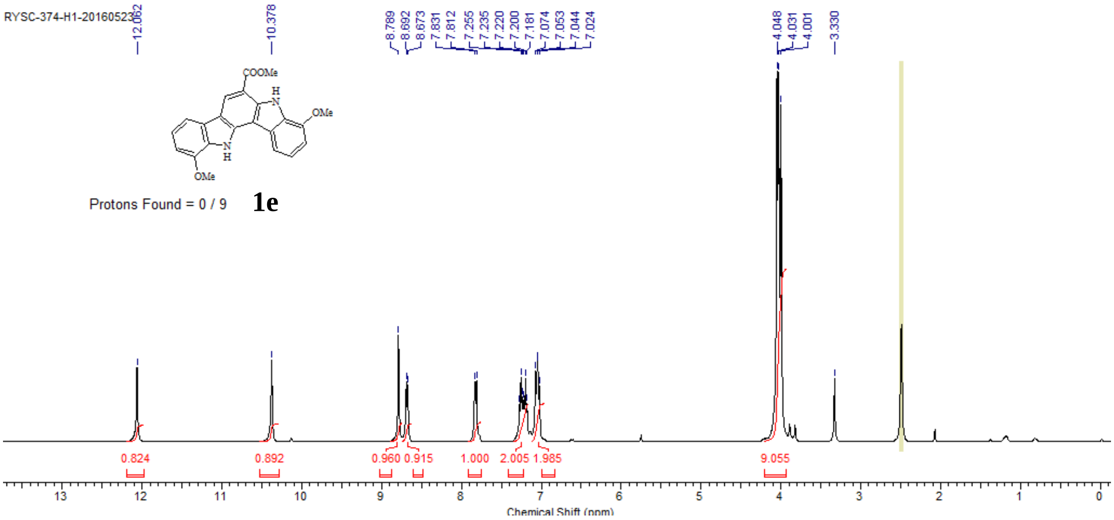
RYSC-374-H1-20160523
12.062
10.378
8.789
8.692
8.673
7.831
7.812
7.255
7.235
7.220
7.200
7.181
7.074
7.053
7.044
7.024
4.048
4.031
4.001
3.330
COOMe
H
N
OMe
N
H
OMe
Protons Found = 0 / 9
1e
0.824
0.892
0.960
0.915
1.000
2.005
1.985
9.055
Chemical Shift (ppm)

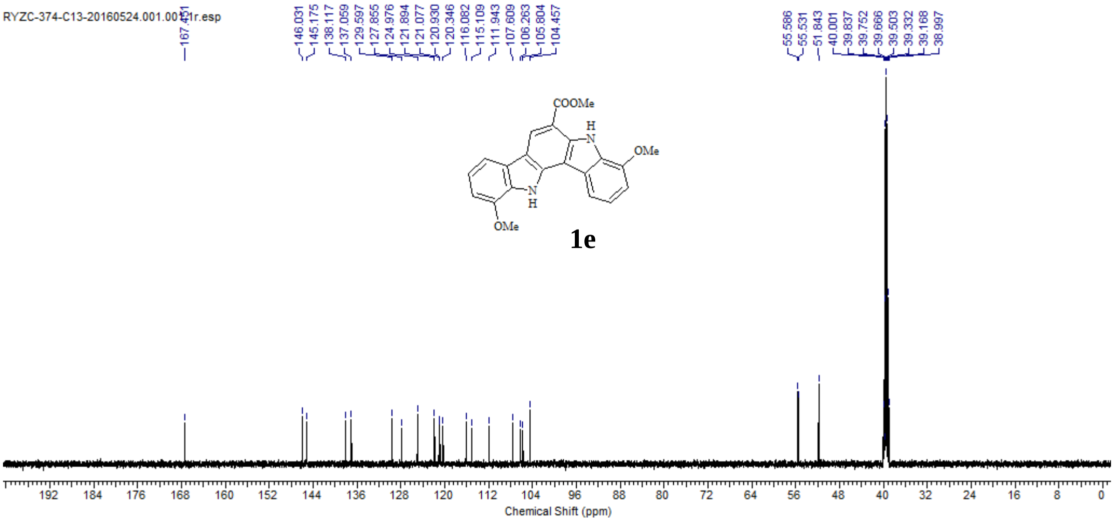
RYZC-374-C13-20160524.001.001.1r.esp
167.401
146.031
145.175
138.117
137.059
129.597
127.855
124.976
121.894
121.077
120.930
120.346
116.082
115.109
111.943
107.609
106.263
105.804
104.457
55.586
55.531
51.843
40.001
39.837
39.752
39.666
39.503
39.332
39.168
38.997
COOMe
H
N
OMe
N
H
OMe
1e
Chemical Shift (ppm)

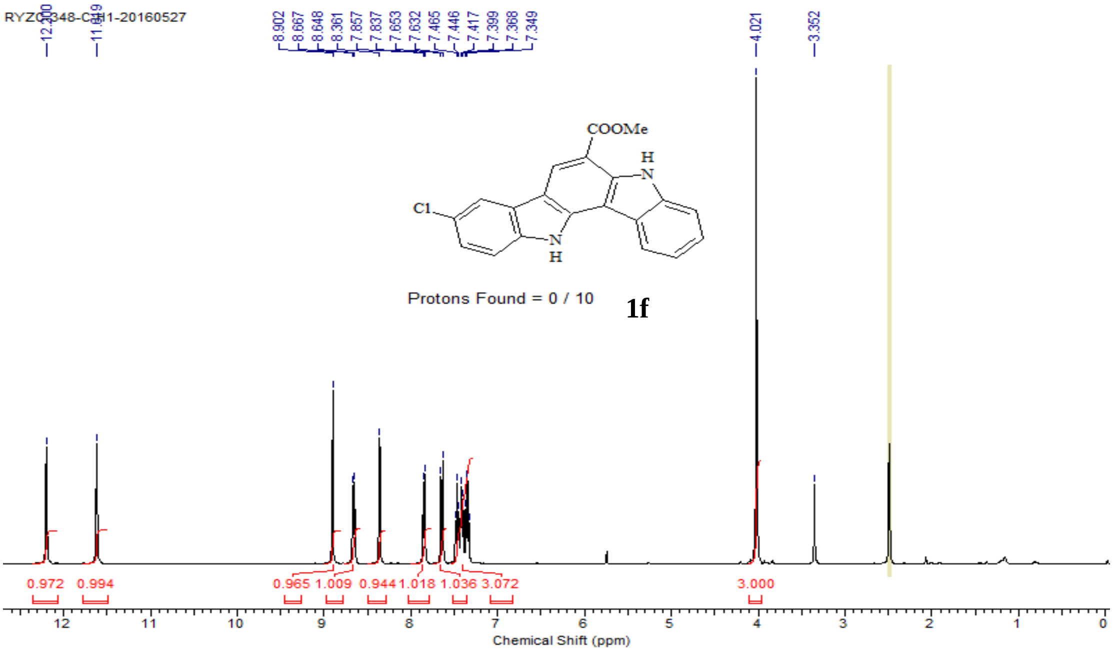
RYZC-348-C-H1-20160527
12.200
11.619
8.902
8.667
8.648
8.361
7.857
7.837
7.653
7.632
7.465
7.446
7.417
7.399
7.368
7.349
4.021
3.352
COOMe
H
N
Cl
N
H
Protons Found = 0 / 10
1f
0.972
0.994
0.965
1.009
0.944
1.018
1.036
3.072
3.000
Chemical Shift (ppm)

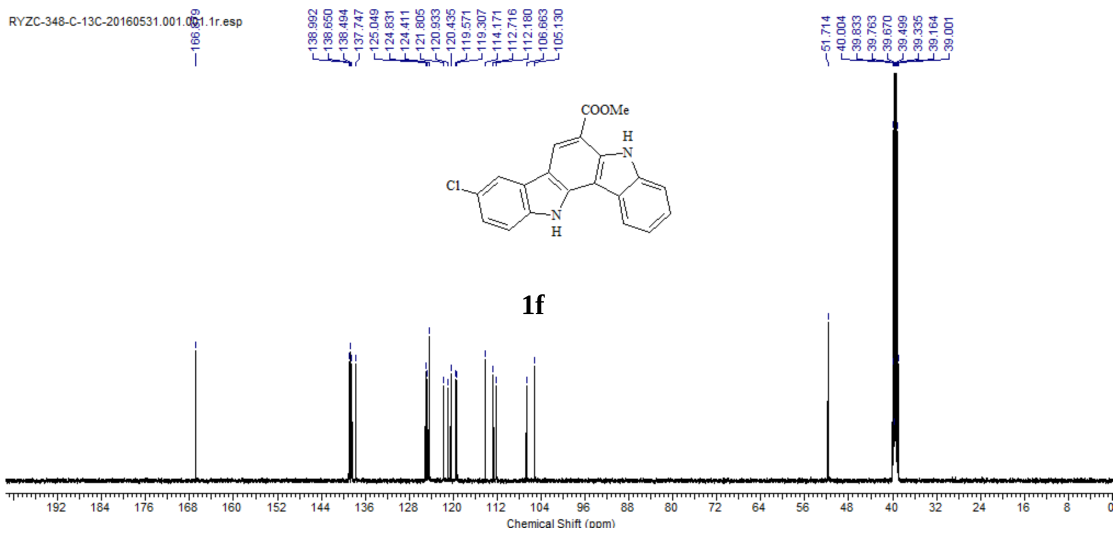
RYZC-348-C-13C-20160531.001.001.1r.esp
166.879
138.992
138.650
138.494
137.747
125.049
124.831
124.411
121.805
120.933
120.435
119.571
119.307
114.171
112.716
112.180
106.663
105.130
51.714
40.004
39.833
39.763
39.670
39.499
39.335
39.164
39.001
COOMe
H
N
Cl
N
H
1f
Chemical Shift (ppm)

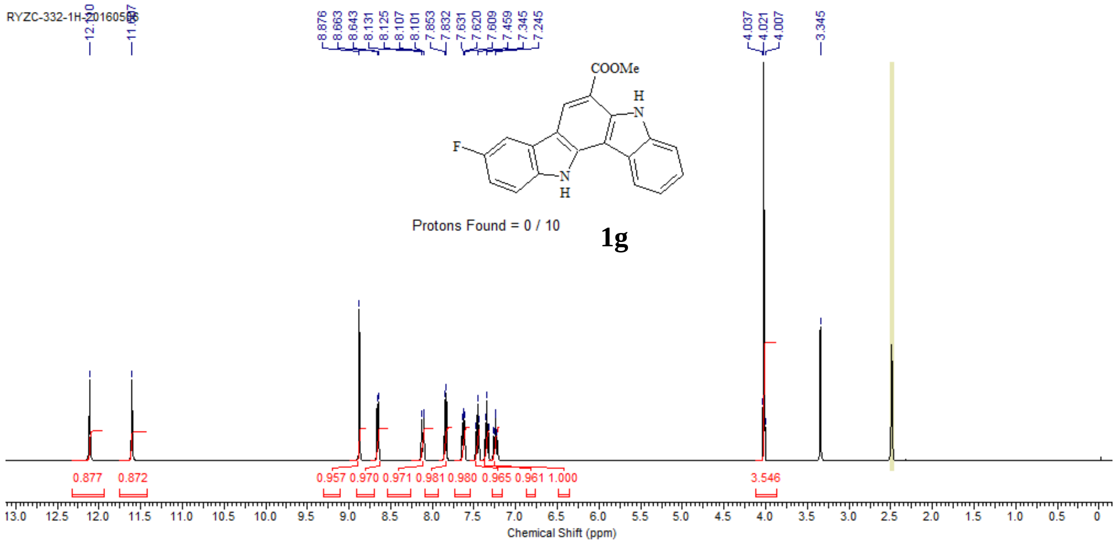
RYZC-332-1H-20160506
12.100
11.567
8.876
8.663
8.643
8.131
8.125
8.107
8.101
7.853
7.832
7.631
7.620
7.609
7.459
7.345
7.245
4.037
4.021
4.007
3.345
COOMe
H
N
F
N
H
Protons Found = 0 / 10
1g
0.877
0.872
0.957
0.970
0.971
0.981
0.980
0.965
0.961
1.000
3.546
Chemical Shift (ppm)

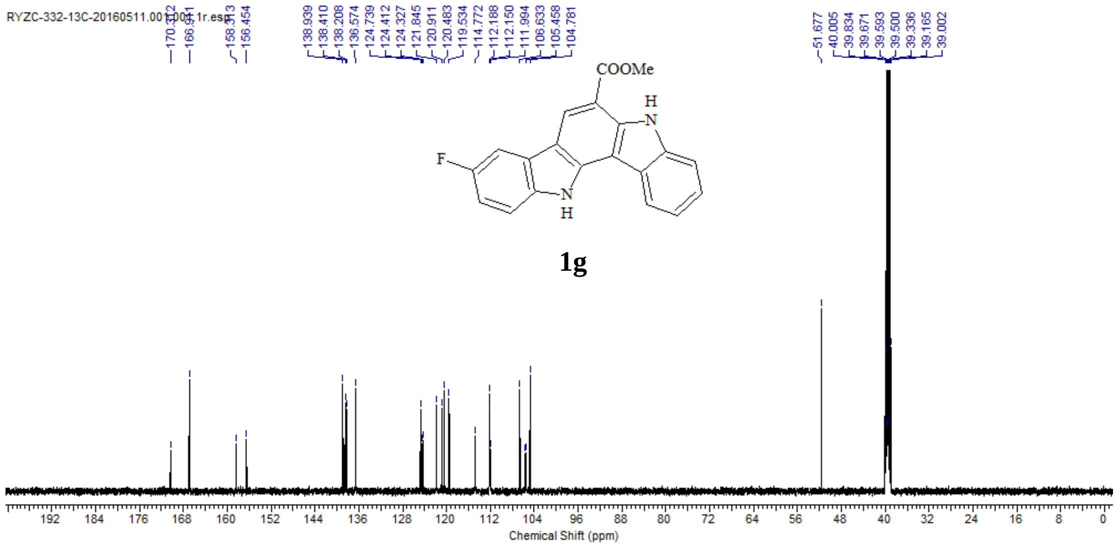
RYZC-332-13C-20160511.001.001.1r.esp
170.332
166.851
158.313
156.454
138.939
138.410
138.208
136.574
124.739
124.412
124.327
121.845
120.911
120.483
119.534
114.772
112.188
112.150
111.994
106.633
105.458
104.781
51.677
40.005
39.834
39.671
39.593
39.500
39.336
39.165
39.002
COOMe
H
N
F
N
H
1g
Chemical Shift (ppm)

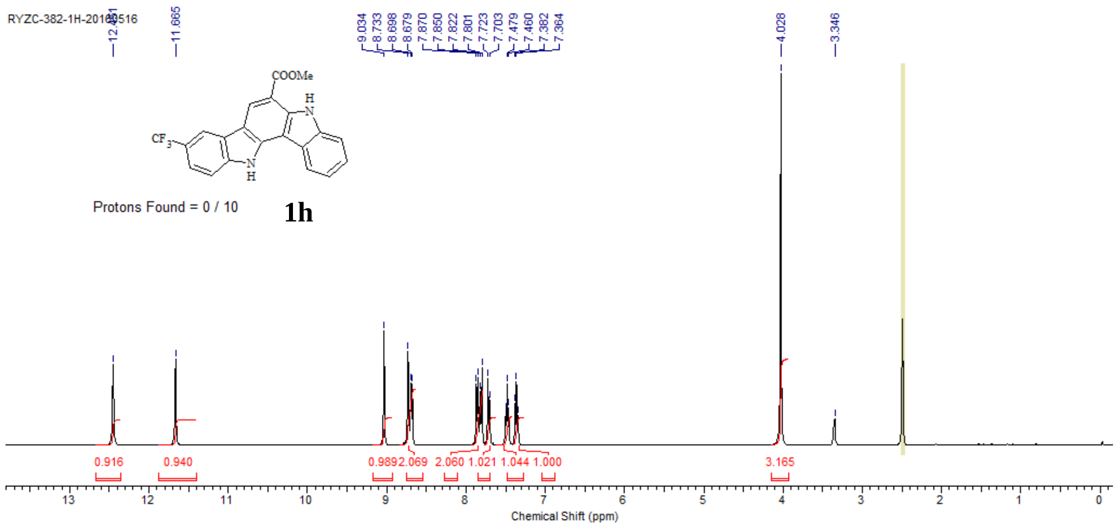
RYZC-382-1H-20160516
12.481
11.665
9.034 8.733 8.698 8.679 7.870 7.850 7.822 7.801 7.723 7.703 7.479 7.460 7.382 7.364
4.028
3.346
COOMe
H
N
CF3
N
H
Protons Found = 0 / 10
1h
0.916 0.940 0.989 2.069 2.060 1.021 1.044 1.000 3.165
Chemical Shift (ppm)

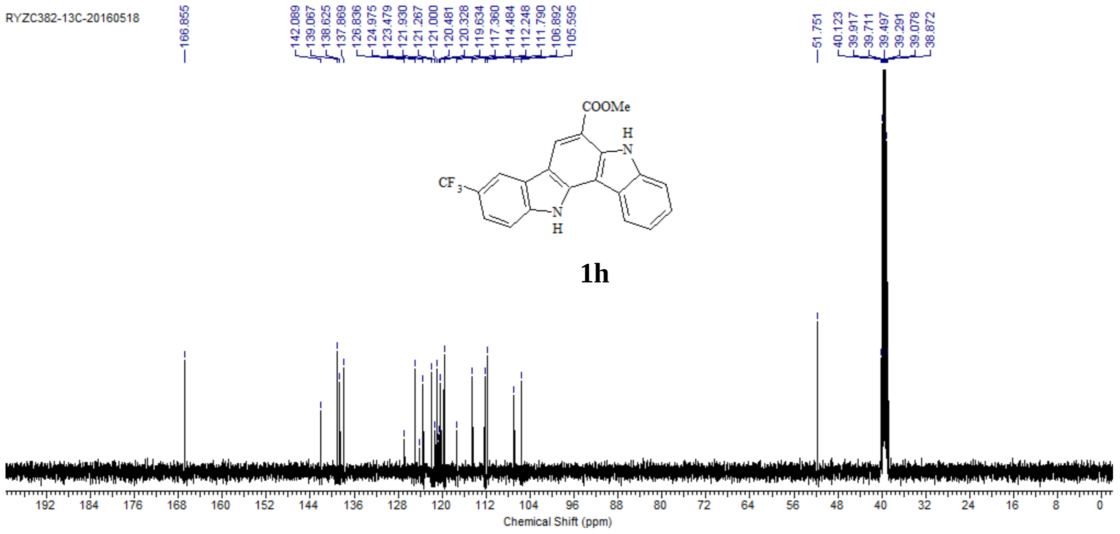
RYZC382-13C-20160518
166.855
142.089 139.067 138.625 137.869 126.836 124.975 123.479 121.930 121.267 121.000 120.481 120.328 119.634 117.360 114.484 112.248 111.790 106.892 105.595
51.751
40.123 39.917 39.711 39.497 39.291 39.078 38.872
COOMe
H
N
CF3
N
H
1h
Chemical Shift (ppm)

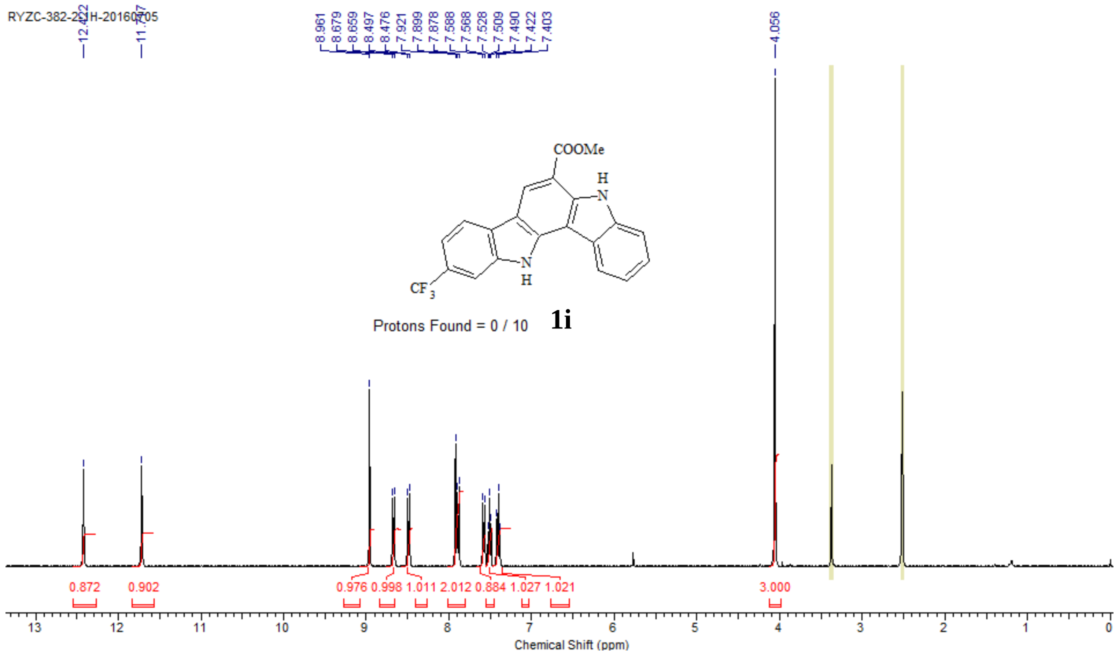
RYZC-382-2-1H-20160705
12.422
11.707
8.961 8.679 8.659 8.497 8.476 7.921 7.899 7.878 7.588 7.568 7.528 7.509 7.490 7.422 7.403
4.056
COOMe
H
N
N
H
CF3
Protons Found = 0 / 10
1i
0.872 0.902 0.976 0.998 1.011 2.012 0.884 1.027 1.021 3.000
Chemical Shift (ppm)

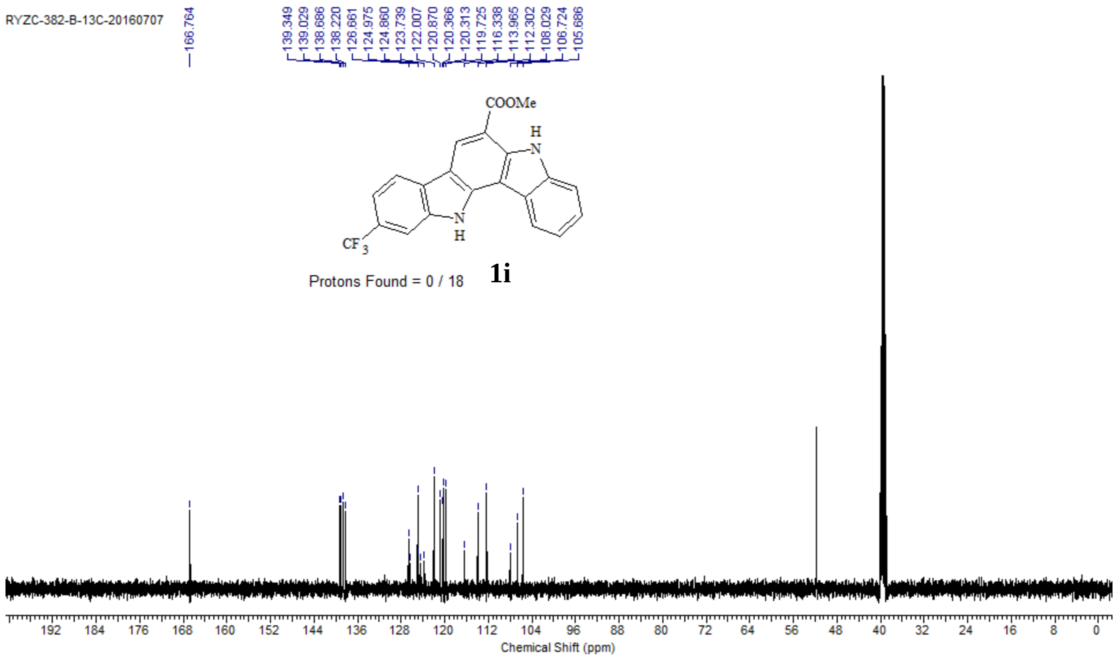
RYZC-382-B-13C-20160707
166.764
139.349
139.029
138.686
138.220
126.661
124.975
124.860
123.739
122.007
120.870
120.366
120.313
119.725
116.338
113.965
112.302
108.029
106.724
105.686
COOMe
H
N
N
H
CF 3
Protons Found = 0 / 18
1i
Chemical Shift (ppm)

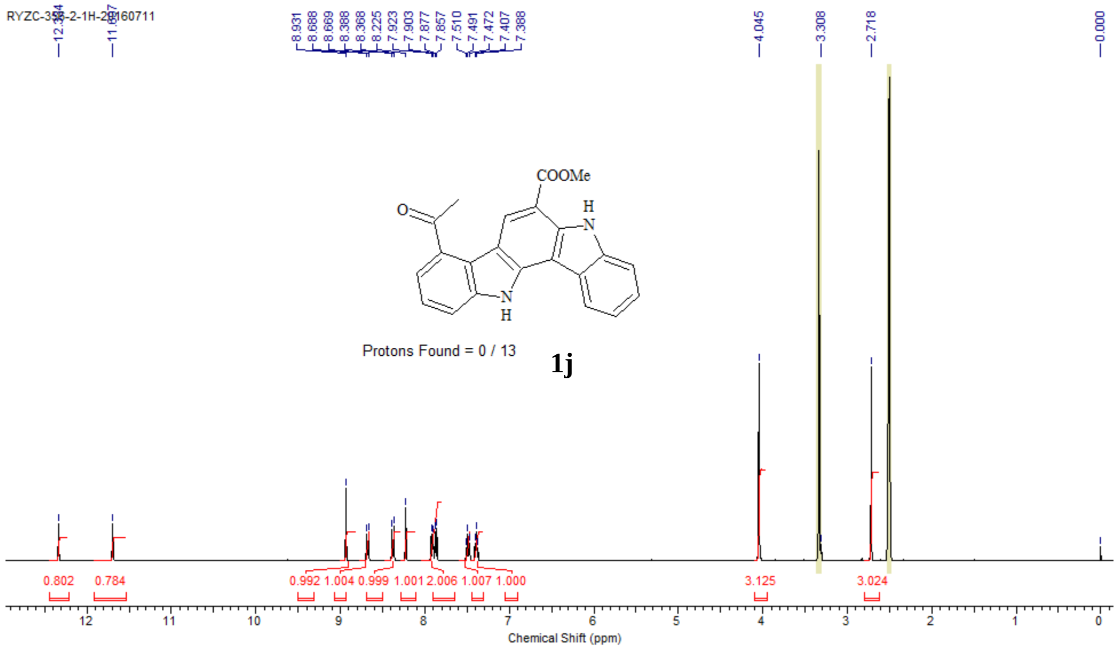
RYZC-384-2-1H-20160711
12.344
11.637
8.931
8.688
8.669
8.388
8.368
8.225
7.923
7.903
7.877
7.857
7.510
7.491
7.472
7.407
7.388
4.045
3.308
2.718
0.000
COOMe
O
H
N
N
H
Protons Found = 0 / 13
1j
0.802
0.784
0.992
1.004
0.999
1.001
2.006
1.007
1.000
3.125
3.024
Chemical Shift (ppm)

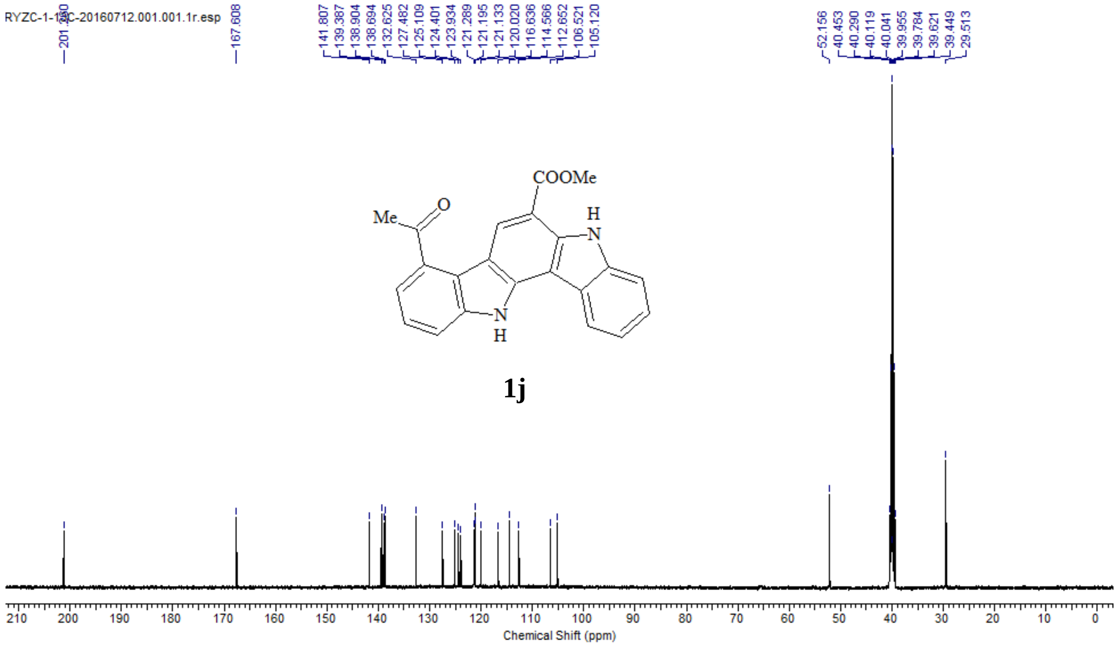
RYZC-1-1-C-20160712.001.001.1r.esp
1j
Chemical Shift (ppm)

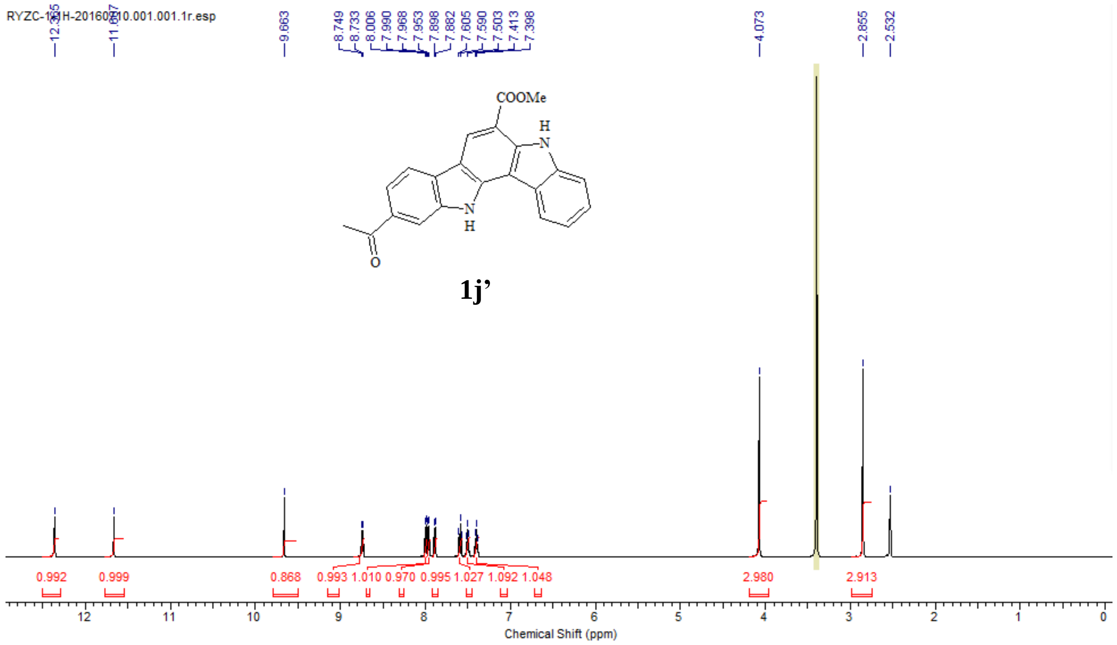
RYZC-1-1-H-20160710.001.001.1r.esp
1j'
Chemical Shift (ppm)

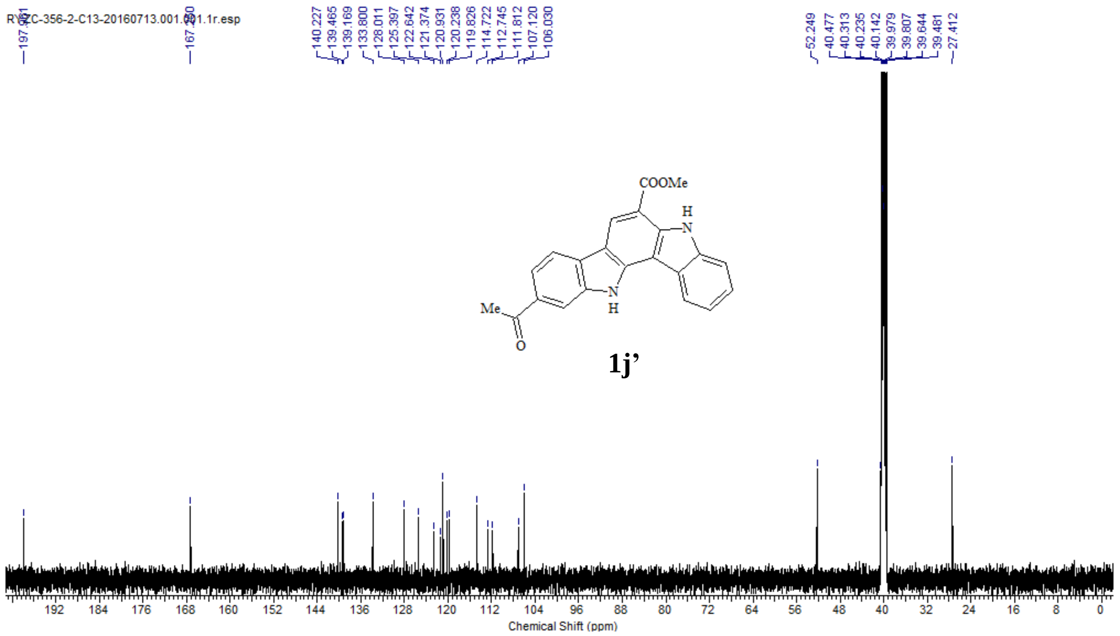
RYZC-356-2-C13-20160713.001.001.1r.esp
197.[illegible]
167.[illegible]
140.227
139.465
139.169
133.800
128.011
125.397
122.642
121.374
120.931
120.238
119.826
114.722
112.745
111.812
107.120
106.030
52.249
40.477
40.313
40.235
40.142
39.979
39.807
39.644
39.481
27.412
COOMe
H
N
Me
O
N
H
1j'
Chemical Shift (ppm)

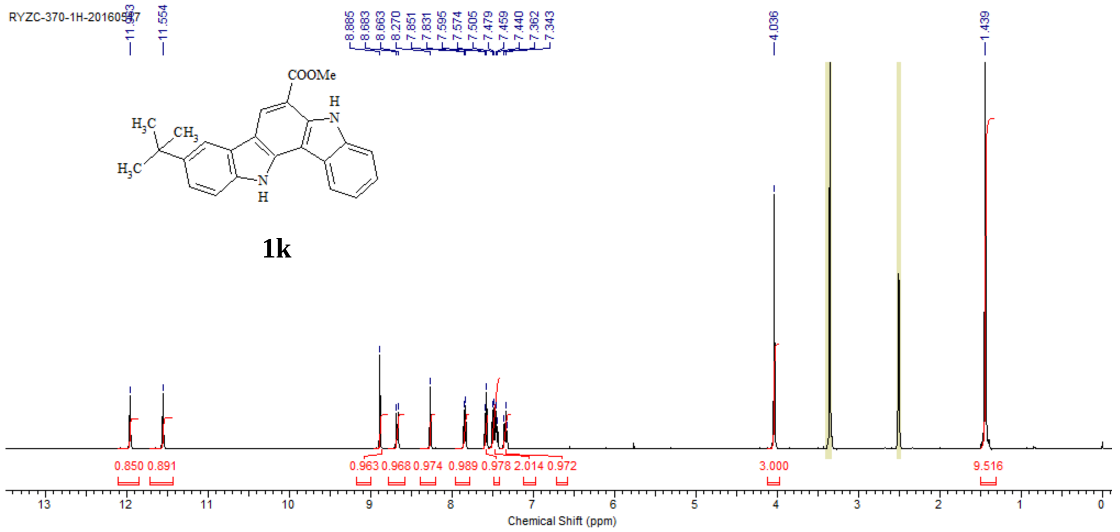
RYZC-370-1H-20160517
11.9[illegible]
11.554
8.885
8.683
8.663
8.270
7.851
7.831
7.595
7.574
7.505
7.479
7.459
7.440
7.362
7.343
4.036
1.439
COOMe
H
N
N
H
1k
0.850 0.891
0.963 0.968 0.974 0.989 0.978 2.014 0.972
3.000
9.516
Chemical Shift (ppm)

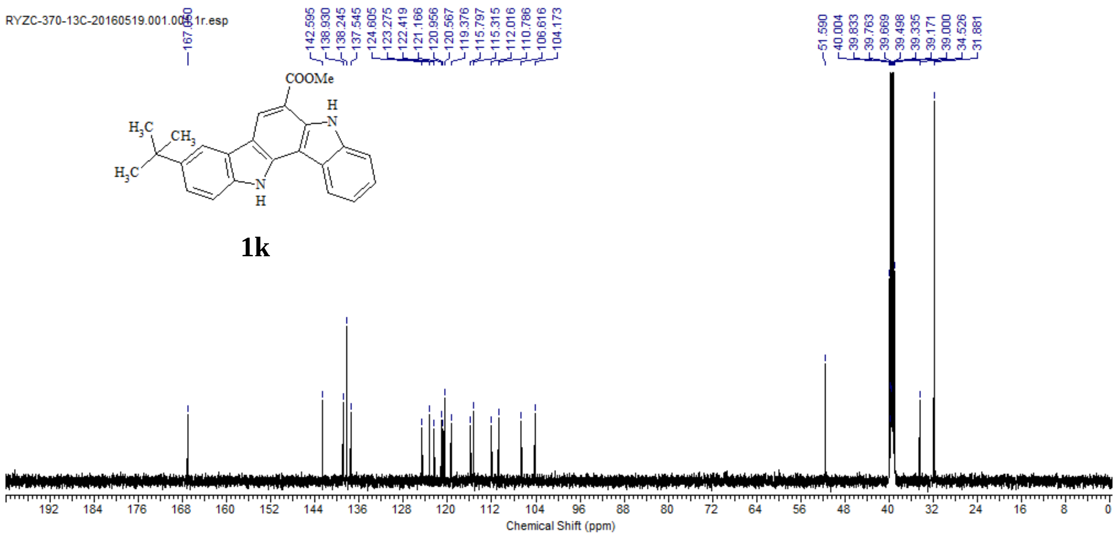
RYZC-370-13C-20160519.001.001.1r.esp
167.[illegible]
142.595
138.930
138.245
137.545
124.605
123.275
122.419
121.166
120.956
120.567
119.376
115.797
115.315
112.016
110.786
106.616
104.173
51.590
40.004
39.833
39.763
39.669
39.498
39.335
39.171
39.000
34.526
31.881
COOMe
H
N
N
H
1k
Chemical Shift (ppm)

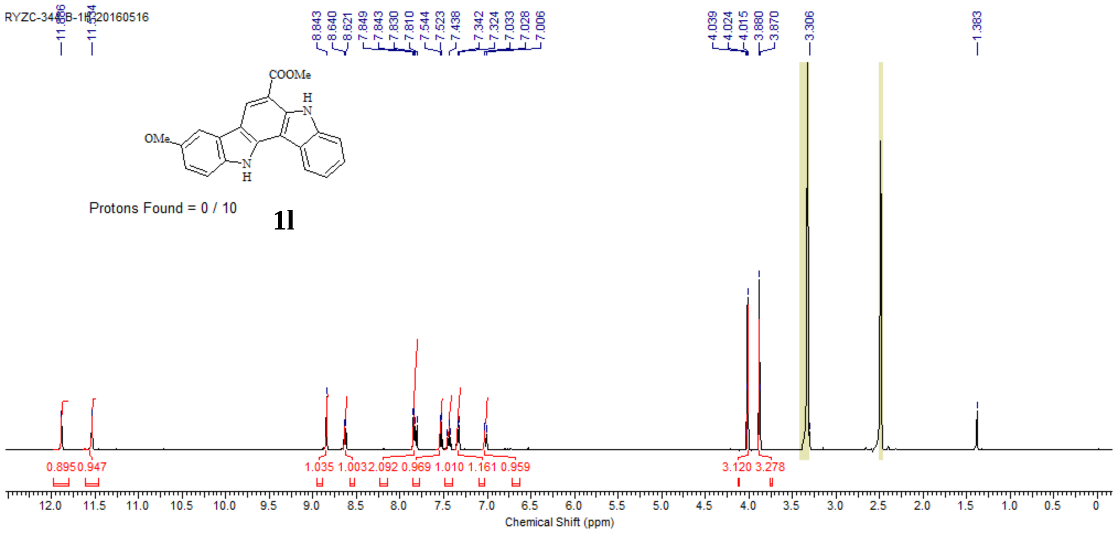
RYZC-344-B-1H-20160516
COOMe
OMe
H
N
Protons Found = 0 / 10
1l
8.843 8.640 8.621 7.849 7.843 7.830 7.810 7.544 7.523 7.438 7.342 7.324 7.033 7.028 7.006
4.039 4.024 4.015 3.880 3.870
3.306
1.383
0.895 0.947 1.035 1.003 2.092 0.969 1.010 1.161 0.959 3.120 3.278
Chemical Shift (ppm)

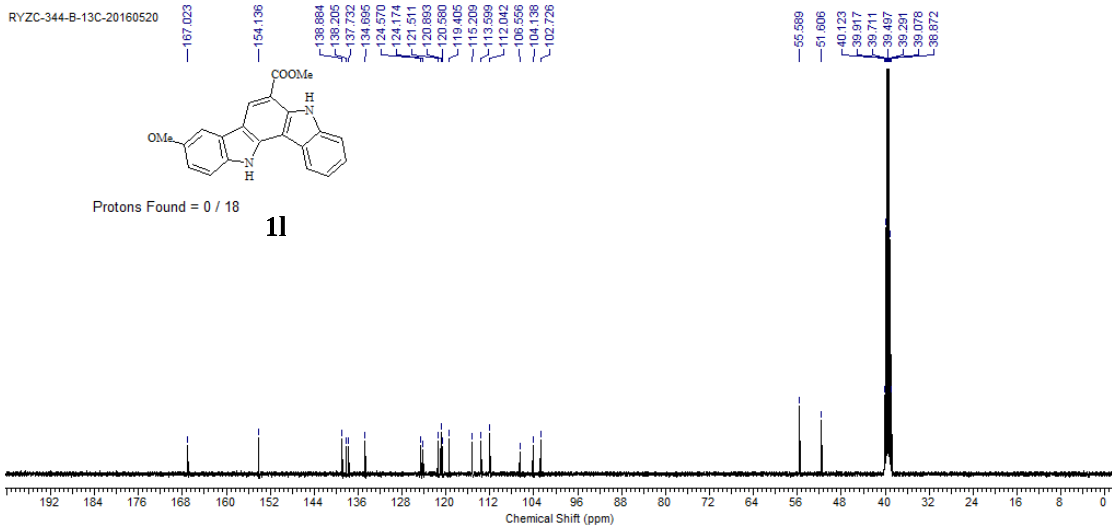
RYZC-344-B-13C-20160520
167.023
154.136
138.884 138.205 137.732 134.695 124.570 124.174 121.511 120.893 120.580 119.405 115.209 113.599 112.042 106.556 104.138 102.726
55.589 51.606 40.123 39.917 39.711 39.497 39.291 39.078 38.872
COOMe
OMe
H
N
Protons Found = 0 / 18
1l
Chemical Shift (ppm)

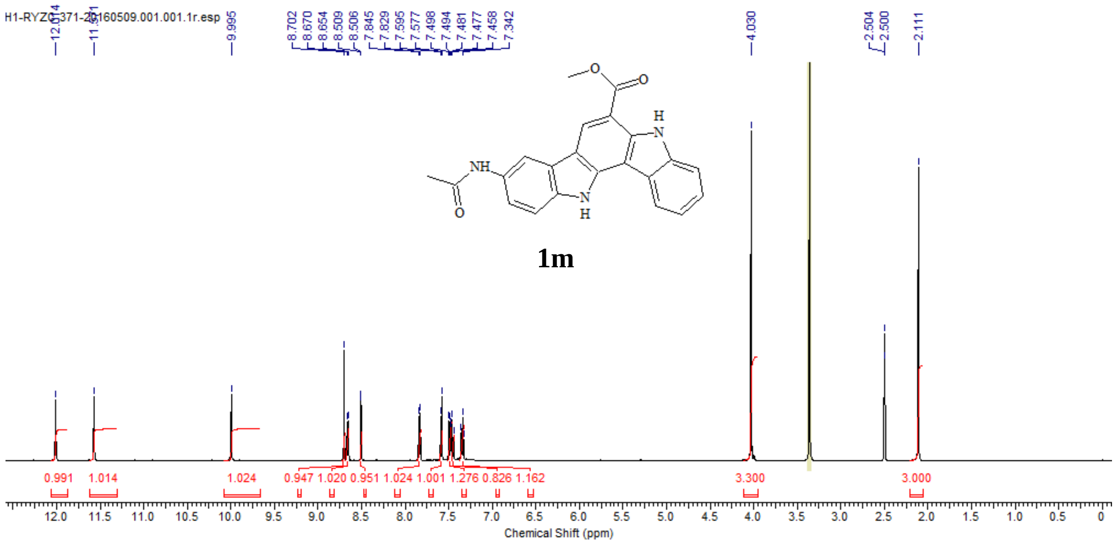
9.995
8.702 8.670 8.654 8.509 8.506 7.845 7.829 7.595 7.577 7.498 7.494 7.481 7.477 7.458 7.342
4.030
2.504 2.500
2.111
NH
O
H
N
1m
0.991 1.014 1.024 0.947 1.020 0.951 1.024 1.001 1.276 0.826 1.162 3.300 3.000
Chemical Shift (ppm)

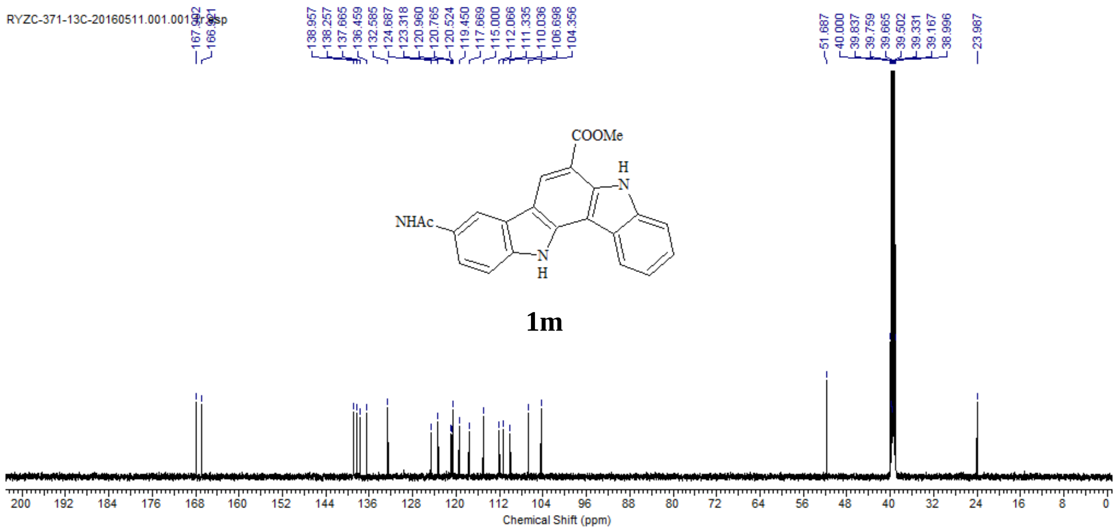
RYZC-371-13C-20160511.001.001.1r.esp
138.957
138.257
137.665
136.459
132.585
124.687
123.318
120.960
120.765
120.524
119.450
117.669
115.000
112.066
111.335
110.036
106.698
104.356
51.687
40.000
39.837
39.759
39.665
39.502
39.331
39.167
38.996
23.987
COOMe
NHAc
1m
Chemical Shift (ppm)

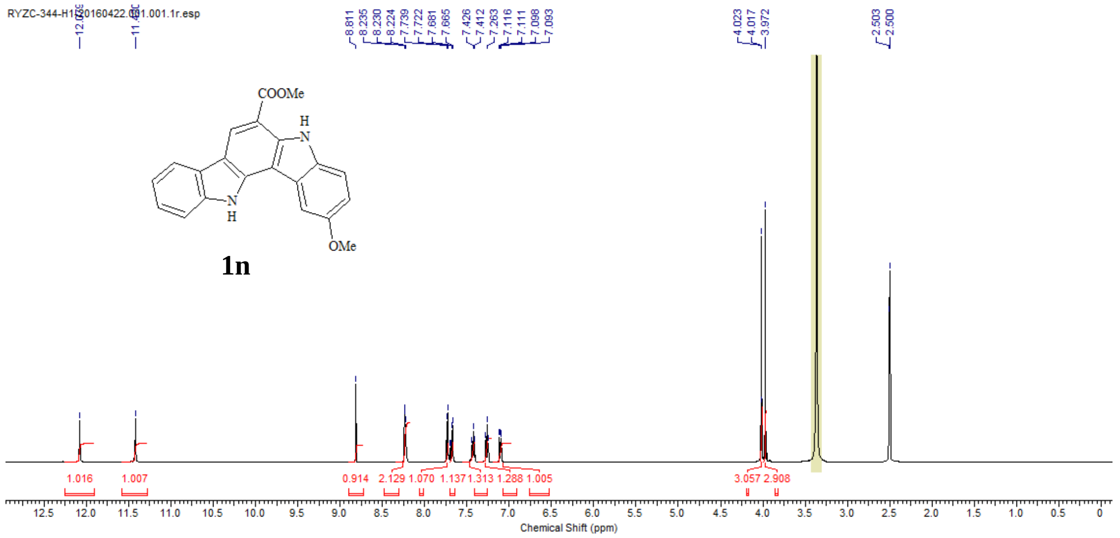
RYZC-344-H1-20160422.001.001.1r.esp
8.811
8.235
8.230
8.224
7.739
7.722
7.681
7.665
7.426
7.412
7.263
7.116
7.111
7.098
7.093
4.023
4.017
3.972
2.503
2.500
COOMe
OMe
1n
1.016
1.007
0.914
2.129
1.070
1.137
1.313
1.288
1.005
3.057
2.908
Chemical Shift (ppm)

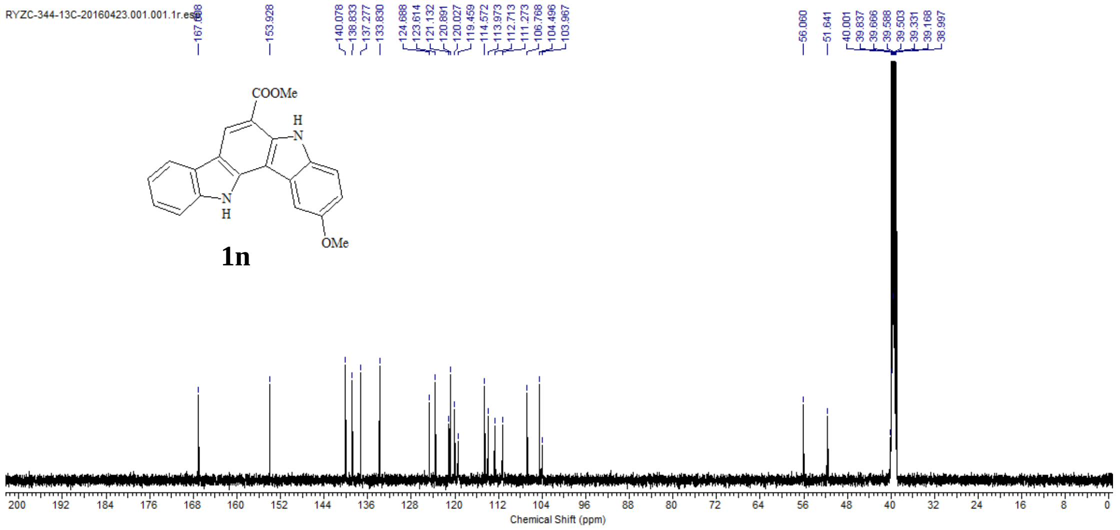
RYZC-344-13C-20160423.001.001.1r.esp
153.928
140.078
138.833
137.277
133.830
124.688
123.614
121.132
120.891
120.027
119.459
114.572
113.973
112.713
111.273
106.768
104.496
103.967
56.060
51.641
40.001
39.837
39.666
39.588
39.503
39.331
39.168
38.997
COOMe
OMe
1n
Chemical Shift (ppm)

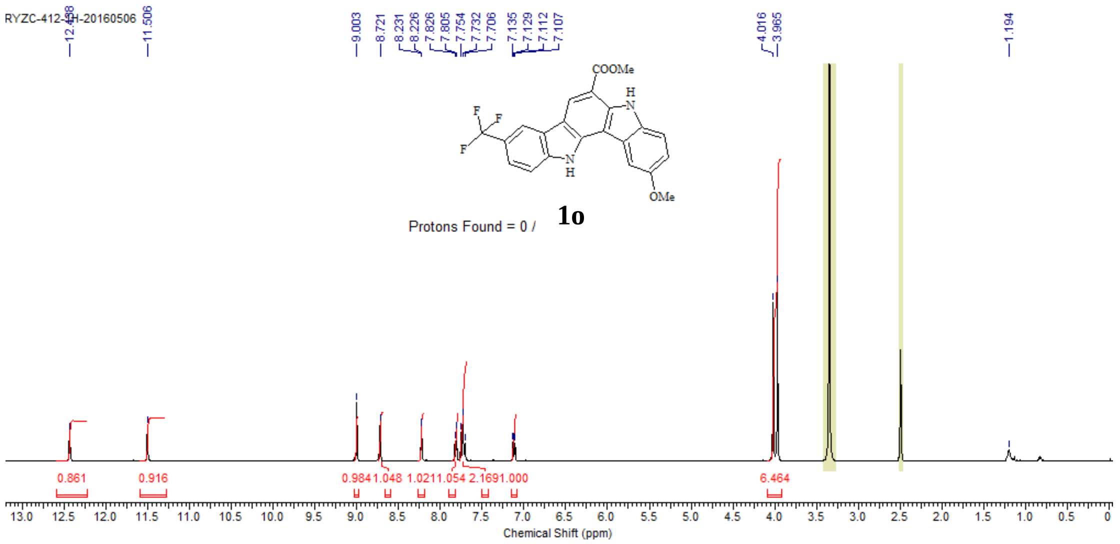

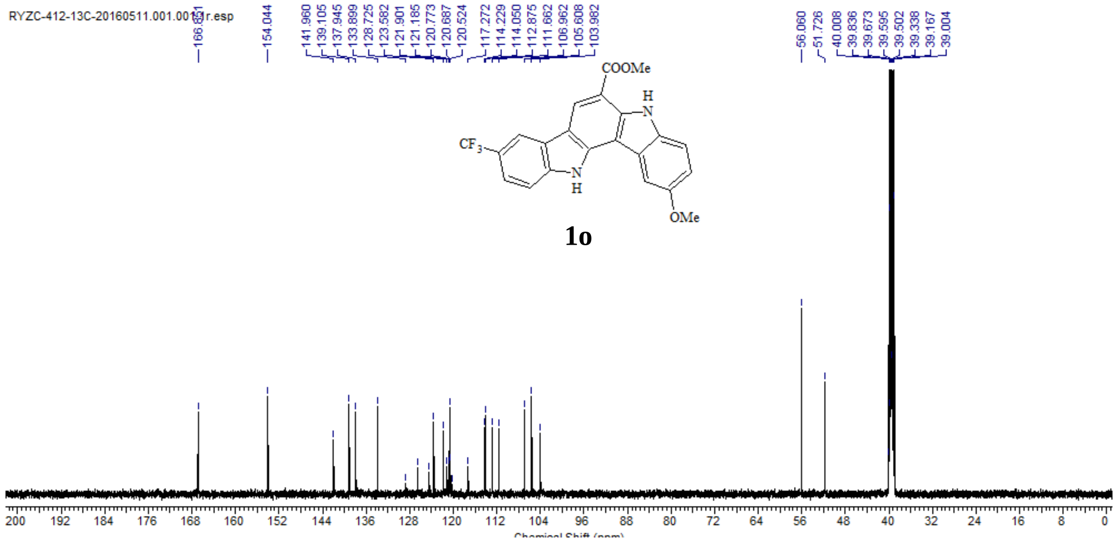

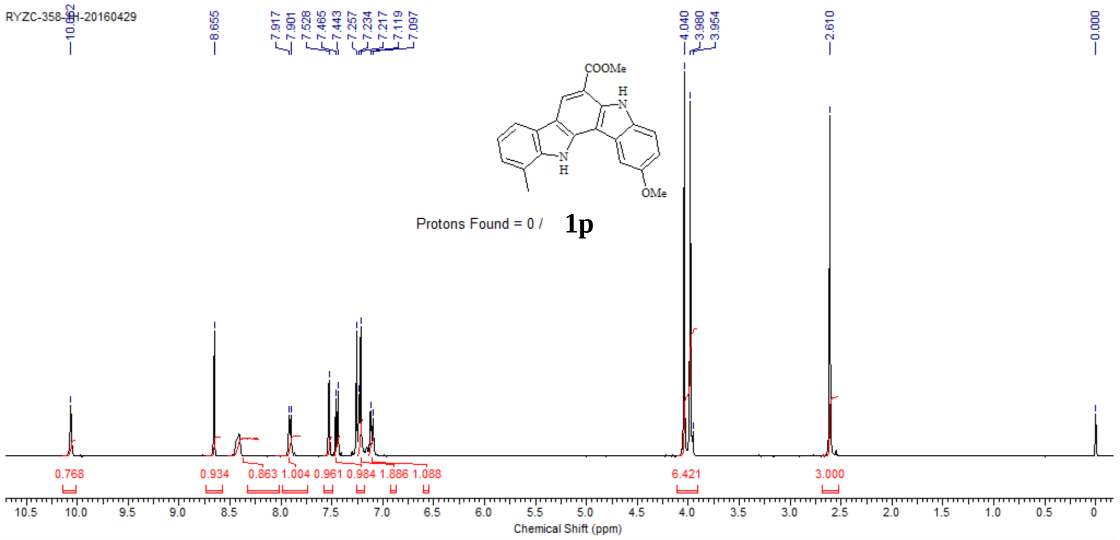

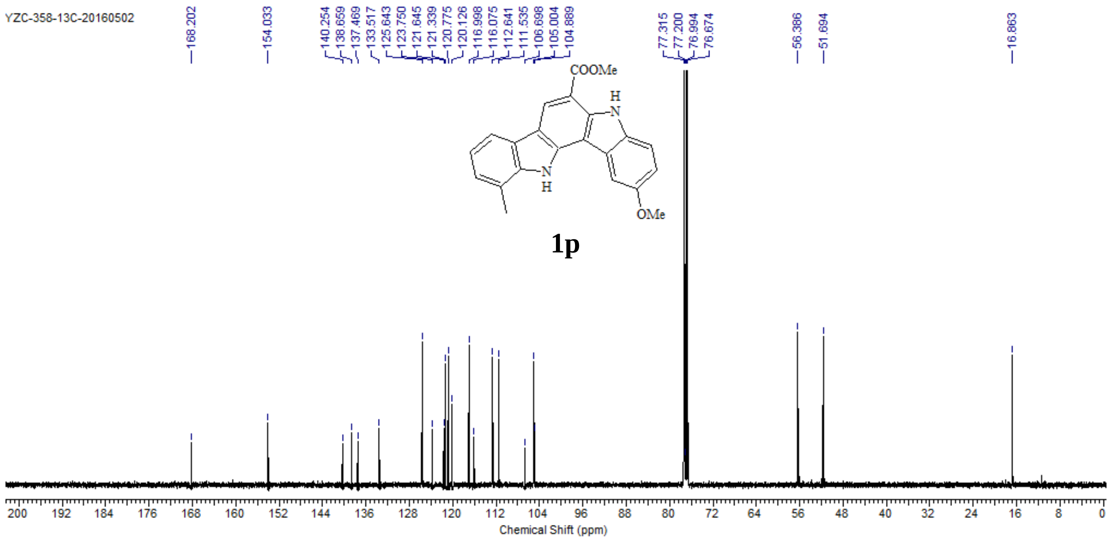
YZC-358-13C-20160502
COOMe
OMe
1p
Chemical Shift (ppm)
